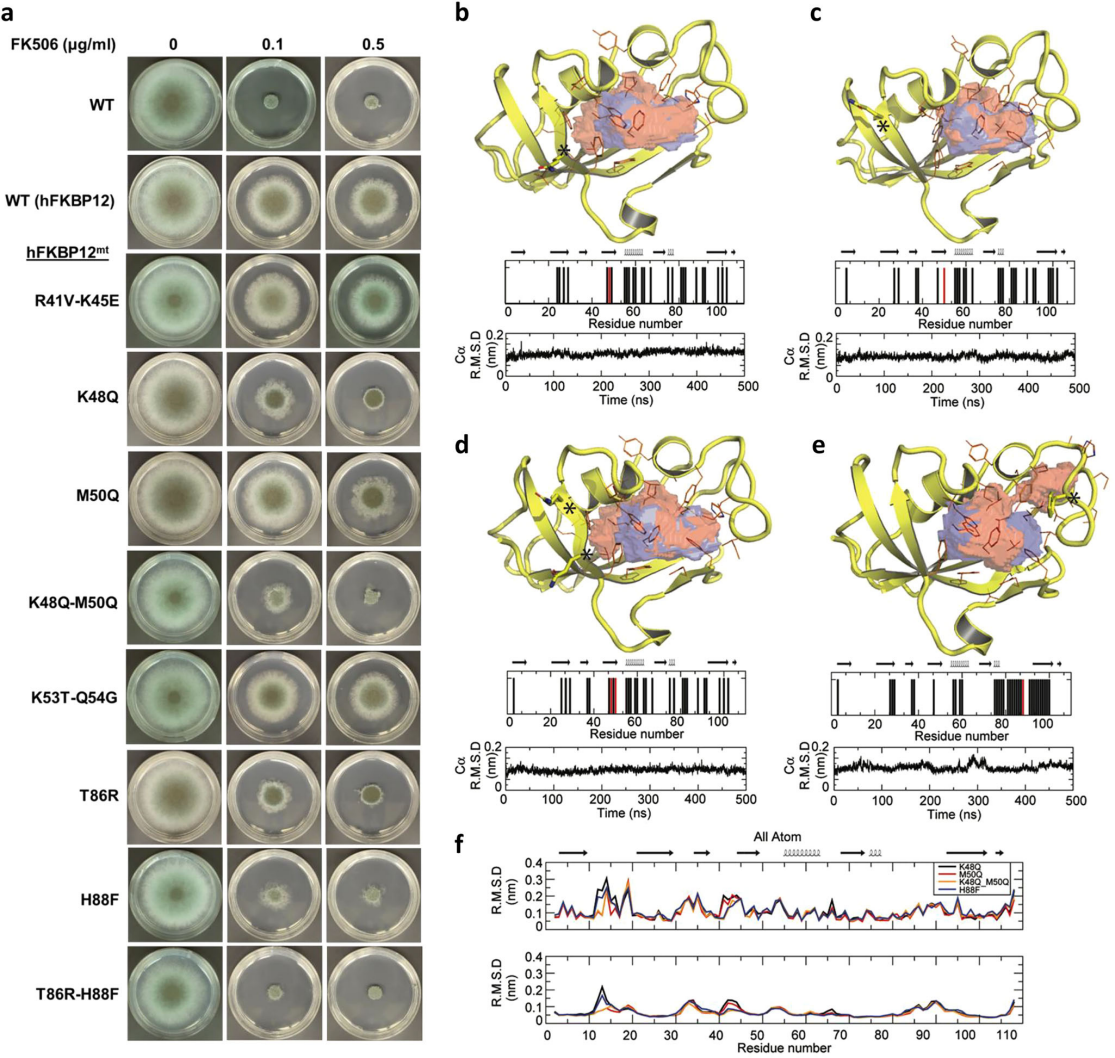# Author Correction: Harnessing calcineurin-FK506-FKBP12 crystal structures from invasive fungal pathogens to develop antifungal agents

**DOI:** 10.1038/s41467-025-61584-6

**Published:** 2025-07-03

**Authors:** Praveen R. Juvvadi, David Fox, Benjamin G. Bobay, Michael J. Hoy, Sophie M. C. Gobeil, Ronald A. Venters, Zanetta Chang, Jackie J. Lin, Anna Floyd Averette, D. Christopher Cole, Blake C. Barrington, Joshua D. Wheaton, Maria Ciofani, Michael Trzoss, Xiaoming Li, Soo Chan Lee, Ying-Lien Chen, Mitchell Mutz, Leonard D. Spicer, Maria A. Schumacher, Joseph Heitman, William J. Steinbach

**Affiliations:** 1 Division of Pediatric Infectious Diseases, Department of Pediatrics, Duke University Medical Center, Durham, NC 27710 USA; 2 Beryllium Discovery Corp., 7869 NE Day Road West, Bainbridge Island, WA 98110 USA; 3 UCB Pharma., 7869 NE Day Road West, Bainbridge Island, WA 98110 USA; 4 Seattle Structural Genomics Center for Infectious Disease (SSGCID), Seattle, WA USA; 5 Duke University NMR Center, Duke University Medical Center, Durham, NC 27710 USA; 6 Department of Biochemistry, Duke University, Durham, NC 27710 USA; 7 Department of Radiology, Duke University, Durham, NC 27710 USA; 8 Department of Molecular Genetics and Microbiology, Duke University Medical Center, Durham, NC 27710 USA; 9 Department of Immunology, Duke University Medical Center, Durham, NC 27710 USA; 10 Amplyx Pharmaceuticals, 3210 Merryfield Row, San Diego, CA 92121 USA; 11 Forge Therapeutics, Inc., 10578 Science Center Drive, San Diego, CA 92121 USA; 12 South Texas Center for Emerging Infectious Diseases, Department of Biology, The University of Texas at San Antonio, San Antonio, TX 78249 USA; 13 Department of Plant Pathology and Microbiology, National Taiwan University, Taipei, 10617 Taiwan; 14 Genentech Inc., 1 DNA Way, San Francisco, CA 94080 USA

Correction to: *Nature Communications* 10.1038/s41467-019-12199-1, published online 19 September 2019

In the version of the article initially published, in Fig. 4a, the first and second images in row “K53T-Q54G” were inadvertently duplicated from the corresponding images in row “R14V-K45E”. The corrected Fig. 4 is available as Fig. 1, below. This notice serves to amend the error.

Fig. 1 | Corrected Fig. 4