# Supplementary material for: Harnessing calcineurin-FK506-FKBP12 crystal structures from invasive fungal pathogens to develop antifungal agents
Source: Nat Commun. 2019 Sep 19;10:4275. doi: 10.1038/s41467-019-12199-1 (PMC6753081; doi:10.1038/s41467-019-12199-1)
Supplement: Supplementary file 1 — Supplementary Information [file 41467_2019_12199_MOESM1_ESM.pdf]

Supplementary Information

Harnessing Calcineurin-FK506-FKBP12 Crystal Structures from Invasive Fungal Pathogens to Develop Antifungal Agents

Praveen R. Juvvadi*[1], David Fox III[2,3,4], Benjamin G. Bobay[5,6,7], Michael J. Hoy[8], Sophie M. Gobeil[6,7], Ronald A. Venters[5,6,7], Zanetta Chang[8], Jackie J. Lin[8], Anna Floyd Averette[8], D. Christopher Cole[1], Blake C. Barrington[1], Joshua D. Wheaton[9], Maria Ciofani[9], Michael Trzoss[10], Xiaoming Li[10,11], Soo Chan Lee[12], Ying-Lien Chen[13], Mitchell Mutz[10,14], Leonard D. Spicer[5,6,7], Maria A. Schumacher[6], Joseph Heitman[8], and William J. Steinbach*[1,8]

Supplementary Figure 1

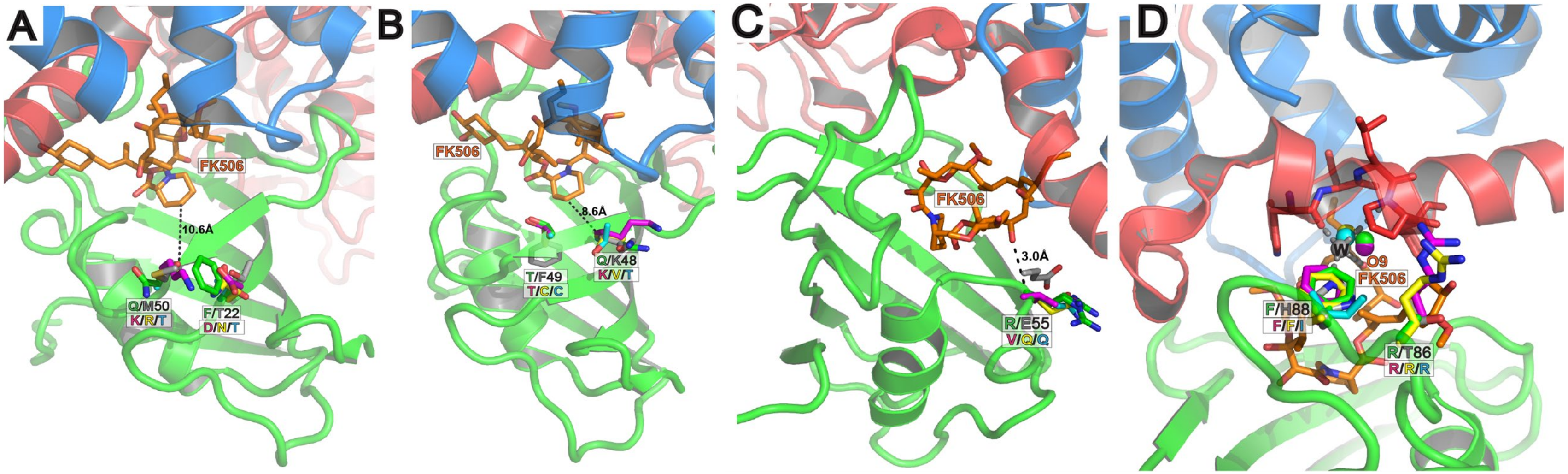

E

F22T, Q50M, R55E

```
AfFKBP12      MGVTKELKSPGNGVDFPKKGDFVTIHYTGRLTDGSKFDSSVDRNEPFQTQIGTGRVIKGW
 HFKBP12      MGVQVETISPGDGRTFPKRGQTCVVHYTGMLEDGKKFDSSRDRNKPFKFMLGKQEVIRGW
              ***  *  ***:*  ***:*:  .:**** * **.***** ***:**:  :*. .**:**
```

F88H

```
AfFKBP12      DEGVPQMSLGEKAVLTITPDYGYGARGFPPVIPGNSTLIFEVELLGINNKRA
 HFKBP12      EEGVAQMSVGQRAKLTISPDYAYGATGHPGIIPPHATLVFDVELLKLE----
              :***.***:*::* ***:***.*** *.* :** ::**:*:**** ::
```

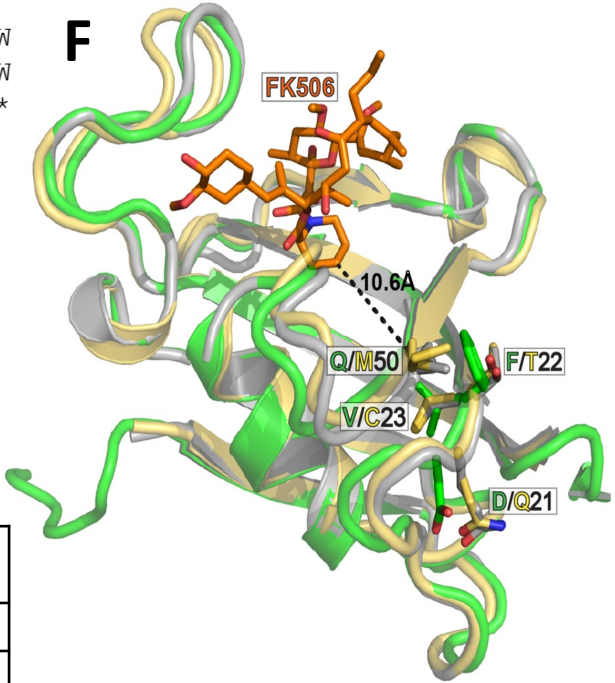

G

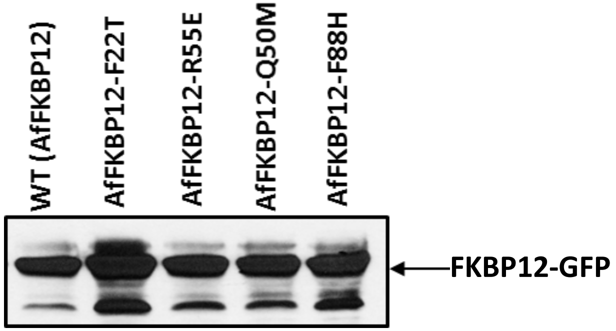

H

FK506 (µg/ml)	AfFKBP12-F88H Septal Localization
0.1	0%
0.5	17%
1	29%

Supplementary Figure 1. Differentiation between bovine and fungal FKBP12s and mutation of AfFKBP12. Overlay of the crystal structures of the fungal CNs with the bovine complex (PDB: 1TCO) at the *A. fumigatus* mutated positions (A. F22; Q50, B. Q48; T49, C. R55, and D. R86; F88). Amino acids side chains are colored according to *A. fumigatus* in green, bovine in gray, *C. albicans* in cyan, *C. immitis* in magenta and *C. neoformans* in yellow. *A. fumigatus* CN is shown as reference and colored as CnA red, CnB blue, FKBP12 green, and FK506 in orange. In panel D, W indicates a water molecule coordinated (dash lines) with FK506-O9. (E) Clustal alignment of AfFKBP12 and human FKBP12. Various mutations performed in AfFKBP12 residues are indicated by black arrows and boxed in green. Chemical shifts observed in AfFKBP12/ hFKBP12 upon binding FK506 are shown as red dashed arrows or blue dashed arrows, respectively. (F) Overlay of crystal structures *A. fumigatus* (PDB: 5HWB) and bovine FKBP12s (PDB: 1TCO) showing FK506. AfFKBP12 is shown in green, hFKBP12 in yellow, and FK506 is in orange. (G) Western detection of the *A. fumigatus* WT and mutated FKBP12 proteins using anti-GFP antibodies. (H) Quantification of septal localization of the AfFKBP12-F88H protein in the presence of varying concentrations of FK506. Note that T49, F88, and V91 residues at the FKBP12-FK506 interface are not conserved between mammalian and fungal species and mediate interactions through their side-chains. T49 does not directly contact FK506, but is important for interactions with the 40s loop that approaches the BBH. The F88 and V91 residues pack directly against the BBH, leading to the burial of key hydrophobic residues between FKBP12, FK506, and CnA. Notably, *A. fumigatus* and *C. neoformans* Phe88 (*C. immitis* Phe96; *C. albicans* Ile102) differs from the hFKBP12 His88 side chain by the latter's ability to contribute to the coordination of a conserved water molecule.

Supplementary Figure 2

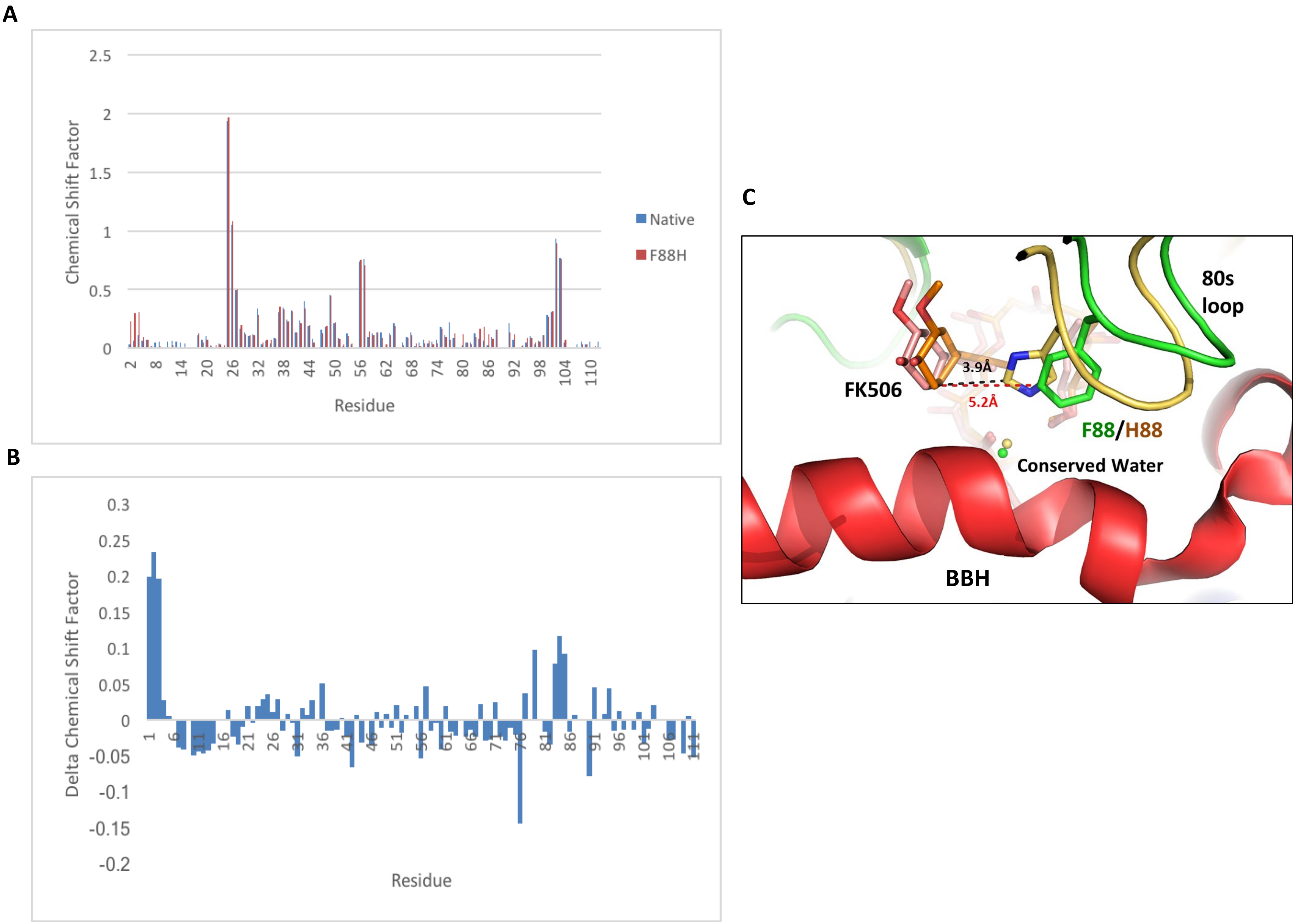

Supplementary Figure 2. Comparison of chemical shifts induced by FK506 binding to native and mutant forms of FKBP12. (A) FK506 binding to native (blue) and the F88H mutant of *A. fumigatus* FKBP12 indicate that the residues involved are essentially identical. (B) Only small changes are seen in residues G2, V3, T4, I77, D80, G84, A85 and R86 when the Chemical Shift Factor of the native protein are subtracted from the F88H mutant (Delta Chemical Shift Factor). The changes in the 80s loop very near the mutation site are to be expected. (C) 80s loop binding differences between *A. fumigatus* and mammalian FKBP12. *A. fumigatus* and mammalian complexes (1TCO.pdb) are aligned by CnB and BBH regions. Only *A. fumigatus* CnA BBH (red) and CnB (blue) are shown for clarity. *A. fumigatus* (green/orange) and mammalian (yellow/pink) FKBP12-FK506 are displayed with sticks highlighting F/H88 of the 80s loop with distances shown as dashed lines (red = fungal, black = mammalian). The conserved water bound to the BBH is shown as a non-bonded sphere colored as with FKBP12. The mammalian 80s loop packs tighter against the BBH (Δ3.2Å FKBP12 Ca Pro89 and BBH Ca Pro354/Pro378) and FK506 (Δ1.3Å FKBP12 Ca Phe88/His88 and FK506 C33) .

Supplementary Figure 3

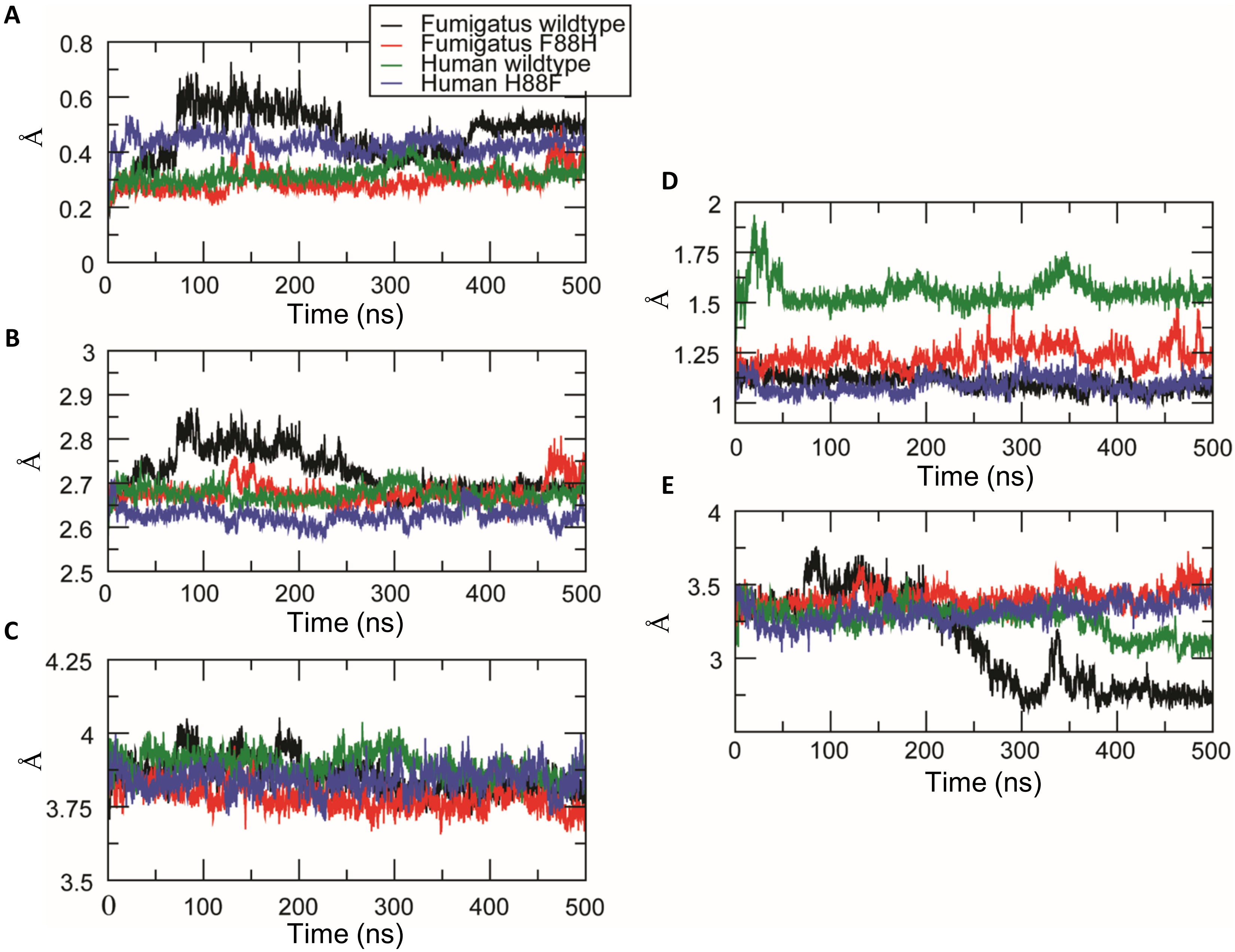

Supplementary Figure 3. Visual inspection of the MD simulation COM distances of *A. fumigatus* WT, *A. fumigatus* F88H, Human WT, and Human H88F. Panels A-D are MD simulations with *A. fumigatus* CN. (E) MD simulation comparing AfFKBP12 WT and F88H with *A. fumigatus* CN and hFKBP12 WT and H88F with Human CN. Panel A – Ca RMSD, B – radius of gyration (overall compactness of structure), C – COM distance of CnA/CnB, D – COM distance of FKBP12/FK506, and E – COM distance of CnA/FK506. *A. fumigatus* WT – black, *A. fumigatus* F88H – red, Human WT – green, and Human H88F – blue.

Supplementary Figure 4

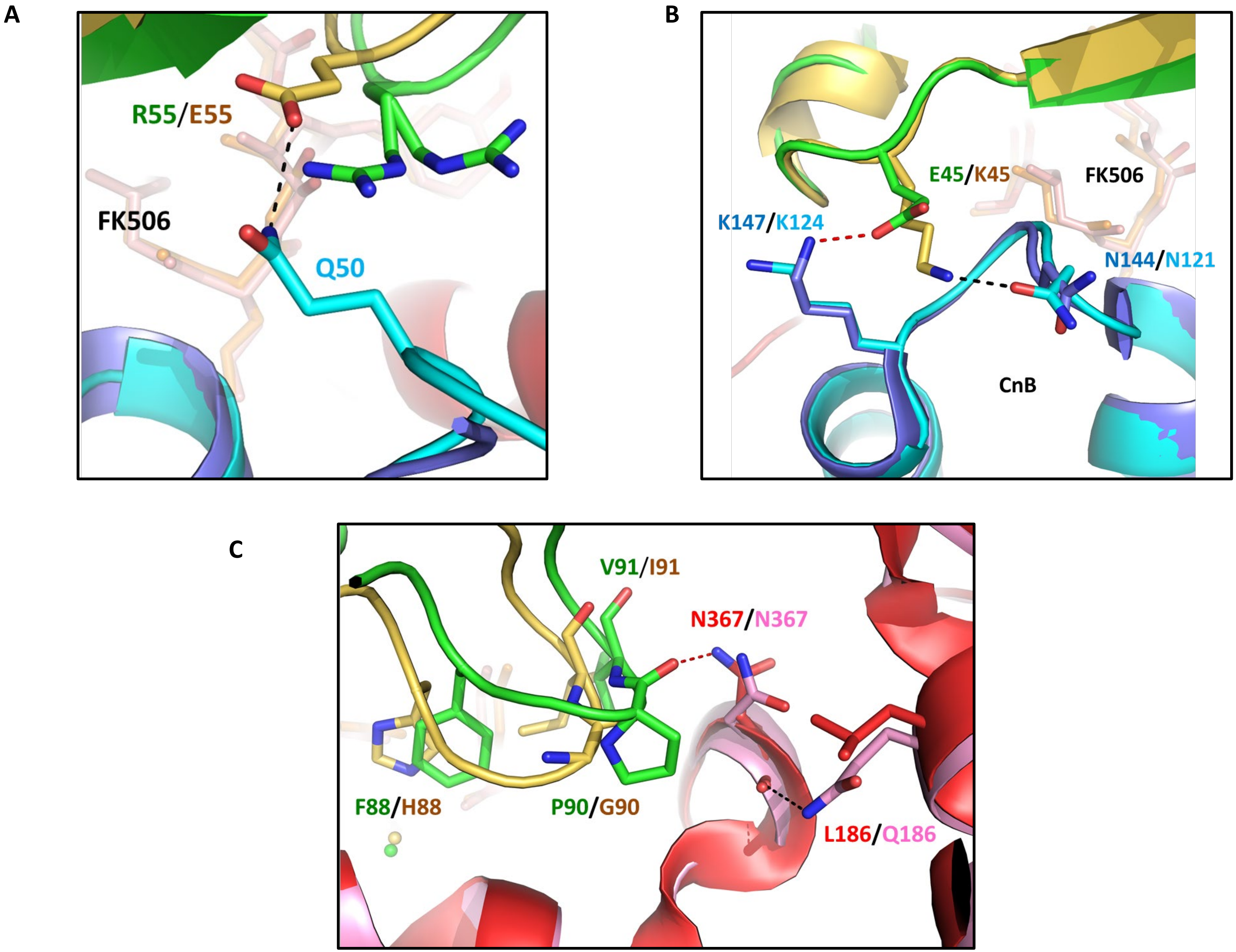

Supplementary Figure 4. Differences in fungal and mammalian FKBP12 binding interface with CN. *A. fumigatus* and mammalian complexes (1TCO.pdb) were aligned by CnB and BBH regions. *A. fumigatus* CN/FKBP12/FK506 (red/blue/green/orange) and mammalian (pink/cyan/yellow/salmon) are shown with sticks and dashed lines (red = fungal, black = mammalian) highlighting differences in interface residues between species. (A-B) FKBP12 and CnB interface; (A) mammalian FKBP12 E55 – CnB Q50 hydrogen bond is absent in the *A. fumigatus* complex crystal structure, replaced by non-interacting residues FKBP12 R55 – CnB S50 (disordered in the crystal structure). The H-bond observed at the C-terminus of the BBH in the mammalian complex (between CnB Q50 and FKBP12 E55) is absent in the *A. fumigatus* complex, in which this pair is replaced by CnB S50 and FKBP12 R55 that are poorly ordered in the structure. (B) Altered hydrogen bond pattern, mammalian FKBP12 K45 and CnB N121 interaction is lost in *A. fumigatus* due to mutation of K45 to Glu in FKBP12. *A. fumigatus* E45 picks up a salt-bridge to CnB K147, an interaction absent in the mammalian complex. A charge-swap substitution at hFKBP12 K45 (E45 in *A. fumigatus*) disrupts the H-bond with CnB N121 in the mammalian structure; however, as a result, the fungal complex forms a new salt-bridge between AfFKBP12 E45 and CnB K147 (K124 in mammalian). (C) Hydrogen bond between mammalian Q186 (CnA – catalytic domain) and main-chain carbonyl of N367 (CnA – near the BBH) is lost in *A. fumigatus* due to mutation of Q186 to Leu. *A. fumigatus* CnA N367 picks up an additional hydrogen bond to FKBP12 P90, absent in the mammalian complex where P90 is replaced by Gly. *A. fumigatus* shows an additional H-bond compared to the mammalian complex between CnA N367 (mammalian N367) and FKBP12 P90 (mammalian G90), likely due to sequence alterations in the neighboring hFKBP12 loop, at I91 (*A. fumigatus* V91), G90 (*A. fumigatus* P90), and H88 (*A. fumigatus* F88). See also Supplementary Table 2.

Supplementary Figure 5

A

```
ATGGGTGTTCAGGTGGAGACTATTTCG
CCAGGTGACGGACGGACATTTCCTAAG
CGGGGACAGACTTGTGTGGTTCATTAT
ACAGGCATGCTGGAGGACGGCAAGAAG
TTCGACTCCAGCCGCGATCGCAACAAG
CCCTTCAAGTTCATGCTCGGCAAGCAG
GAAGTCATCCGAGGATGGGAGGAAGGC
GTCGCTCAGATGTCCGTCGGACAGCGA
GCTAAGCTGACCATCTCCCCTGATTAC
GCCTACGGCGCTACCGGCCACCCTGGT
ATTATCCCTCCGCACGCCACTTTGGTG
TTTGATGTTGAACTCTTGAAGCTGGAA
```

B

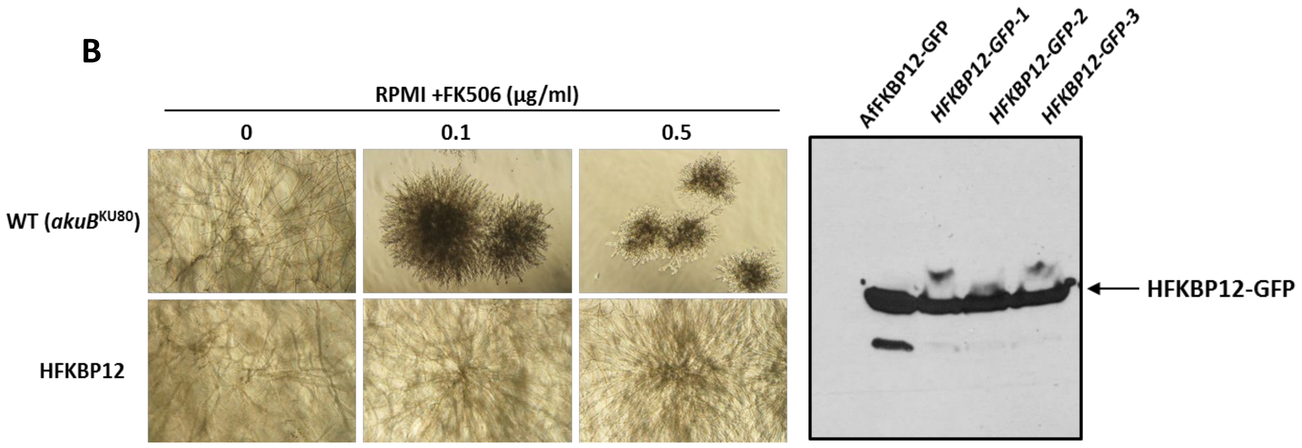

C

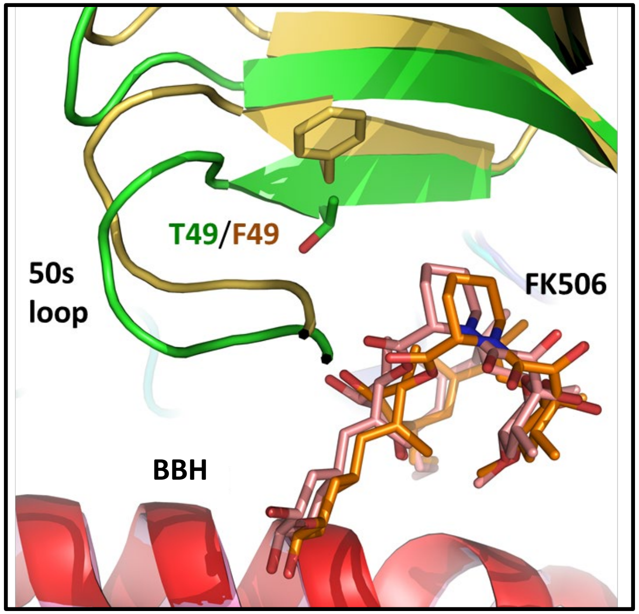

D

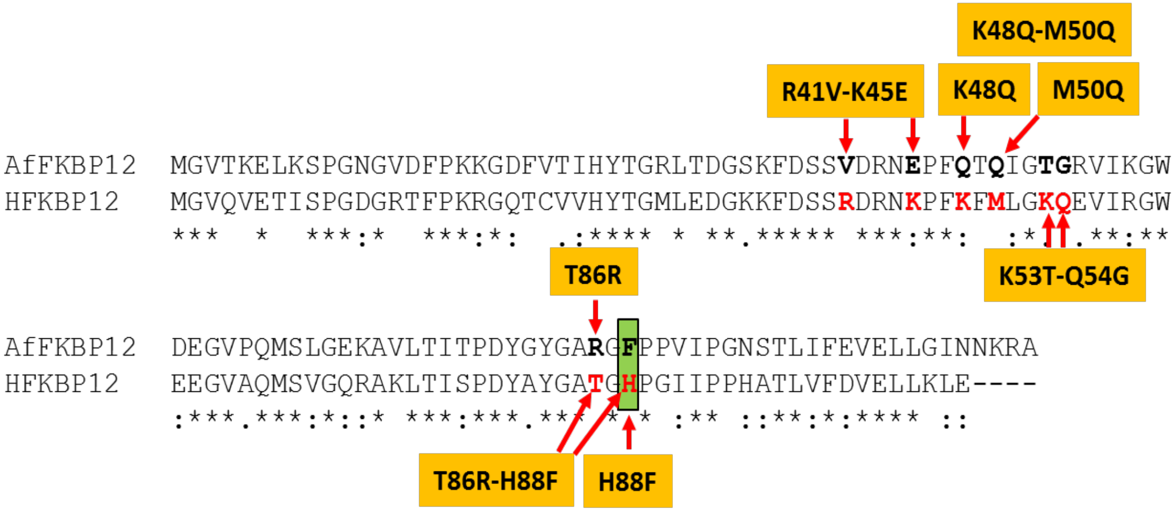

Supplementary Figure 5. Expression of Human FKBP12 in *A. fumigatus*. (A) Codon optimized hFKBP12 with 324 bp in length and a GC% of 57.08 is shown. (B) Strain expressing hFKBP12-GFP was cultured in RPMI liquid medium in the absence or presence of FK506 (0.1 and 0.5 μg/ml) for 48 h. Note the resistance of hFKBP12 expression strain to FK506 in comparison to the WT strain. (C) Western detection of hFKBP12 protein using the anti-GFP polyclonal primary antibody and peroxidase labeled anti-rabbit IgG secondary antibody. Arrow indicates the ~37 kDa FKBP12-GFP fusion proteins. (D) *A. fumigatus* and mammalian FKBP12 and BBH regions showing differences observed in the FKBP12 50s loop residues Phe49-Glu55, stemming from the Phe→Thr (F49/T49) substitution. In the FK506 pocket of FKBP12, the aromatic side-chain of mammalian F49 is replaced by the smaller more polar residue, T49, in *A. fumigatus.* (E) Clustal alignment of AfFKBP12 and hFKBP12. Positions of the various mutations are indicated.

Supplementary Figure 6

A

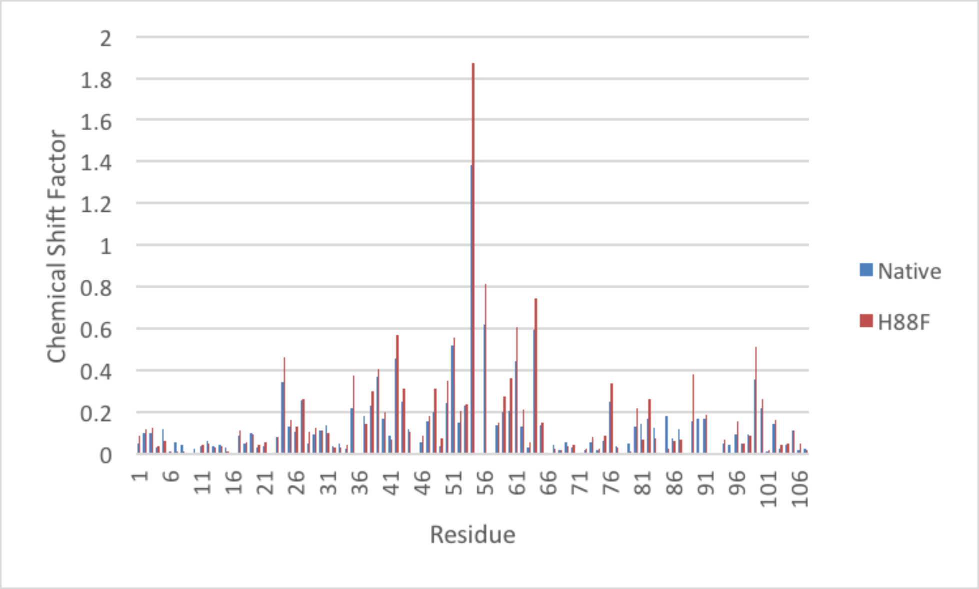

B

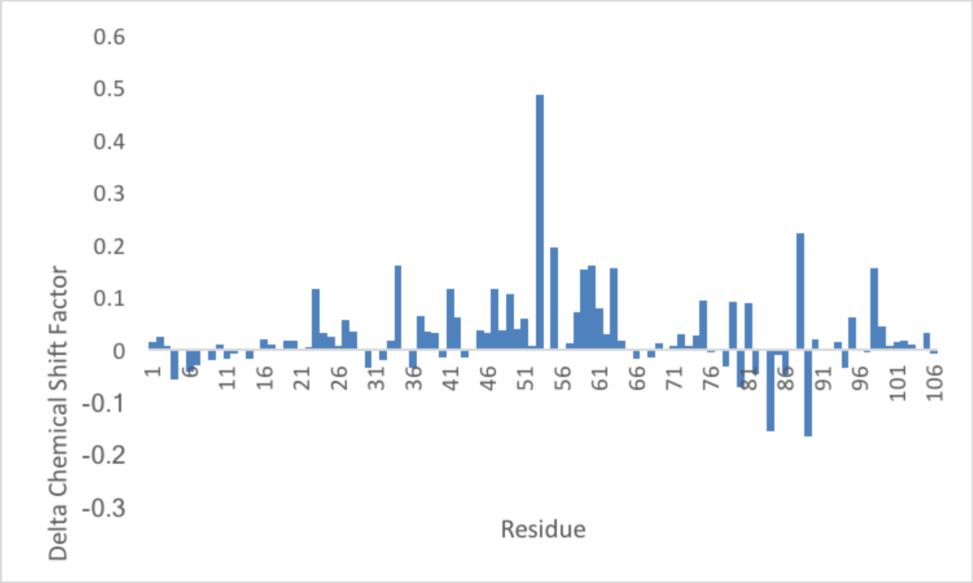

Supplementary Figure 6. Comparison of chemical shifts induced by FK506 binding to native and mutant forms of FKBP12. (A) FK506 binding to native (blue) and the H88F mutant (red) of hFKBP12 reveal a very similar binding interaction. (B) The delta chemical shift show that, again, residues Thr85, Gly89 and Ile90 in the 80s loop near the mutation site are effected as expected. Another larger difference is seen in the 50s loop at Glu54 and Ile56 perhaps as a conformational adjustment to the mutation.

Supplementary Figure 7

A

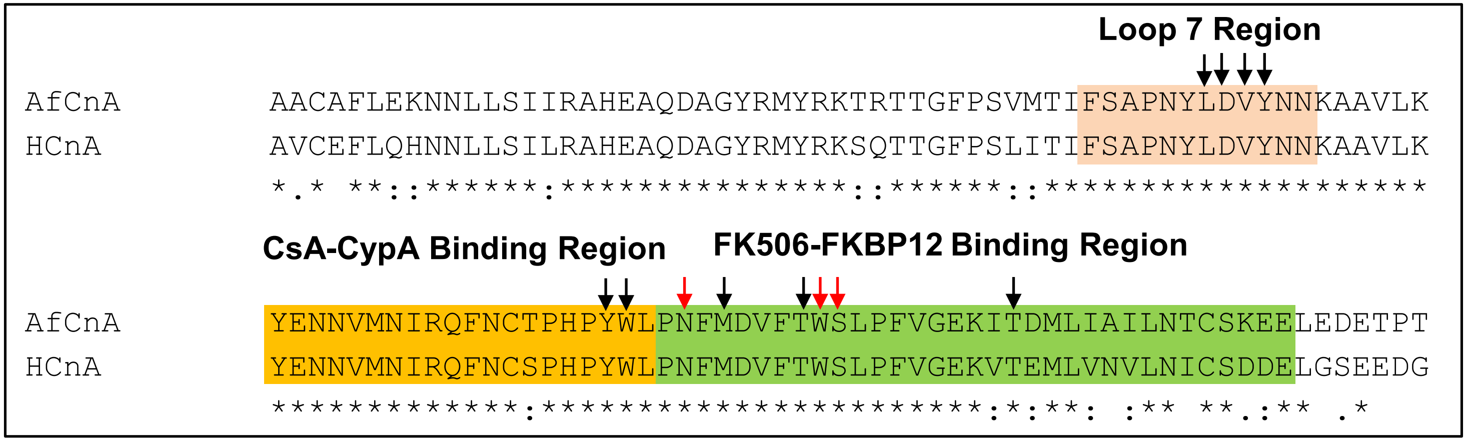

B

Loop 7 Residues (L334A–Y337A)

AfCnA Mutation	FK506 *
G145A	Sensitive
Y181A	Sensitive
L334A	Sensitive
D335A	Sensitive
V336A	Sensitive
V336R	Sensitive
Y337A	Sensitive
Y363A	Partial Resistance
Y363F	Partial Resistance
W364A	Sensitive
M369A	Partial Resistance
Y363A M369A	Increased Resistance
T373A	Sensitive
T384A	Sensitive

Supplementary Figure 7. CnA mutations confer partial FK506 resistance. (A) Partial sequence alignment of *A. fumigatus* CnA with human CnA. The three regions including the loop 7, the CsA-CypA binding domain and the FK506-FKBP12 binding domain are shaded. Arrows in black point to the important residues that were mutated in this study. Red arrows point to important residues known to induce FK506 resistance. Mutations in CnA were performed based on previous literature and also our modeling based on *A. fumigatus* CN-FKBP12-FK506 crystallization data. (B) List of mutations performed in *A. fumigatus* CnA and their susceptibility to FK506* (100ng/ml) were recorded.

Supplementary Figure 8

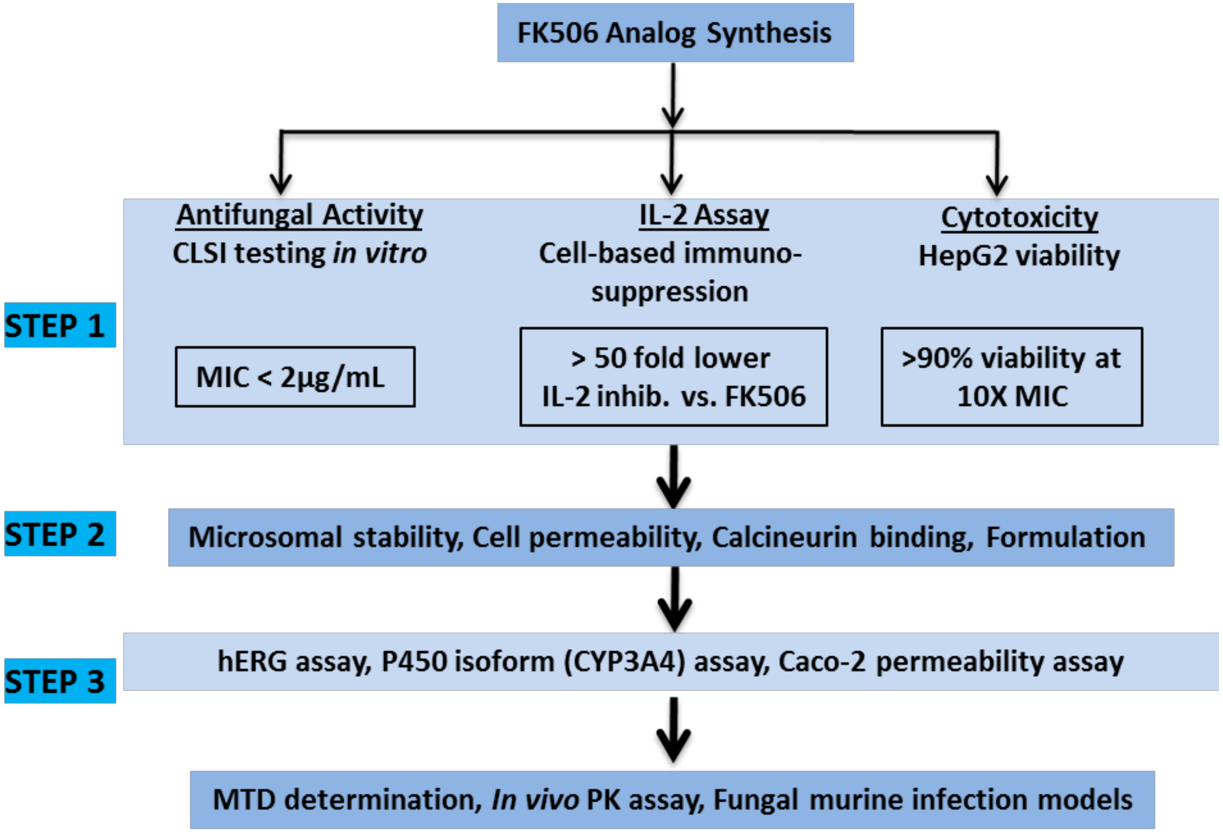

Supplementary Figure 8. Flow chart of FK506 analogs screening. Work flow involving three steps of screening the various FK506 analogs synthesized to select the effective analog for further screening in fungal murine infection models.

Supplementary Figure 9

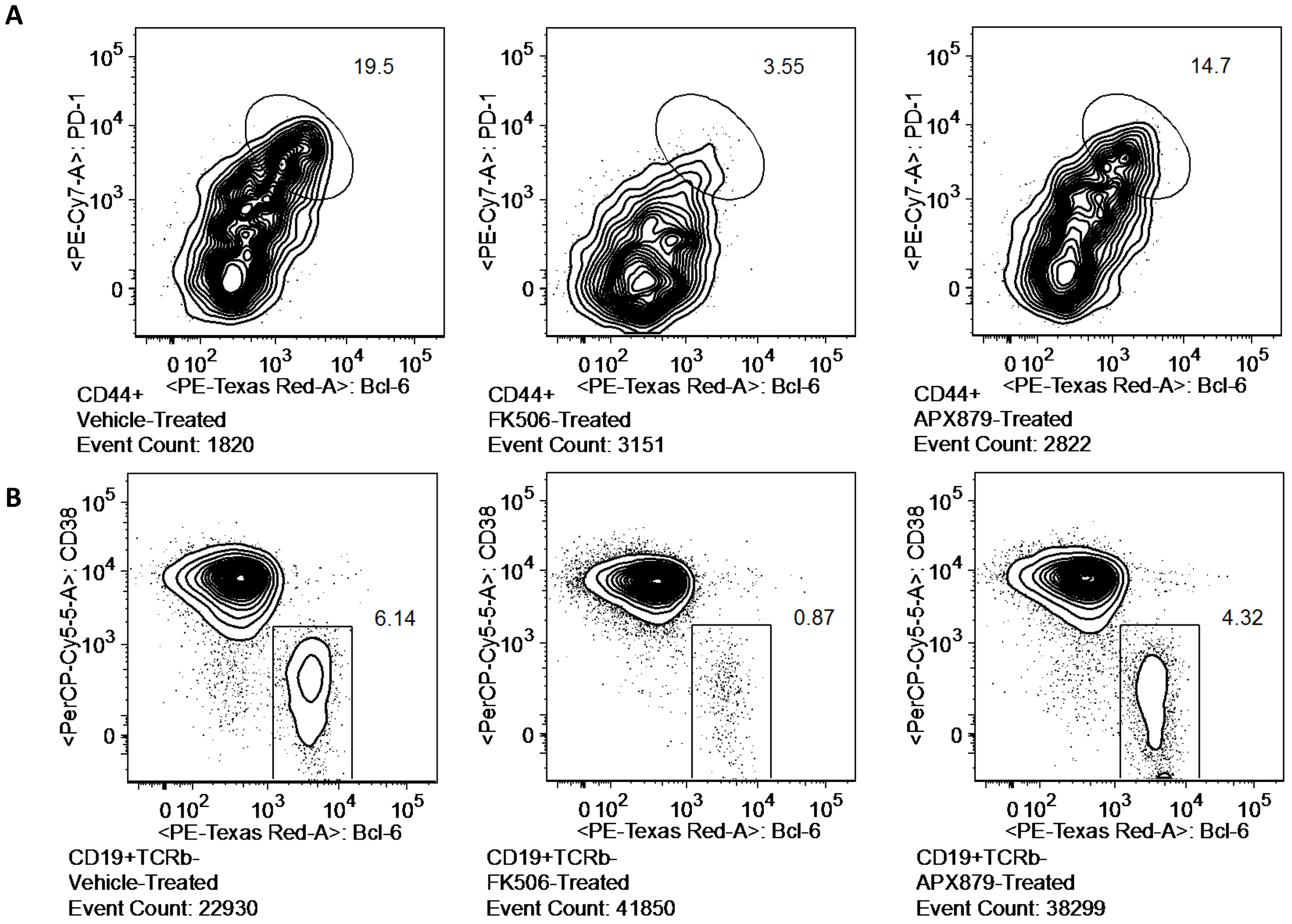

Supplementary Figure 9. ***In vivo* immunosuppression of calcineurin inhibitors.** (A) Abundance of T helper cells isolated from lymph nodes of animals immunized with NP-OVA and treated with vehicle (left), 5 mg/kg FK506 (middle), or 20 mg/kg APX879 (right). FK506 treatment results in significantly reduced T helper cell population compared to vehicle or APX879 treatment. (B) Abundance of Germinal Center (GC)-B cells isolated from lymph nodes of the same animals in (A). FK506 treatment results in significantly reduced GC-B cell population compared to vehicle or APX879 treatment.

Supplementary Figure 10

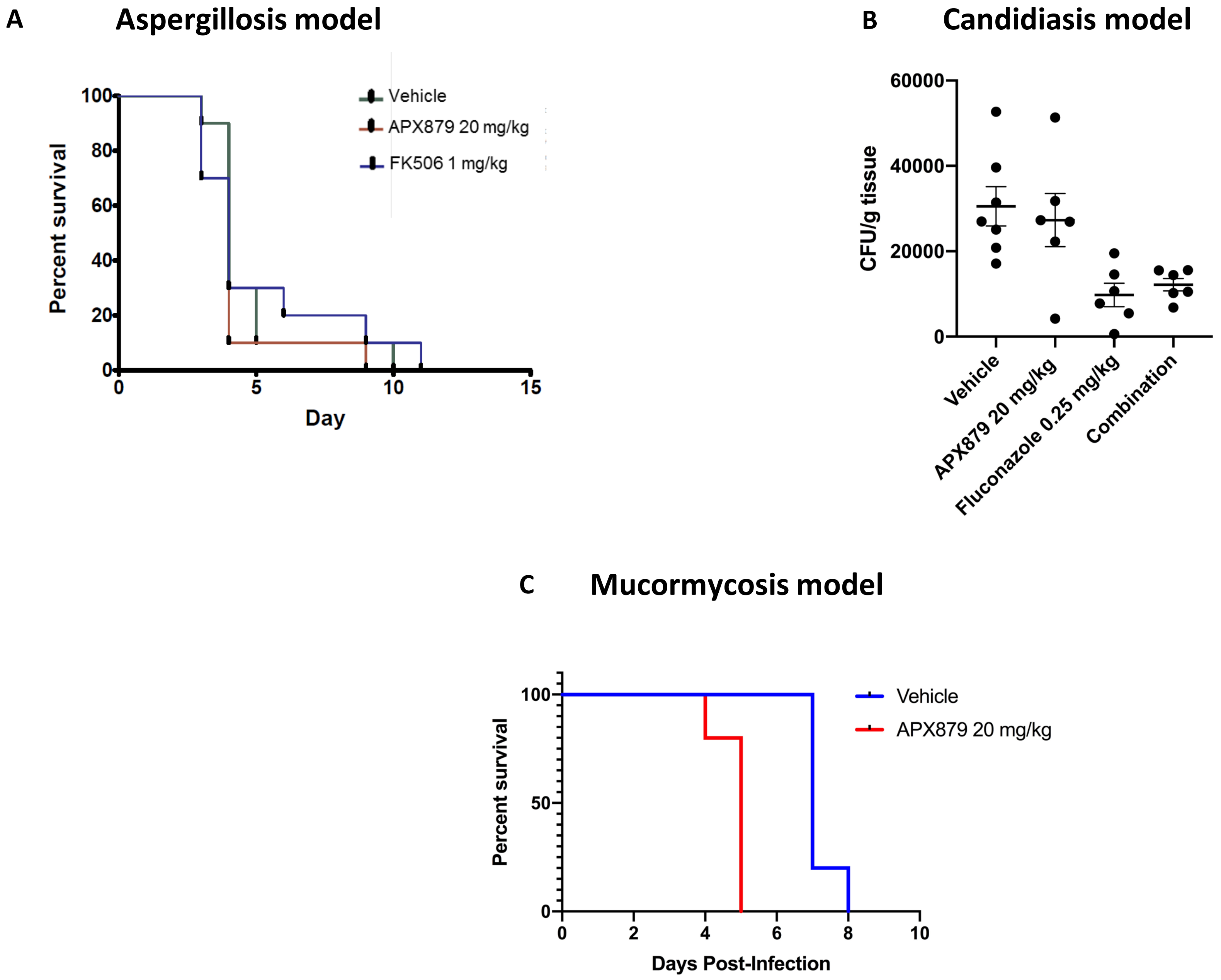

Supplementary Figure 10. APX879 was not efficacious in Aspergillosis, Candidiasis, or Mucor mycosis models of murine invasive fungal infections. (A) APX879 treatment did not improve survival in mice infected with *Aspergillus fumigatus.* CD1 mice were immunosuppressed with cyclophosphamide (150 mg/kg on day -2 and 100 mg/kg on day +3) and triamcinolone acetonide (40 mg/kg on days -1 and +6). Three groups of 20 mice each were immunosuppressed and intranasally infected with the *A. fumigatus* CEA10 strain (40 µl of $3x10^7$ spores/ml) and received either no treatment-vehicle only, or APX879 (20 mg/kg once daily IP), or FK506 (1 mg/kg once daily IP). FK506 was not delivered at APX879 equipotent dosing, due to earlier toxicity studies at higher doses (> 5 mg/kg daily). Survival was plotted on a Kaplan-Meier curve and log rank was used for pair-wise comparison of survival with statistical significance defined as a two-tailed $p < 0.05$. (B) APX879 treatment does not reduce kidney fungal burden in murine model of candidiasis. CD1 mice were infected with $7x10^5$ cells of *Candida albicans* SC5314 via retro-orbital intravenous instillation. Animals were treated with vehicle, APX879 20 mg/kg, fluconazole 0.25 mg/kg, or combination daily for 6 days following infection. Kidneys were harvested from each animal, homogenized, and plated for CFUs. Fungal burden is represented as CFUs per gram of tissue. There was no significant difference between vehicle and APX879 20 mg/kg treatment. ** $P<0.01$, * $P<0.05$. (C) APX879 treatment reduced survival of mice infected with *Mucor circinelloides f. circinelloides.* BALB/c mice were infected with *M. circinelloides f. circinelloides* by retro-orbital instillation of $1.25x10^6$ spores. Animals were treated with vehicle or APX879 20 mg/kg daily for 7 days. Animals were monitored in a blinded manner for weight and daily survival. ** indicates $P<0.005$.

Supplementary Figure 11

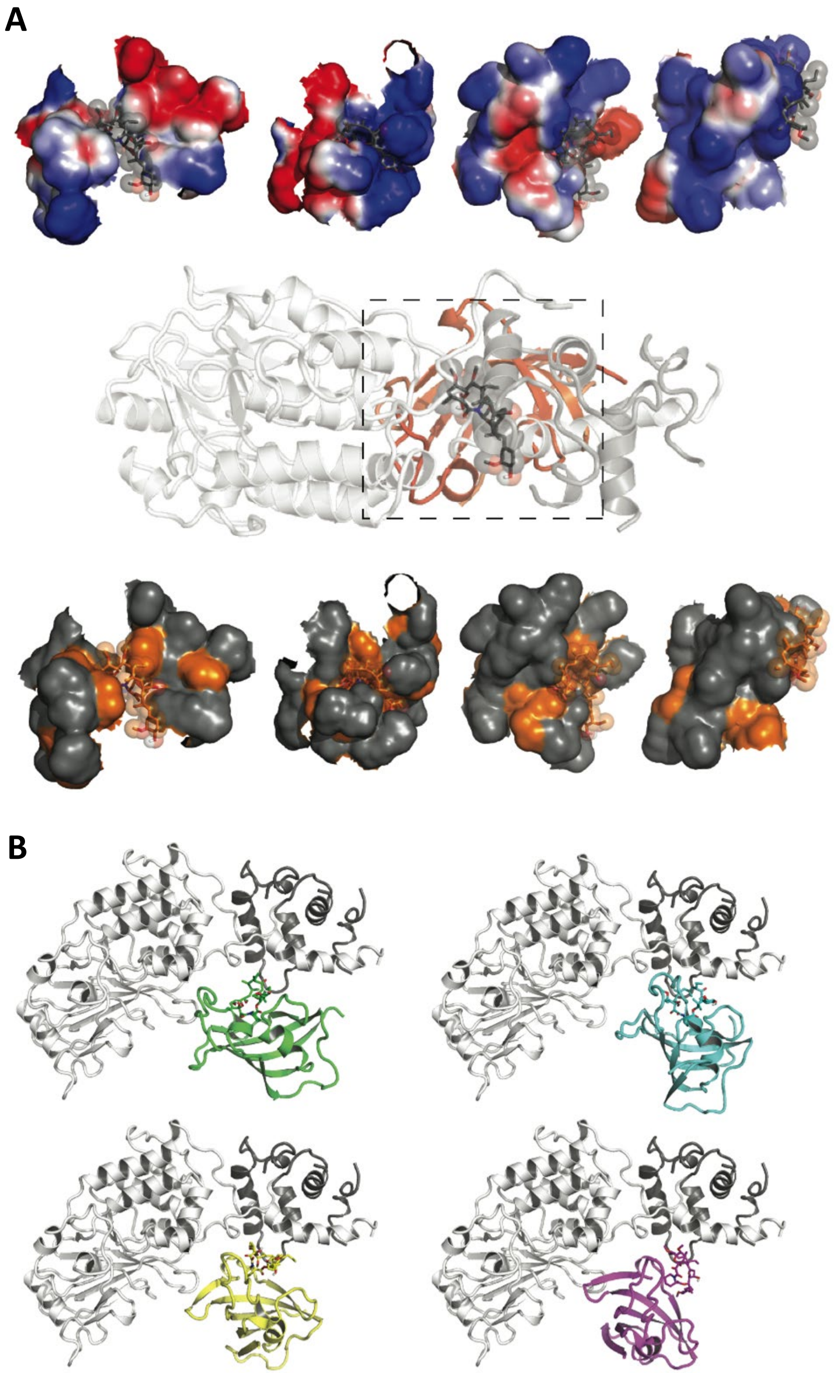

Supplementary Figure 11. FKBP12-FK506 binding to *A. fumigatus* CN. (A) (from left to right) – *A. fumigatus* wild-type, *A. fumigatus* F88H, Human wild-type, Human H88F shows the electrostatic surface potential plot contoured from −1 kT/e (red) to +1 kT/e (blue) (top) and the hydrophobic surface plot, where hydrophobic residues are colored orange and a surface rendering was accomplished, (bottom) for the interaction surface of FKBP12 to CnA/CnB with FK506 shown in stick and transparent spheres. The orientation of the surfaces is shown in the middle plot of panel A. (B) Binding conformation of A. fumigatus CnA(white)/CnB(black) complex to FKBP12:FK506 A. fumigatus wild-type (top left), A. fumigatus F88H (top right), Human wild-type (bottom left), and Human H88F (bottom right) with FK506 shown in stick format.

Supplementary Table 1.

Identity between Mammalian and Fungal Calcineurin A Catalytic Domains

**Species**	***Ca***	***Hs***	***Cn***	***Af***	***Ci***
Candida albicans (Ca)	100.00	61.92	66.77	67.38	68.00
Homo sapiens (Hs)	61.92	100.00	71.38	71.69	72.92
Cryptococcus neoformans (Cn)	66.77	71.38	100.00	83.18	84.40
Aspergillus fumigatus (Af)	67.38	71.69	83.18	100.00	92.35
Coccidiodes immitis (Ci)	68.00	72.92	84.40	92.35	100.00

Supplementary Table 2.
FKBP12 and Calcineurin Interface Details

Mammalian			*A. fumigatus*		
FKBP12					
Residue Type	Residue #	Bond Type	Residue Type	Residue #	Bond Type
THR	28		THR	28	
			ARG	30	
ASP	33		ASP	33	H
LYS	35	H	SER	35	
LYS	36	H	LYS	36	H
PHE	37		PHE	37	
ASP	38		ASP	38	
ARG	41		VAL	41	
ASP	42		ASP	42	
ARG	43	H	ARG	43	H
ASN	44		ASN	44	H
LYS	45	H	GLU	45	S
PHE	47		PHE	47	
LYS	48	H	GLN	48	H
LYS	53				
GLN	54				
GLU	55	H	ARG	55	
THR	86				Disordered
HIS	88		PHE	88	
PRO	89		PRO	89	
GLY	90		PRO	90	H
ILE	91		VAL	91	H
Calcineurin A					
			ARG	144	
			TYR	146	
TYR	181	H	TYR	181	H
PHE	182		PHE	182	
			LEU	186	
LEU	334	H	LEU	334	H
ASP	335		ASP	335	
VAL	336		VAL	336	
			TYR	337	
TYR	363	H	TYR	363	H
PRO	366		PRO	366	
ASN	367		ASN	367	H
MET	369		MET	369	
THR	373		THR	373	
TRP	374		TRP	374	
PRO	377		PRO	377	
PHE	378		PHE	378	
LYS	382		LYS	382	
GLU	385				
Calcineurin B					
GLU	47				Disordered
GLN	50	H	SER	73	
ASN	121	H	ASN	144	
ASN	122	H	ASN	145	H
LEU	123		LEU	146	
LYS	124		LYS	147	HS
GLN	127	H	GLN	150	H
			ASN	181	
LEU	159				Disordered
ILE	161				Disordered
LYS	164				Disordered

Interface residues determined in qtPISA for FKBP12 and CnA/CnB for mammalian (1TCO.pdb) and *A. fumigatus* complexes.
Residues marked in red indicate interest highlighted in figures. H = hydrogen bond; S = Salt bridge
Reference: E. Krissinel (2015) Stock-based detection of protein oligomeric states in jsPISA, Nucl. Acids Res. Doi: 10.1093/nar/gkv314

Supplementary Table 3.
FK506 Analogs with Substitutions at C22 Position and their Antifungal Activity

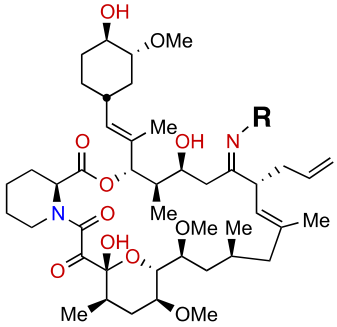

APX ID	R	IL2 IC_{50} (nM)	*A. fumigatus* MEC (μg/ml)	*C. neoformans* MIC (μg/ml)	APX ID	R	IL2 IC_{50} (nM)	*A. fumigatus* MEC (μg/ml)	*C. neoformans* MIC (μg/ml)
**786**	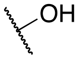	<10	n.d.	n.d.	**926**	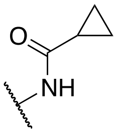	>10*	4	2
**800**	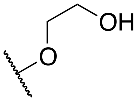	<10	n.d.	n.d.	**929**	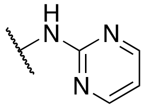	>10*	4	1
**802**	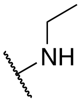	<10	n.d.	n.d.	**938**	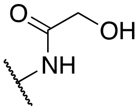	<10	n.d.	n.d.
**831**	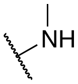	<10	n.d.	n.d.	**947**	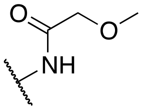	<10	n.d.	n.d.
**833**	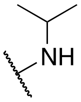	<10	n.d.	n.d.	**948**	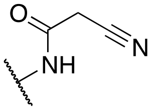	~10	2	1
**879**	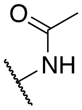	25	0.5	0.5	**962**	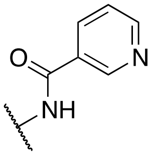	~10	4	2
**880**	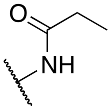	52	1	0.5	**963**	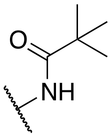	>10*	4	2
**921**	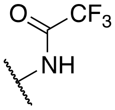	<10	n.d.	n.d.	**965**	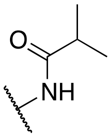	>10*	2	1

n.d. = not determined

* IC_{50} not determined

Supplementary Table 4.
Antifungal Susceptibility Testing of FK506 and APX879 against Different Pathogenic Fungi

Strain	FK506 (µg/ml)	APX879 (µg/ml)
Aspergillus fumigatus Wild type (AF293)	0.0156	0.5
Aspergillus fumigatus Wild type (CEA10)	0.0312	1
Candida albicans Wild type (SC5314)	0.06	8
Cryptococcus neoformans Wild type (H99)	0.06	1
Mucor circinelloides f. lusitanicus	0.125	4
Mucor circinelloides f. circinelloides	0.25	2

Supplementary Table 5.
Pathogenic Fungal Strains and Mutants and Susceptibility to FK506 and APX879

Strain	FK506 (µg/ml)	APX879 (µg/ml)
Aspergillus fumigatus[1]		
Wild type (AF293)	S (0.0156)	S (0.5)
Wild type (CEA10)	S (0.0312)	S (1.0)
Δ*fkbp12* (*akuB*^{KU80})	R (>5)	R (>8)
*Cryptococcus neoformans**		
Wild type (H99)	S (0.06)	S (1.0)
*frr1*Δ (MCC1)	R (>25)	R (>25)
Wild type (JEC21)	S (0.06)	S (1.0)
frr1-1 (C20F1)	R (>25)	R (>25)
frr1-2 (C20F2)	R (>25)	R (>25)
CNB1-1 (C21F2)	R (>25)	R (>25)
frr1-3 (C21F3)	R (>25)	R (>25)
*Candida albicans**		
Wild type (SC5314)	S (0.06)	S (8.0)
CNB1-1/CNB1 (YAG237)	R (>25)	R (>100)
*rbp1*Δ/*rbp1*Δ (YAG171)	R (>25)	R (>100)
Mucor circinelloides f. lusitanicus		
Wild type (R7B)	S (0.125)	S (4.0)
*fkbA*Δ (RBM1)[1]	R (>2)	R (>32)
fkbA-1 (SCV33)	R (>25)	R (>25)
fkbA-2 (SCV41)	R (>25)	R (>25)
CNBR -1(MSL11)	R (>25)	R (>25)

*Disk diffusion halo assay was used to detect sensitivity or resistance.
[1]RPMI cultures were used for testing drug sensitivity.
S-Sensitive (growth inhibition zone present); R-Resistant (no zone of inhibition present)

Supplementary Table 6.
FIC Index values of FK506 and APX879 in combination with other Antifungal Drugs on Different Pathogenic Fungi

Strain	Calcineurin Inhibitor (A)	Antifungal (B)	MIC (A)	MIC (B)	FIC Index
***Aspergillus fumigatus* (CEA10)**	FK506	Ambisome	0.0312	1	0.187
	APX879	Ambisome	1	1	0.187
	FK506	Caspofungin	0.0312	1	0.218
	APX879	Caspofungin	1	1	0.375
	FK506	Voriconazole	0.0312	0.25	≤2
	APX879	Voriconazole	1	0.25	≤2
***Candida albicans* (SC5314)**	FK506	Amphotericin B	0.06	0.25	0.266
	APX879	Amphotericin B	8	0.25	0.254
	FK506	Caspofungin	0.06	0.25	0.375
	APX879	Caspofungin	8	0.25	0.375
***Cryptococcus neoformans* (H99)**	FK506	Amphotericin B	0.06	0.25	0.265
	APX879	Amphotericin B	1	0.25	0.258
Mucor circinelloides f. circinelloides	FK506	Ambisome	0.25	0.125	0.75
	APX879	Ambisome	2	0.125	0.625
	FK506	Isavoconazole	0.25	8	≤2
	APX879	Isavuconazole	2	8	≤2
