# Supplementary material for: Harnessing calcineurin-FK506-FKBP12 crystal structures from invasive fungal pathogens to develop antifungal agents
Source: Nat Commun. 2019 Sep 19;10:4275. doi: 10.1038/s41467-019-12199-1 (PMC6753081; doi:10.1038/s41467-019-12199-1)
Supplement: Supplementary file 3 — Description of Additional Supplementary Files [file 41467_2019_12199_MOESM3_ESM.pdf]

Description of Additional Supplementary Files

File Name: Supplementary Data 1
Description: Primers Designed for the Generation of A. fumigatus FKBP12 Mutants

File Name: Supplementary Data 2
Description: Primers Designed for the Generation of A. fumigatus CnaA Mutants

File Name: Supplementary Data 3
Description: Strains Used and Generated in the Present Study
