# Supplementary material for: Harnessing calcineurin-FK506-FKBP12 crystal structures from invasive fungal pathogens to develop antifungal agents
Source: Nat Commun. 2019 Sep 19;10:4275. doi: 10.1038/s41467-019-12199-1 (PMC6753081; doi:10.1038/s41467-019-12199-1)
Supplement: Supplementary file 7 — Reporting Summary [file 41467_2019_12199_MOESM7_ESM.pdf]

nature research

Corresponding author(s): Praveen R. Juvvadi; William J. Steinbach

Reporting Summary

Nature Research wishes to improve the reproducibility of the work that we publish. This form provides structure for consistency and transparency in reporting. For further information on Nature Research policies, see Authors & Referees and the Editorial Policy Checklist.

Statistical parameters

When statistical analyses are reported, confirm that the following items are present in the relevant location (e.g. figure legend, table legend, main text, or Methods section).

n/a	Confirmed	
☐	☒	The exact sample size (*n*) for each experimental group/condition, given as a discrete number and unit of measurement
☐	☒	An indication of whether measurements were taken from distinct samples or whether the same sample was measured repeatedly
☐	☒	The statistical test(s) used AND whether they are one- or two-sided<br>*Only common tests should be described solely by name; describe more complex techniques in the Methods section.*
☒	☐	A description of all covariates tested
☒	☐	A description of any assumptions or corrections, such as tests of normality and adjustment for multiple comparisons
☐	☒	A full description of the statistics including central tendency (e.g. means) or other basic estimates (e.g. regression coefficient) AND variation (e.g. standard deviation) or associated estimates of uncertainty (e.g. confidence intervals)
☒	☐	For null hypothesis testing, the test statistic (e.g. *F*, *t*, *r*) with confidence intervals, effect sizes, degrees of freedom and *P* value noted<br>*Give P values as exact values whenever suitable.*
☒	☐	For Bayesian analysis, information on the choice of priors and Markov chain Monte Carlo settings
☒	☐	For hierarchical and complex designs, identification of the appropriate level for tests and full reporting of outcomes
☒	☐	Estimates of effect sizes (e.g. Cohen's *d*, Pearson's *r*), indicating how they were calculated
☐	☒	Clearly defined error bars<br>*State explicitly what error bars represent (e.g. SD, SE, CI)*

Our web collection on statistics for biologists may be useful.

Software and code

Policy information about availability of computer code

Data collection	GeneComposer (Emerald Bio); GeneArt (ThermoFisher); Vector NTI 9.1 (ThermoFisher); Phaser; Molrep; Phenix; COOT; Molprobity (http://molprobity.biochem.duke.edu/); GraphPad Prism 7 (https://www.graphpad.com/); MODELLER v9.18 (https://salilab.org/modeller/); HADDOCK v2.2 (http://www.bonvinlab.org/software/haddock2.2/); GROMACS v5.0.1 (http://www.gromacs.org/)
Data analysis	NMRPipe (https://www.ibbr.umd.edu/nmrpipe/); NMRViewJ (https://www.nmrbox.org/registry/nmrviewj); PINE (http://i-pine.nmrfam.wisc.edu/); AutoAssign (http://nmr.cabm.rutgers.edu/autoassign/cgi-bin/aaenmr.py); qtPISA; PyMOL Molecular Graphics System (Schrödinger, LLC); ProFit (http://www.bioinf.org.uk/programs/profit/); FireDock (http:/bioinfo3d.cs.tau.ac.il/FireDock/); DoGSiteScorer (https://proteins.plus/); McLachlan algorithm

For manuscripts utilizing custom algorithms or software that are central to the research but not yet described in published literature, software must be made available to editors/reviewers upon request. We strongly encourage code deposition in a community repository (e.g. GitHub). See the Nature Research guidelines for submitting code & software for further information.

Data

Policy information about availability of data

All manuscripts must include a data availability statement. This statement should provide the following information, where applicable:

- Accession codes, unique identifiers, or web links for publicly available datasets
- A list of figures that have associated raw data
- A description of any restrictions on data availability

Crystal structures for calcineurin complexes have been deposited in Protein Data Bank (https://clicktime.symantec.com/3Lbn1725GvPQimrdCJSmn7w6H2?u=https%3A%2F%2Fdeposit.wwpdb.org%2Fdeposition%2F) PDB DEP ID: D_1000231074. The structures are on hold until August 10, 2020. The PDB ID numbers for the structures are 5B8I (Coccidiodes immitis, already released), 6TZ6 (Candida albicans), 6TZ7 (Aspergillus fumigatus), 6TZ8 (Cryptococcus neoformans). The details on crystallization and the PDB validation reports have been submitted as supporting data.
The following PDB IDs can be accessed using these weblinks: 5B8I [https://www.rcsb.org/structure/5b8i]; 6TZ6 [https://www.rcsb.org/structure/6tz6; 6TZ7 [https://www.rcsb.org/structure/6tz7; 6TZ8 [https://www.rcsb.org/structure/6tz8].

Field-specific reporting

Please select the best fit for your research. If you are not sure, read the appropriate sections before making your selection.

☒ Life sciences ☐ Behavioural & social sciences ☐ Ecological, evolutionary & environmental sciences

For a reference copy of the document with all sections, see nature.com/authors/policies/ReportingSummary-flat.pdf

Life sciences study design

All studies must disclose on these points even when the disclosure is negative.

Sample size	For genetic manipulation and mutation experiments in Aspergillus fumigatus, threee strains were individually confirmed for each mutation by PCR and DNA sequencing. Three strains were processed for growth experiments in triplicate to confirm the effect of the mutations. In vitro antifungal susceptibility testing assays in the various fungal pathogens (A. fumigatus Af293; A. fumigatus CEA10; C. neoformans WT (H99), M. circinelloides f. lusitanicus, M. circinelloides f. circinelloides, and C. albicans) were performed in triplicate.
Data exclusions	No data were excluded from the analyses in this study.
Replication	All fungal growth and animal infection experiments were replicated to confirm the results and their reproducibility.
Randomization	For animal infection experiments the mice were grouped randomly.
Blinding	For animal infection experiments the investigators were blinded to different groups of mice for data collection and analysis.

Reporting for specific materials, systems and methods

Materials & experimental systems

n/a	Involved in the study	
☐	☒	Unique biological materials
☐	☒	Antibodies
☒	☐	Eukaryotic cell lines
☒	☐	Palaeontology
☐	☒	Animals and other organisms
☒	☐	Human research participants

Methods

n/a	Involved in the study	
☒	☐	ChIP-seq
☐	☒	Flow cytometry
☒	☐	MRI-based neuroimaging

Unique biological materials

Policy information about availability of materials

Obtaining unique materials	All materials used for the study are available from the corresponding authors and other commercial sources as specified in Materials and Methods.

Antibodies

Antibodies used

Anti-GFP Rabbit polyclonal antibody (GenScript; A01704); Anti-Rabbit anti-IgG Peroxidase-labeled (Rockland; 611-1302); Anti-CD4 antibody (eBioscience; 14-9766-80); Anti-IL-2 Monoclonal Antibody (eBioscience; JES6-5H4); IFN gamma Monoclonal Antibody (eBioscience XMG1.2).

Validation

GenScript Rabbit Anti-GFP Polyclonal Antibody (GenScript; A01704) is developed in rabbit using purified recombinant full-length GFP protein. This polyclonal antibody is highly purified from rabbit antiserum by immunoaffinity chromatography.GenScript Rabbit Anti-GFP Polyclonal Antibody reacts with GFP fusion proteins. The antibody also reacts with other variants of GFP, such as CFP, YFP, eGFP and GFPuv.The following concentration ranges are recommended starting points for this product. Western blot: 0.5-1 µg/ml.

Anti-CD4 antibody (eBioscience; 14-9766-80): This 4SM95 monoclonal antibody reacts with the mouse CD4 molecule, a 55 kDa cell surface receptor expressed by a majority of thymocytes, a subpopulation of mature T cells, and dendritic cells. This 4SM95 antibody has been reported for use in western blotting, microscopy, and immunohistochemical staining of formalin-fixed paraffin embedded tissue sections. 14-9766 was used in Flow cytometry/Cell sorting to identify a inducible barrier to CNS entry at the glia limitans, which may be therapeutically targetable in inflammatory CNS disease.

Anti-IL-2 (eBioscience; JES6-5H4): The JES6-5H4 monoclonal antibody reacts with mouse interleukin-2 (IL-2), a 17 kDa T cell growth factor and a major immunoregulatory cytokine.T he purified JES6-5H4 has been reported for use in blocking of fluorochrome conjugated JES6-5H4 in intracellular flow cytometry experiments to serve as specificity control. JES6-5H4 is reported for detection of mouse IL-2 by ELISA, immunoprecipitation, and for neutralizing IL-2 binding and biological activity.

IFN gamma Monoclonal Antibody (eBioscience XMG1.2): The XMG1.2 antibody reacts with mouse interferon (IFN) gamma. The XMG1.2 antibody is a neutralizing antibody. The XMG1.2 antibody has been reported for use in ELISA, intracellular staining for flow cytometric analysis, immunoblotting (WB), and for neutralization of IFN gamma bioactivity.

Animals and other organisms

Policy information about studies involving animals; ARRIVE guidelines recommended for reporting animal research

Laboratory animals

For Crytococcal murine infection assays, groups of 10 female A/J mice (15-20 g) each were infected with C. neoformans H99 and 3 animals from each group were randomly selected for euthanasia and organs harvested for fungal burden analysis.

For Aspergillus murine infection model groups of 20 mice each (Six-week-old CD1 male mice; mean weight 22.5 g) were infected with the A. fumigatus.

For the murine model of invasive mucor mycosis groups of 5 male BALB/c mice (20g) each were infected.

For the murine model of invasive candidiasis, groups of 6 male CD1 mice (20-25 g) each were infected.

For testing the in vivo immunosuppressive activity of FK506 and APX879, groups of 5 female healthy C57BL/6 mice were treated daily for 8 days with vehicle, 5 mg/kg FK506, or 20 mg/kg APX879 via IP injection. 24 hours following initial treatment, animals were immunized with the antigen NP-OVA via subcutaneous instillation. Drug treatment continued daily for 7 days following immunization to allow for T cell-dependent Germinal Center (GC) B cell response.

Wild animals

This study did not involve wild animals.

Field-collected samples

This study did not involve samples collected from the field.

Flow Cytometry

Plots

Confirm that:

☒ The axis labels state the marker and fluorochrome used (e.g. CD4-FITC).

☒ The axis scales are clearly visible. Include numbers along axes only for bottom left plot of group (a 'group' is an analysis of identical markers).

☒ All plots are contour plots with outliers or pseudocolor plots.

☒ A numerical value for number of cells or percentage (with statistics) is provided.

Methodology

Sample preparation

Pooled spleen and lymph nodes from C57BL/6 mice were homogenized through a 40 µm filter and subjected to magnetic enrichment of naive CD4+ T cells using the eBioscience MagniSort mouse naive T cell kit (eBioscience/Invitrogen/ThermoFisher). Cells were grown in Iscove's Modified Dulbecco's Medium (IMDM) supplemented with glutamine, penicillin, streptomycin, gentamicin, 2-mercaptoethanol, and 10% FBS. Purified naive T cells were cultured on anti-hamster IgG coated plates in the presence of hamster anti-CD3 epsilon and anti-CD28 antibodies (eBioscience), neutralizing anti-IL-4 antibody (eBioscience), recombinant IL-12 (10 ng/mL), and recombinant IL-2 (50 U/mL) for 72 hours. Cells were cultured in the presence of serially diluted FK506 or APX879 suspended in DMSO during these 72 hours. During the last 4 hours of culture, phorbol 12-myristate 13-

acetate (PMA; Sigma), ionomycin (Sigma), and GolgiStop (BD Biosciences) were added to the culture to facilitate detection of intracellular cytokines.

For in vivo immunosuppression comparison experiment between FK506 and APX879, groups of 5 female healthy C57BL/6 mice were treated daily for 8 days with vehicle, 5 mg/kg FK506, or 20 mg/kg APX879 via IP injection. 24 hours following initial treatment, animals were immunized with the antigen NP-OVA via subcutaneous instillation. Drug treatment continued daily for 7 days following immunization to allow for T cell-dependent Germinal Center (GC) B cell response. Animal lymph nodes were harvested 7 days following immunization and sorted to isolate populations of T helper cells and GC B cells. Abundance of T helper cells and GC B cells from each animal was determined using FlowJo software.

Instrument

BD LSRFortessa X-20 flow cytometer

Software

FlowJo v9.8

Cell population abundance

Abundance measurements are provided as % IL-2+ of Live CD4+ T cells identified using the gating strategy below. Gates for IL-2+ cells were determined based on A) bimodality of the untreated (DMSO) sample and B) absence of signal in the high-concentration treated samples.

Gating strategy

All plots were gated on FSC-A x SSC-A to exclude debris -> FSC-A x FSC-H to include only singlets -> CD4+ Live/Dead Dye -

☒ Tick this box to confirm that a figure exemplifying the gating strategy is provided in the Supplementary Information.
