# Supplementary material for: Harnessing calcineurin-FK506-FKBP12 crystal structures from invasive fungal pathogens to develop antifungal agents
Source: Nat Commun. 2019 Sep 19;10:4275. doi: 10.1038/s41467-019-12199-1 (PMC6753081; doi:10.1038/s41467-019-12199-1)
Supplement: Supplementary file 8 — Source Data [file 41467_2019_12199_MOESM8_ESM.zip › SOURCE-DATA-NCOMMS-18-31711B-2019/KEY-RESOURCE-TABLE.docx]

**KEY RESOURCE TABLE**

| REAGENT or RESOURCE | SOURCE | IDENTIFIER |
| --- | --- | --- |
| Vectors for Protein Expression |  |  |
| pBacgus4X-1 | Novagen | Cat#70045 |
| pBacgus-1 | Novagen | Cat#70054 |
| BestBac 2.0 | Expression Systems | Cat#91-002 |
| pET-15b | Novagen | Cat#69661 |
| pUCGH-Fkbp12promo-Fkbp12-Fkbp12 term | This study | N/A |
| pUCGH-CnaApromo-CnaA-CnaA term | This study | N/A |
| Protein Purification |  |  |
| Tni Cells | Expression Systems | Cat#94-002F |
| ESF 921™ Insect Cell Culture Medium | Expression Systems | Cat#96-001-01 |
| Terrific Broth | Teknova | Cat#T7060-06 |
| IPTG | GoldBio | Cat#I2481 |
| Ni^2+^-HiTrap Chelating HP column | GE Healthcare | Cat#17040801 |
| HiTrap Benzamidine FF | GE Healthcare | Cat#17514401 |
| Vivaspin 20 PES 10kDa MWCO | Sartorius | Cat#VS2001 |
| VivaSpin 15 PES 5kDa MWCO | Sartorius | Cat#VS15T11 |
| VivaSpin Turbo 15 PES 10kDa MWCO | Sartorius | Cat#VS15T01 |
| Superdex Hi Load 200 16/600 | GE Healthcare | Cat#28989335 |
| Superdex 200 10/300 GL | GE Healthcare | Cat#17517501 |
| Sephacryl S100HR XK26/60 FPLC column | GE Healthcare | Cat#17-1194-01 |
| Thrombin | BioPharm | Cat#91-035 |
| Antibiotics and Antibodies |  |  |
| Kanamycin | Teknova | Cat#K2125 |
| Carbenicillin disodium salt | Genesee Scientific | Cat#25-532 |
| Ampicillin sodium salt | G Biosciences | Cat#A051-B |
| Chloramphenicol | Fisher | Cat#BP904-100 |
| Amphotericin B (Ambisome) | Astellas Pharma | Cat#1397-89-3 |
| Hygromycin B | ThermoScientific | Cat#10687010 |
| The^TM^ anti-GFP Rabbit polyclonal antibody | GenScript | Cat#A01704 |
| Anti-Rabbit anti-IgG Peroxidase-labeled | Rockland | Cat#611-1302 |
| Anti-CD4 antibody | eBioscience | Cat#14-9766-80 |
| anti-IL-2 | eBioscience | Cat#JES6-5H4 |
| Chemicals and Drugs |  |  |
| FK506 | Astellas Pharma | Cat#109581-93-3 |
| APX879 | Amplyx | N/A |
| Fluconazole | Sagent Pharmaceuticals NDC | Cat#25021-113-82 |
| Fixable Viability Dye eFluor 780 | eBioscience | Cat#65-0865-14 |
| Experimental Models: Strains |  |  |
| *Aspergillus fumigatus* Af293 | Fungal Genetics Stock Center | FGSC A1100 |
| *Aspergillus fumigatus* CEA10 | Fungal Genetics Stock Center | FGSC A1163 |
| *Candida albicans* SC5314 | American Type Culture Collection | ATCC MYA-2876 |
| *Cryptococcus neoformans* H99 | American Type Culture Collection | ATCC 208821 |
| *M. circinelloides f. lusitanicus* | American Type Culture Collection | ATCC 1216B |
| *M. circinelloides f. circinelloides* | American Type Culture Collection | ATCC 20132 |
| Software and Algorithms |  |  |
| GeneComposer | Emerald Bio | N/A |
| GeneArt | ThermoFisher | N/A |
| Phaser | McCoy et al., 2007 | N/A |
| Molrep | Vagin and Teplyakov, 2010 | N/A |
| Phenix | Adams et al., 2010 | N/A |
| COOT | Emsley et al., 2010 | N/A |
| Molprobity | http://molprobity.biochem.duke.edu/ | N/A |
| GraphPad Prism 7 | https://www.graphpad.com/ | N/A |
| NMRPipe | https://www.ibbr.umd.edu/nmrpipe/ | N/A |
| NMRViewJ | https://www.nmrbox.org/registry/nmrviewj | N/A |
| PINE | http://i-pine.nmrfam.wisc.edu/ | N/A |
| AutoAssign | http://nmr.cabm.rutgers.edu/autoassign/cgi-bin/aaenmr.py | N/A |
| qtPISA | Krissinel, 2015 | N/A |
| MODELLER v9.18 | https://salilab.org/modeller/ | N/A |
| PyMOL Molecular Graphics System | Schrödinger, LLC | N/A |
| HADDOCK | http://www.bonvinlab.org/software/haddock2.2/ | N/A |
| McLachlan algorithm | McLachlan, 1982 | N/A |
| ProFit | http://www.bioinf.org.uk/programs/profit/ | N/A |
| GROMACS 5.0.1 | http://www.gromacs.org/ | N/A |
| FireDock | http://bioinfo3d.cs.tau.ac.il/FireDock/ | N/A |
| DoGSiteScorer | https://proteins.plus/ | N/A |
| Deposited Data for Crystal Structures |  |  |
| *Aspergillus fumigatus* | Canadian Light Source Beamline 08-ID | 6TZ7 |
| *Candida albicans* | Advanced Photon Source Beamline 21-ID-G (LS-CAT) | 6TZ6 |
| *Cryptococcus neoformans* | Advanced Photon Source Beamline 21-ID-G (LS-CAT) | 6TZ8 |
| *Coccidioides immitis* | Advanced Photon Source Beamline 21-ID-G (LS-CAT) | 5B8I |

N/A-Not Applicable
