# Supplementary material for: Harnessing calcineurin-FK506-FKBP12 crystal structures from invasive fungal pathogens to develop antifungal agents
Source: Nat Commun. 2019 Sep 19;10:4275. doi: 10.1038/s41467-019-12199-1 (PMC6753081; doi:10.1038/s41467-019-12199-1)
Supplement: Supplementary file 8 — Source Data [file 41467_2019_12199_MOESM8_ESM.zip › SOURCE-DATA-NCOMMS-18-31711B-2019/SOURCE-DATA-Juvvadi-etal-Calcineurin-FKBP12-Structure-Manuscript.pptx]

## Slide 1
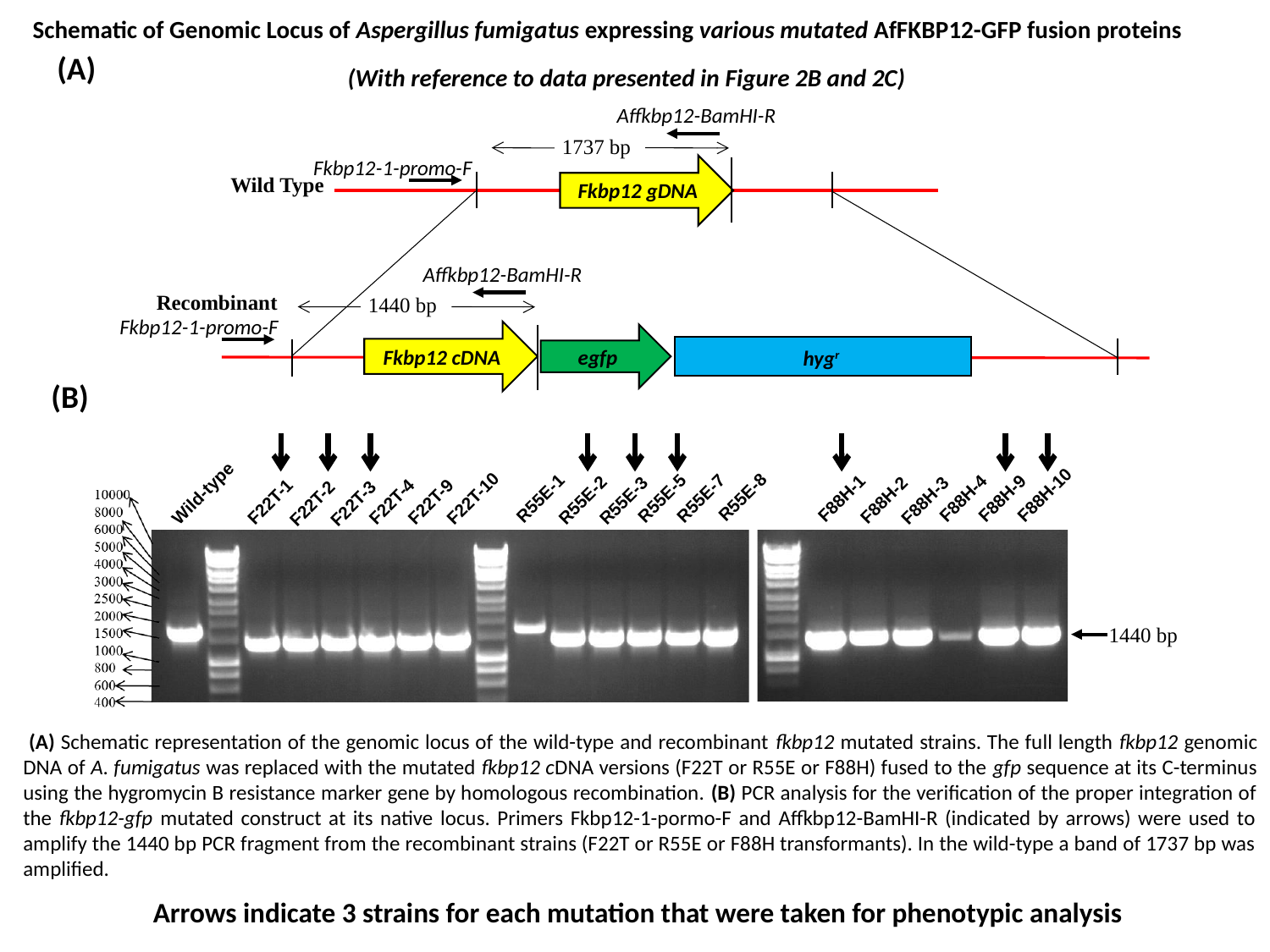

Schematic of Genomic Locus of Aspergillus fumigatus expressing various mutated AfFKBP12-GFP fusion proteins
(A)
(With reference to data presented in Figure 2B and 2C)
Affkbp12-BamHI-R
 1737 bp
Fkbp12-1-promo-F
Fkbp12 gDNA
Wild Type
Affkbp12-BamHI-R
Recombinant
 1440 bp
Fkbp12-1-promo-F
Fkbp12 cDNA
egfp
hygr
(B)
Wild-type
F88H-10
F88H-4
F88H-1
F88H-2
F88H-9
F88H-3
R55E-8
R55E-5
R55E-1
R55E-2
R55E-7
R55E-3
F22T-10
F22T-4
F22T-1
F22T-2
F22T-9
F22T-3
 1440 bp
 (A) Schematic representation of the genomic locus of the wild-type and recombinant fkbp12 mutated strains. The full length fkbp12 genomic DNA of A. fumigatus was replaced with the mutated fkbp12 cDNA versions (F22T or R55E or F88H) fused to the gfp sequence at its C-terminus using the hygromycin B resistance marker gene by homologous recombination. (B) PCR analysis for the verification of the proper integration of the fkbp12-gfp mutated construct at its native locus. Primers Fkbp12-1-pormo-F and Affkbp12-BamHI-R (indicated by arrows) were used to amplify the 1440 bp PCR fragment from the recombinant strains (F22T or R55E or F88H transformants). In the wild-type a band of 1737 bp was amplified.
Arrows indicate 3 strains for each mutation that were taken for phenotypic analysis

## Slide 2
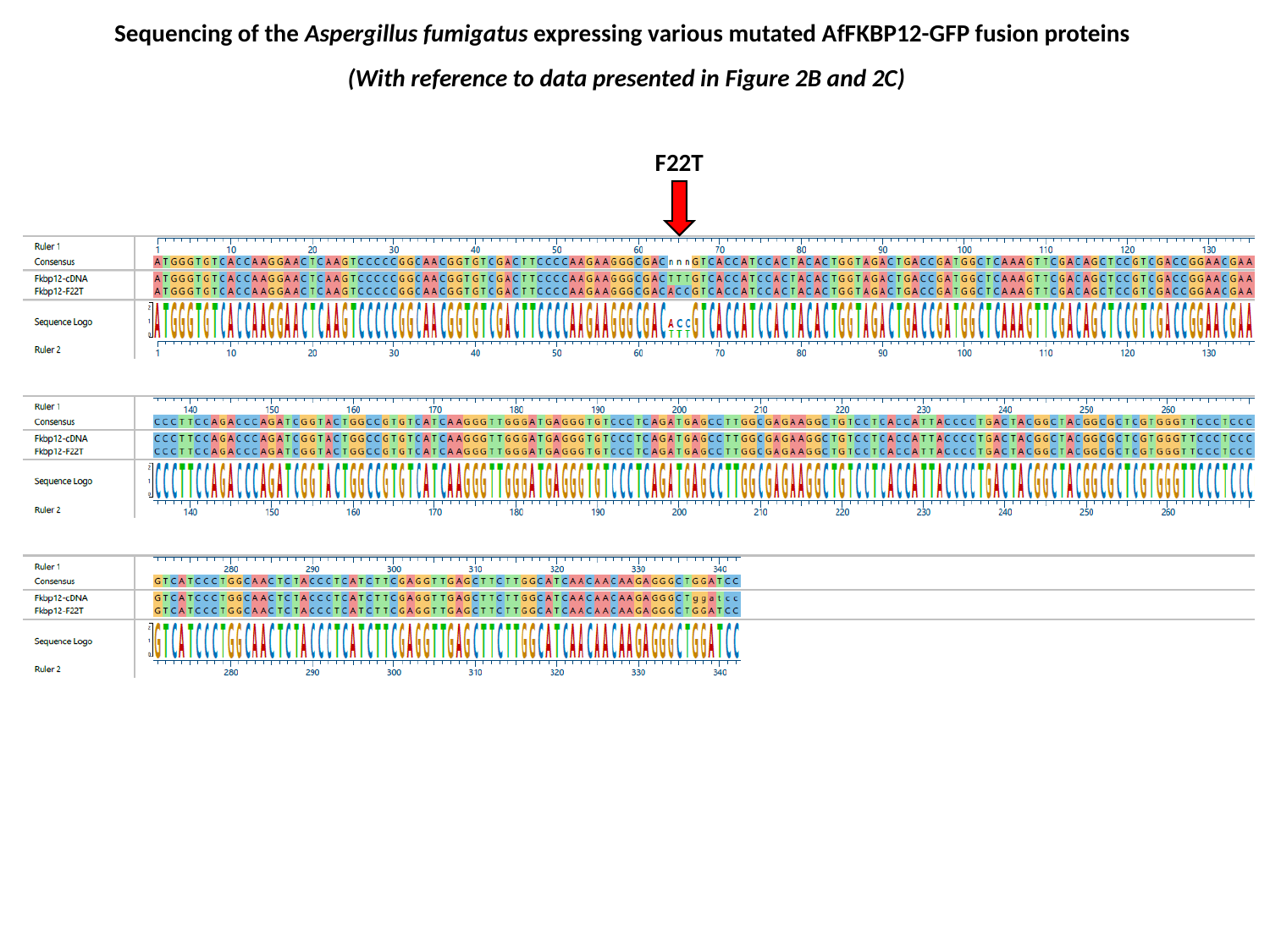

Sequencing of the Aspergillus fumigatus expressing various mutated AfFKBP12-GFP fusion proteins
(With reference to data presented in Figure 2B and 2C)
F22T

## Slide 3
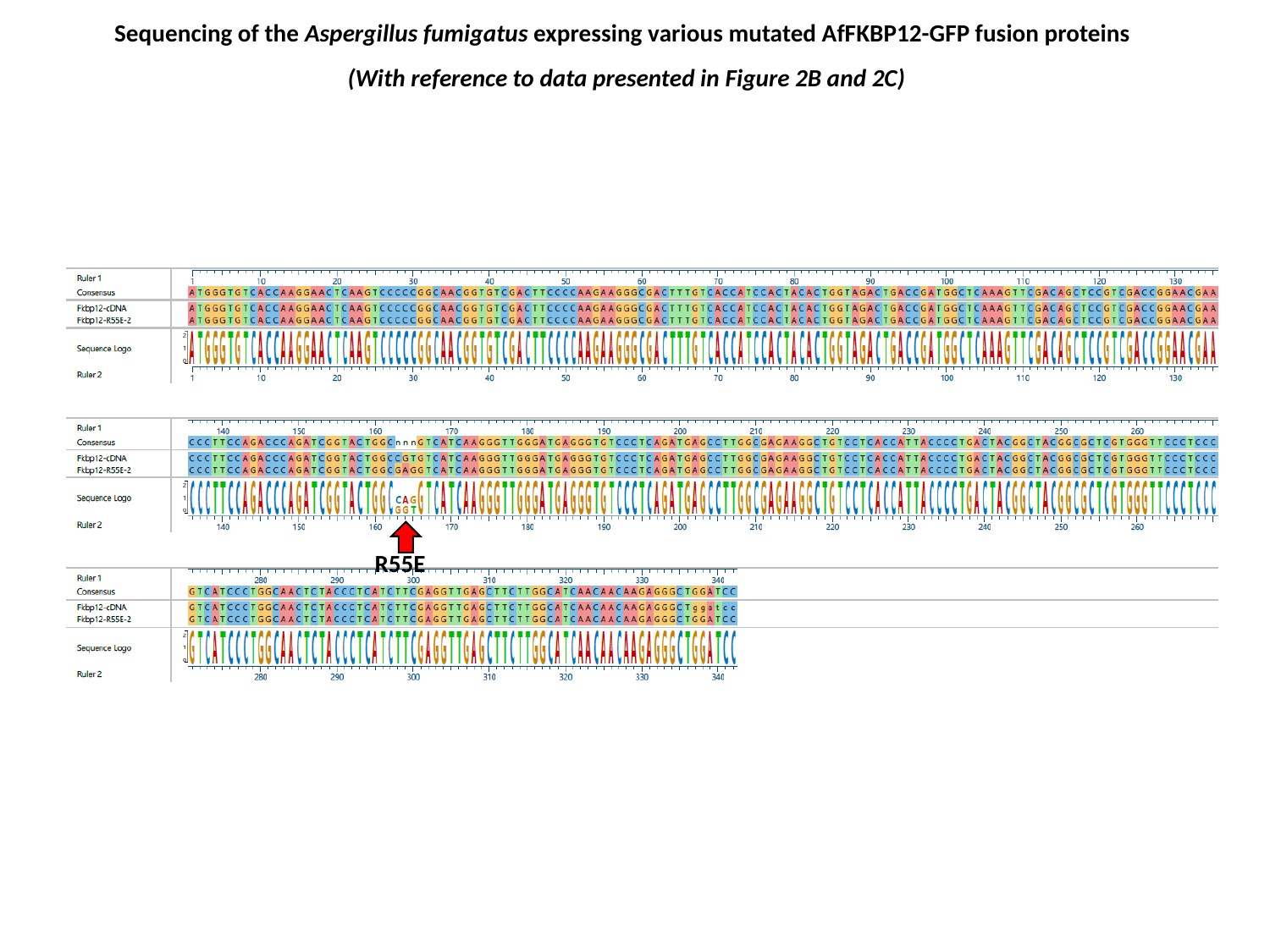

Sequencing of the Aspergillus fumigatus expressing various mutated AfFKBP12-GFP fusion proteins
(With reference to data presented in Figure 2B and 2C)
R55E

## Slide 4
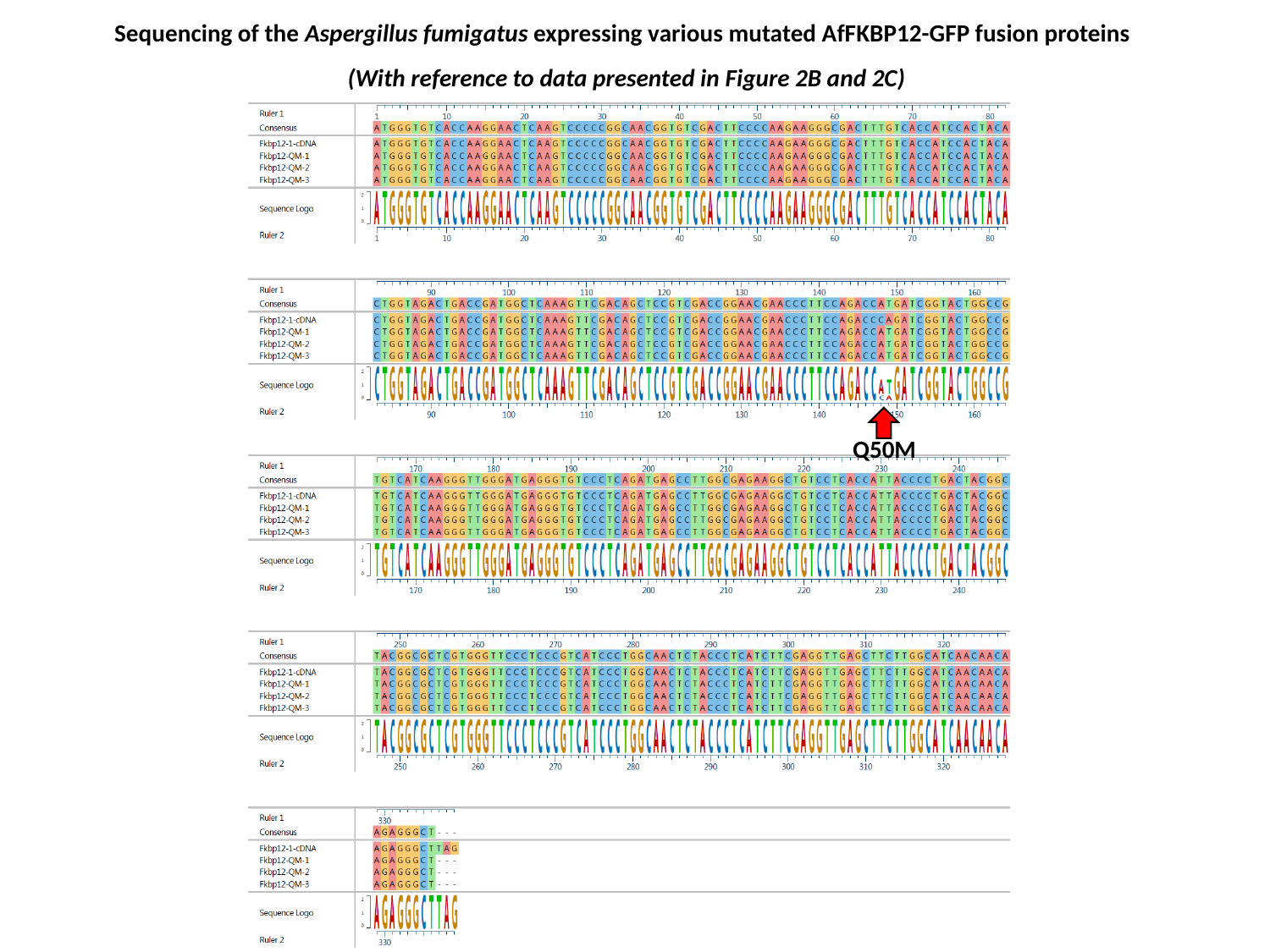

Sequencing of the Aspergillus fumigatus expressing various mutated AfFKBP12-GFP fusion proteins
(With reference to data presented in Figure 2B and 2C)
Q50M

## Slide 5
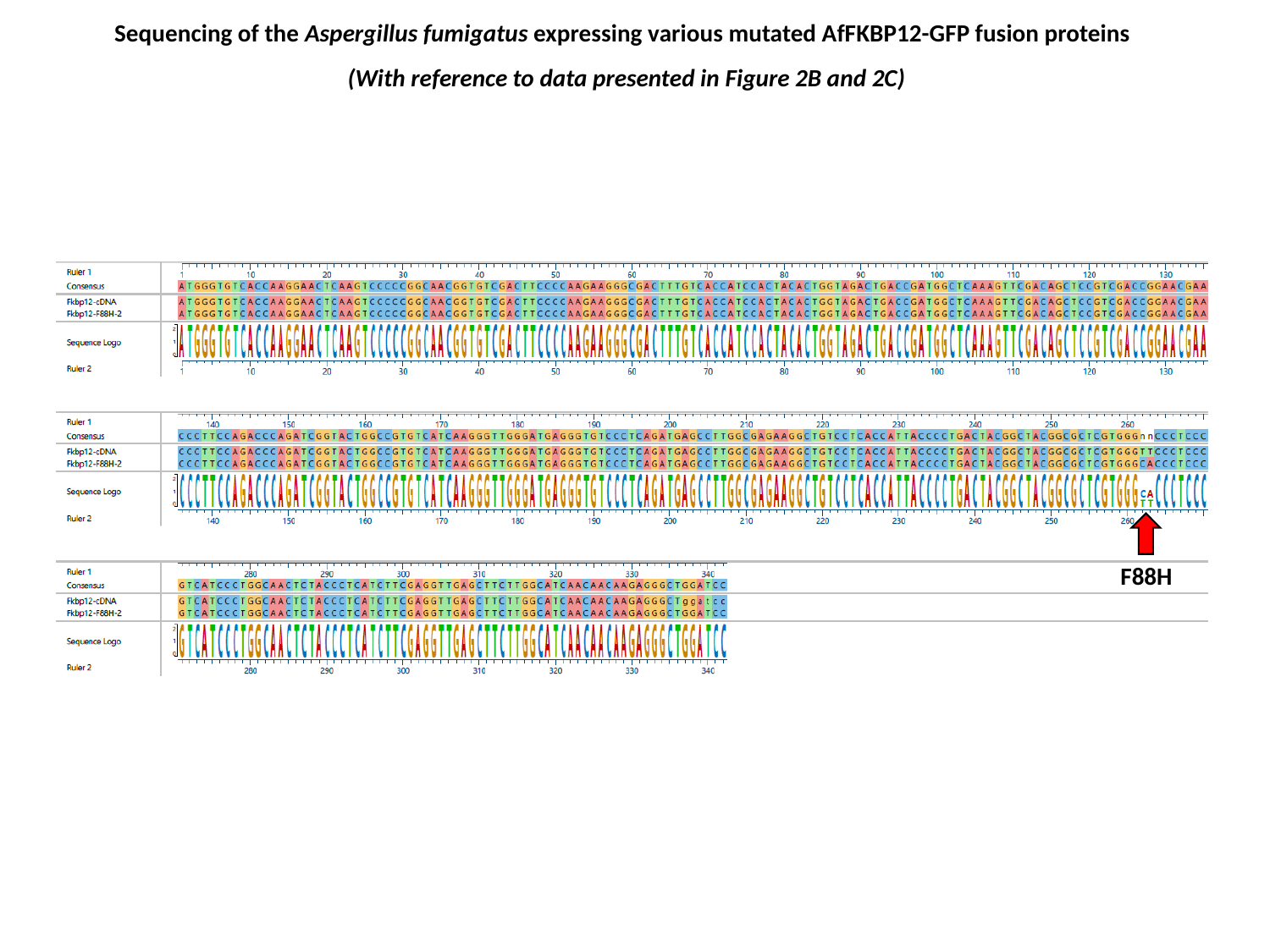

Sequencing of the Aspergillus fumigatus expressing various mutated AfFKBP12-GFP fusion proteins
(With reference to data presented in Figure 2B and 2C)
F88H

## Slide 6
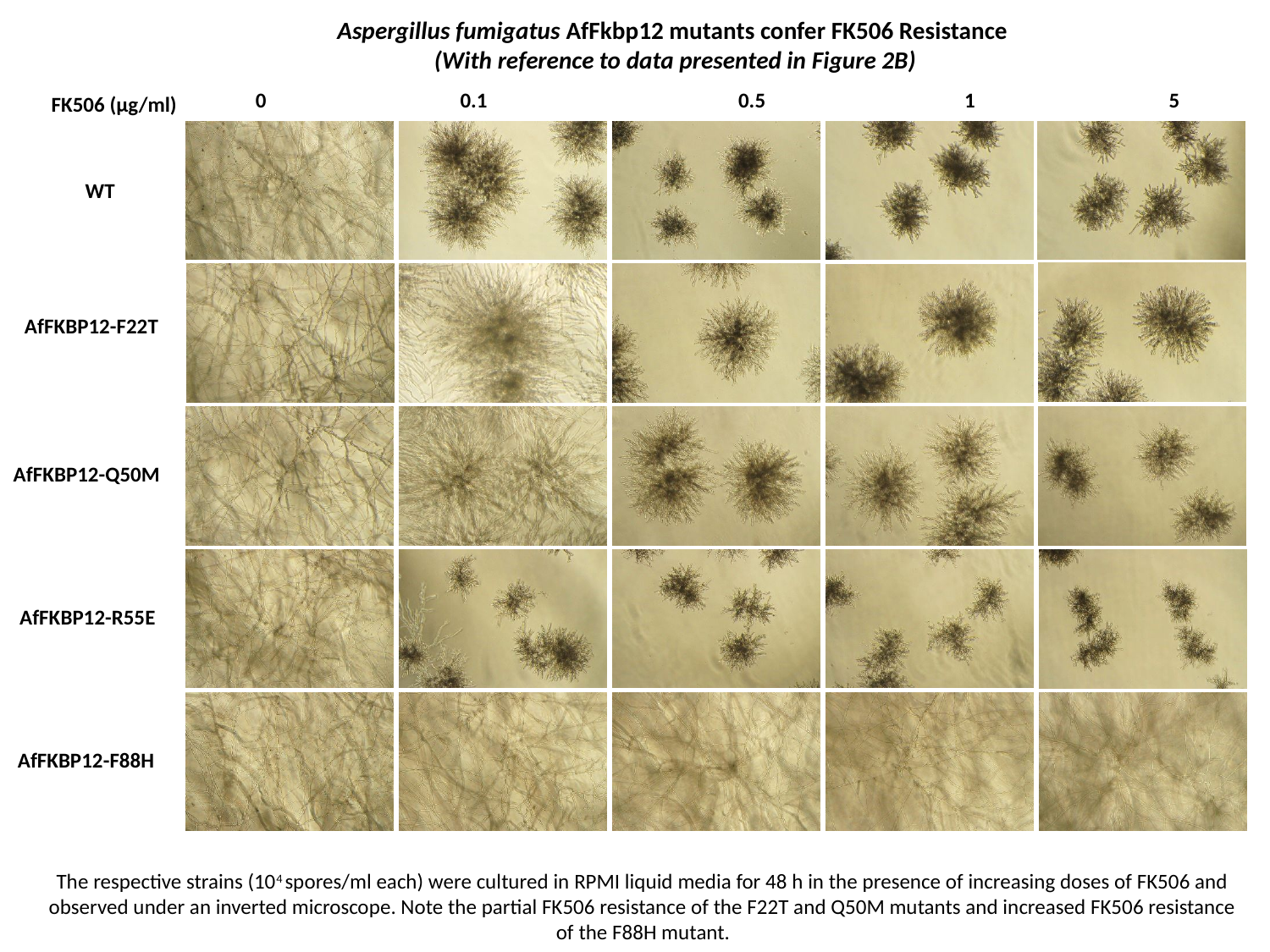

Aspergillus fumigatus AfFkbp12 mutants confer FK506 Resistance
(With reference to data presented in Figure 2B)
0 0.1		 0.5 1 5
FK506 (µg/ml)
WT
AfFKBP12-F22T
AfFKBP12-Q50M
AfFKBP12-R55E
AfFKBP12-F88H
The respective strains (104 spores/ml each) were cultured in RPMI liquid media for 48 h in the presence of increasing doses of FK506 and observed under an inverted microscope. Note the partial FK506 resistance of the F22T and Q50M mutants and increased FK506 resistance of the F88H mutant.

## Slide 7
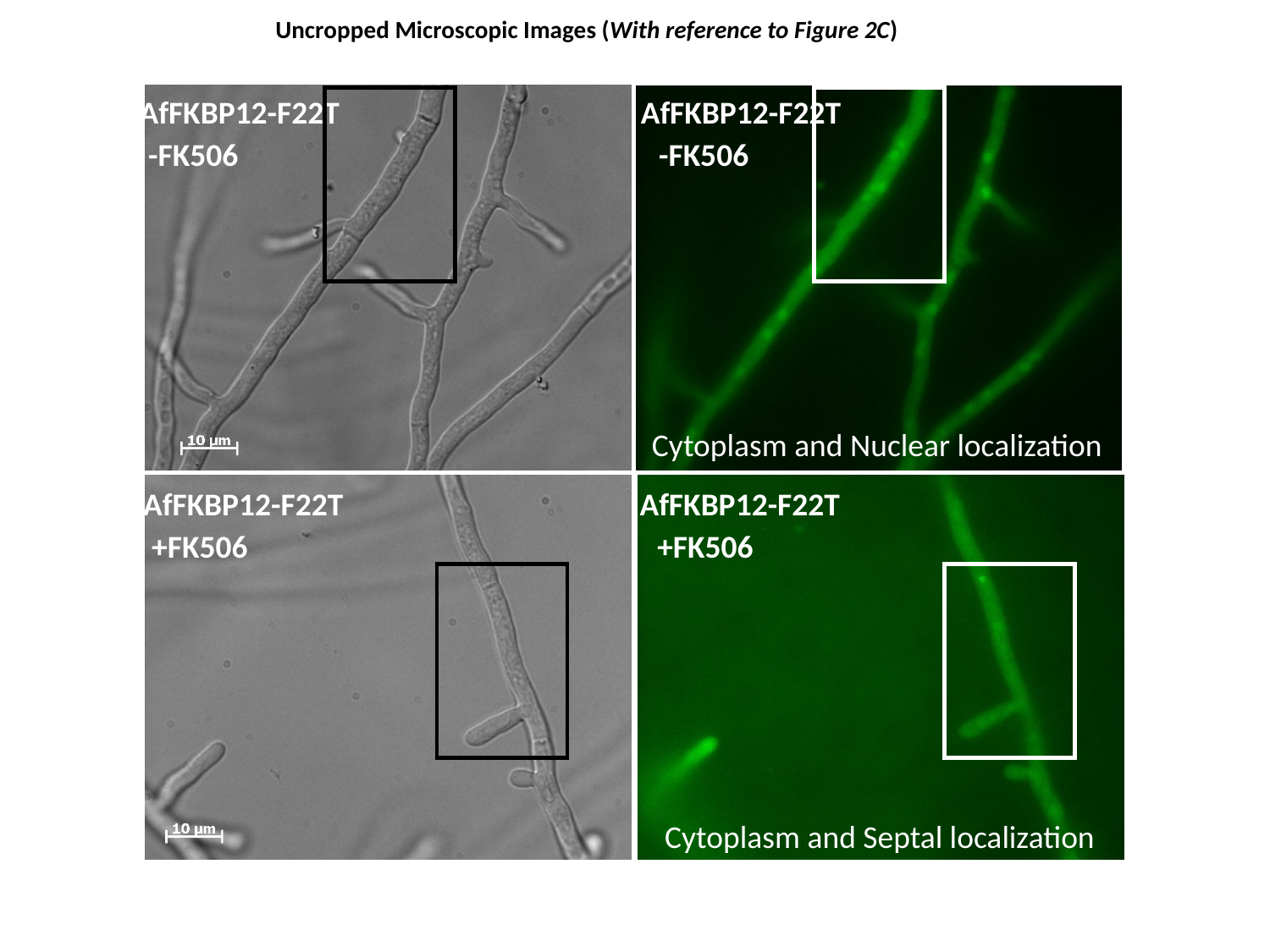

Uncropped Microscopic Images (With reference to Figure 2C)
AfFKBP12-F22T
AfFKBP12-F22T
-FK506
-FK506
AfFKBP12-F22T
AfFKBP12-F22T
+FK506
+FK506
Cytoplasm and Nuclear localization
Cytoplasm and Septal localization

## Slide 8
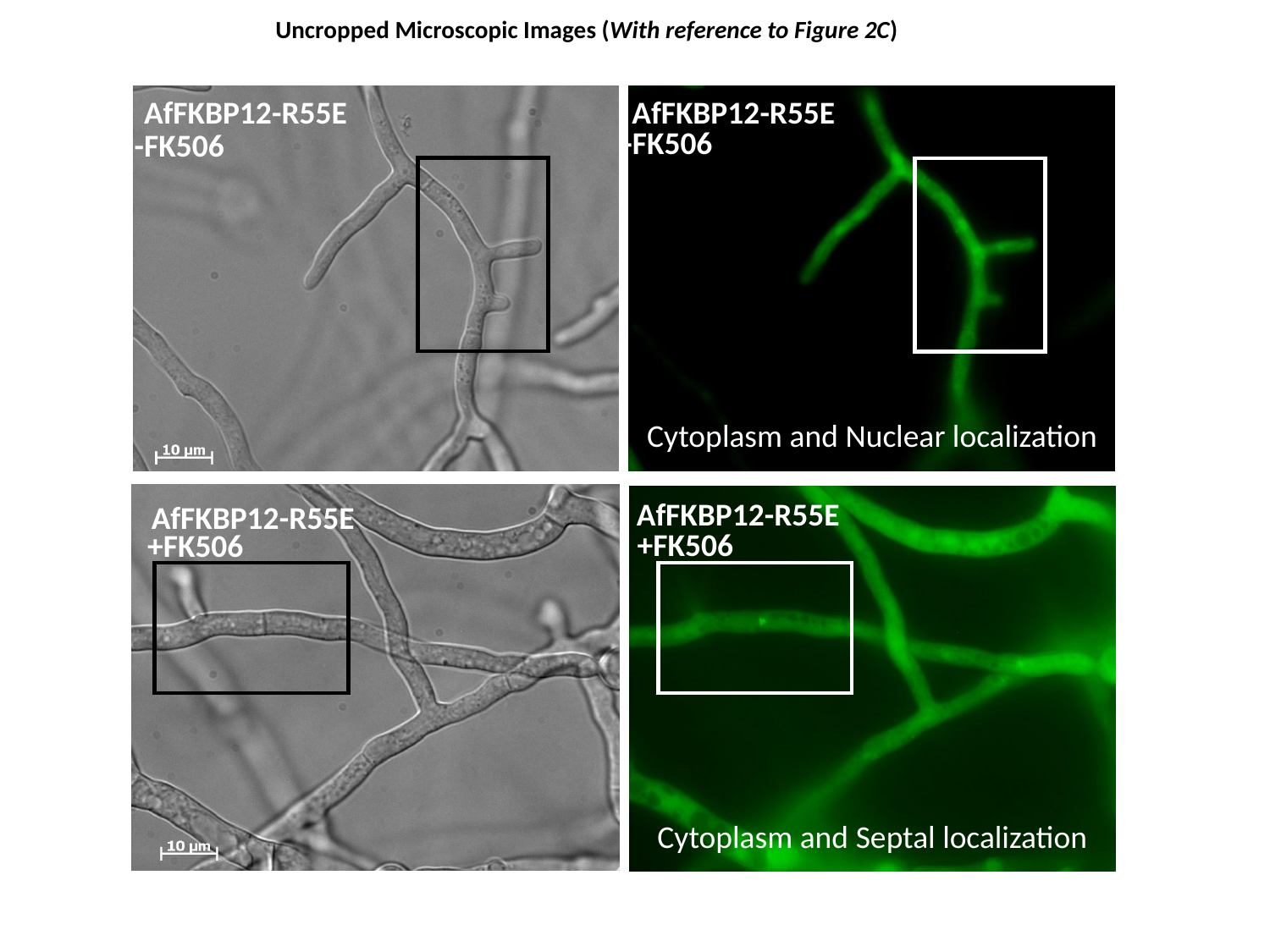

Uncropped Microscopic Images (With reference to Figure 2C)
AfFKBP12-R55E
AfFKBP12-R55E
-FK506
-FK506
AfFKBP12-R55E
AfFKBP12-R55E
+FK506
+FK506
Cytoplasm and Nuclear localization
Cytoplasm and Septal localization

## Slide 9
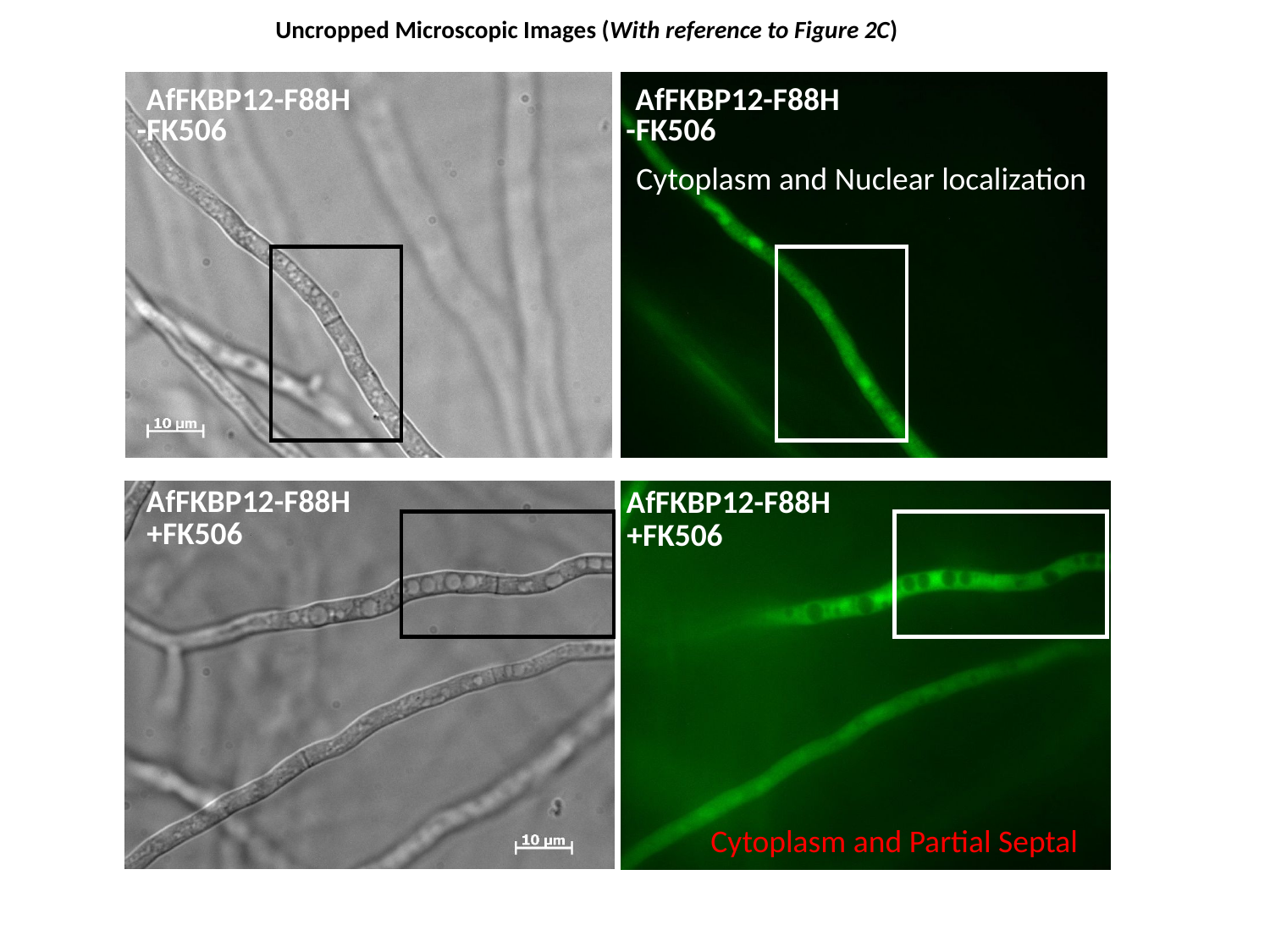

Uncropped Microscopic Images (With reference to Figure 2C)
AfFKBP12-F88H
AfFKBP12-F88H
-FK506
-FK506
AfFKBP12-F88H
AfFKBP12-F88H
+FK506
+FK506
Cytoplasm and Nuclear localization
Cytoplasm and Partial Septal

## Slide 10
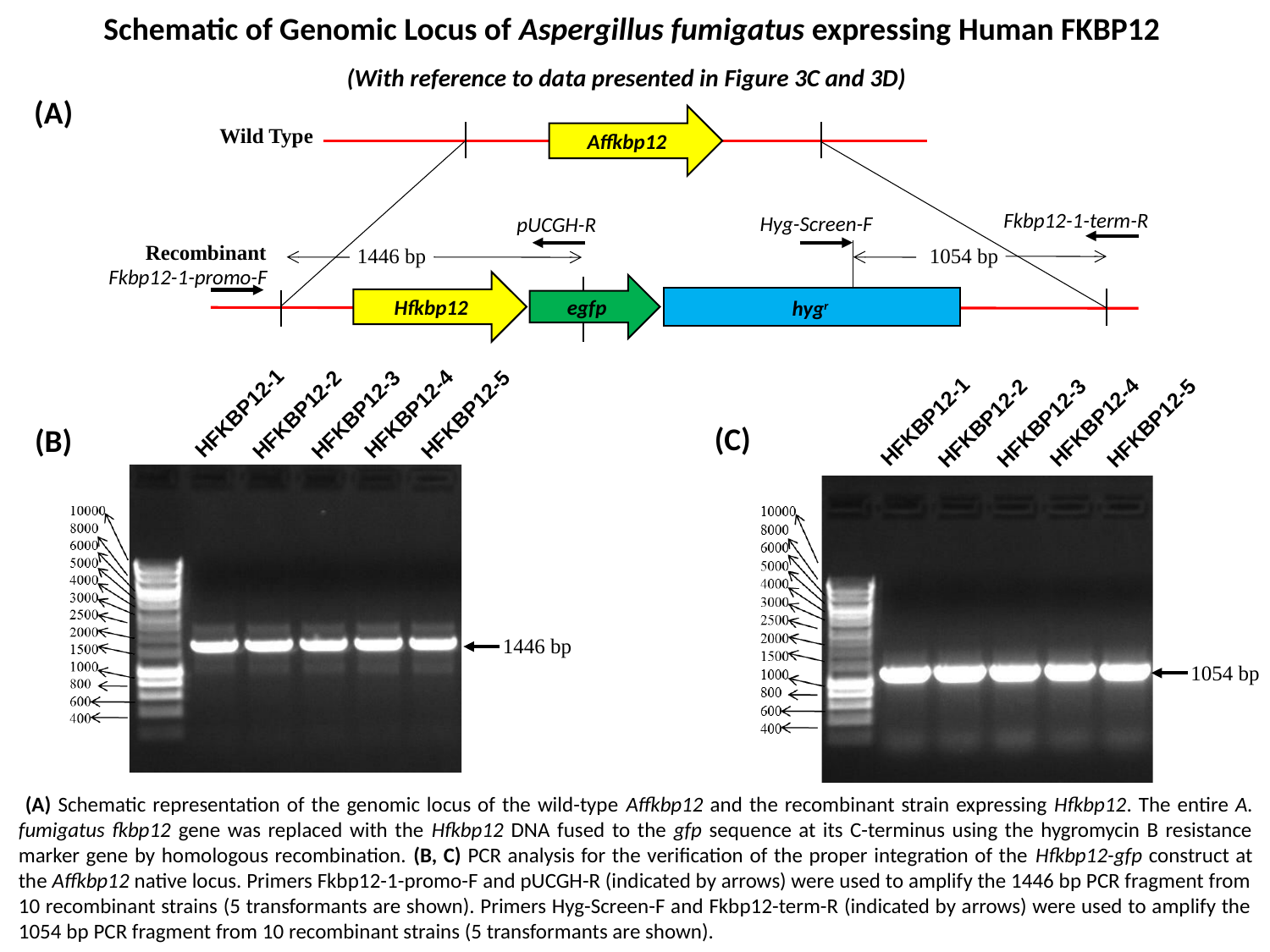

Schematic of Genomic Locus of Aspergillus fumigatus expressing Human FKBP12
(With reference to data presented in Figure 3C and 3D)
(A)
Affkbp12
Wild Type
Fkbp12-1-term-R
Hyg-Screen-F
pUCGH-R
Recombinant
 1446 bp
 1054 bp
Fkbp12-1-promo-F
Hfkbp12
egfp
hygr
HFKBP12-1
HFKBP12-4
HFKBP12-2
HFKBP12-3
HFKBP12-5
(B)
 1446 bp
HFKBP12-1
HFKBP12-4
HFKBP12-2
HFKBP12-3
HFKBP12-5
(C)
 1054 bp
 (A) Schematic representation of the genomic locus of the wild-type Affkbp12 and the recombinant strain expressing Hfkbp12. The entire A. fumigatus fkbp12 gene was replaced with the Hfkbp12 DNA fused to the gfp sequence at its C-terminus using the hygromycin B resistance marker gene by homologous recombination. (B, C) PCR analysis for the verification of the proper integration of the Hfkbp12-gfp construct at the Affkbp12 native locus. Primers Fkbp12-1-promo-F and pUCGH-R (indicated by arrows) were used to amplify the 1446 bp PCR fragment from 10 recombinant strains (5 transformants are shown). Primers Hyg-Screen-F and Fkbp12-term-R (indicated by arrows) were used to amplify the 1054 bp PCR fragment from 10 recombinant strains (5 transformants are shown).

## Slide 11
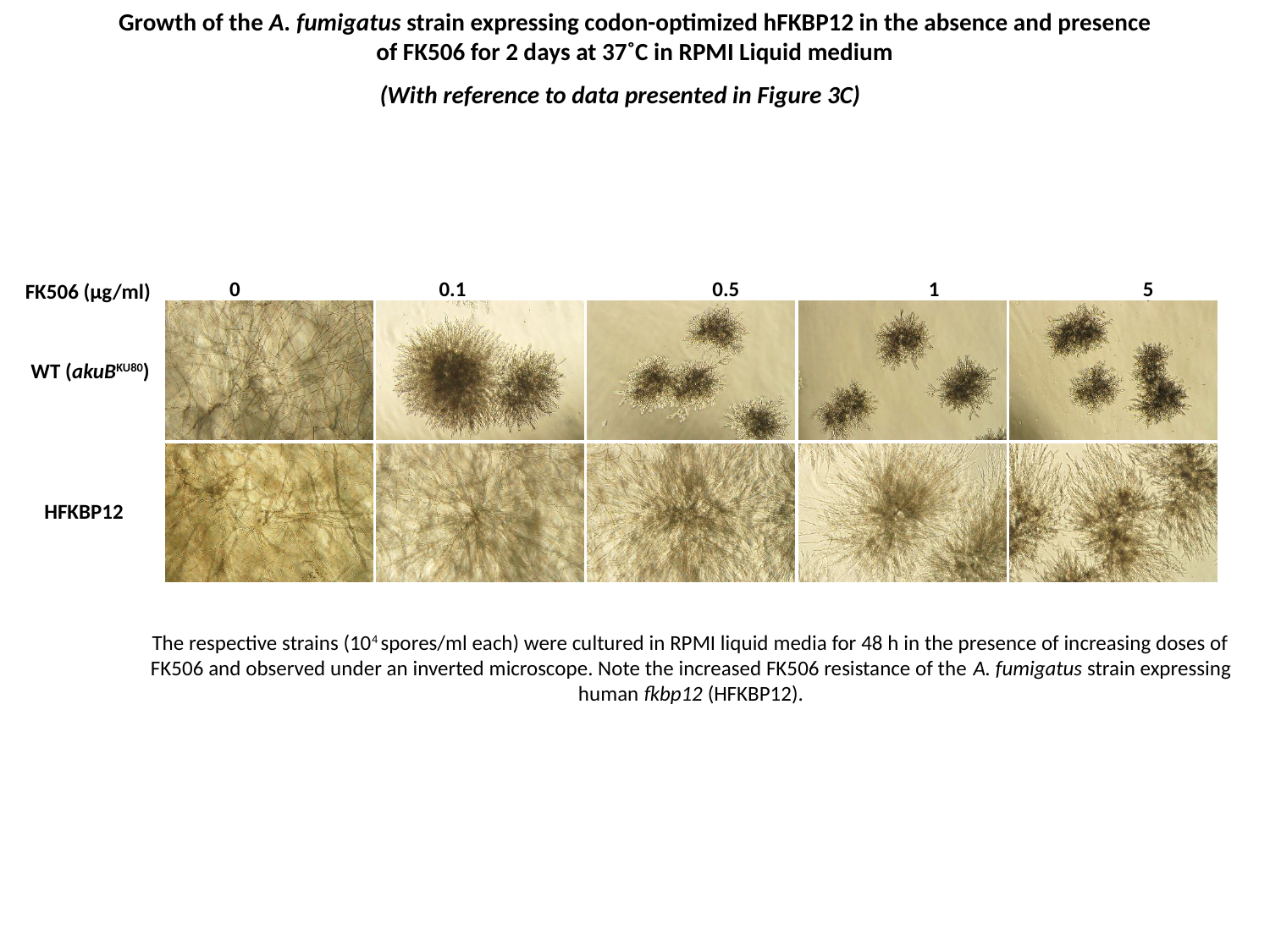

Growth of the A. fumigatus strain expressing codon-optimized hFKBP12 in the absence and presence of FK506 for 2 days at 37˚C in RPMI Liquid medium
(With reference to data presented in Figure 3C)
0 0.1		 0.5 1 5
FK506 (µg/ml)
WT (akuBKU80)
HFKBP12
The respective strains (104 spores/ml each) were cultured in RPMI liquid media for 48 h in the presence of increasing doses of FK506 and observed under an inverted microscope. Note the increased FK506 resistance of the A. fumigatus strain expressing human fkbp12 (HFKBP12).

## Slide 12
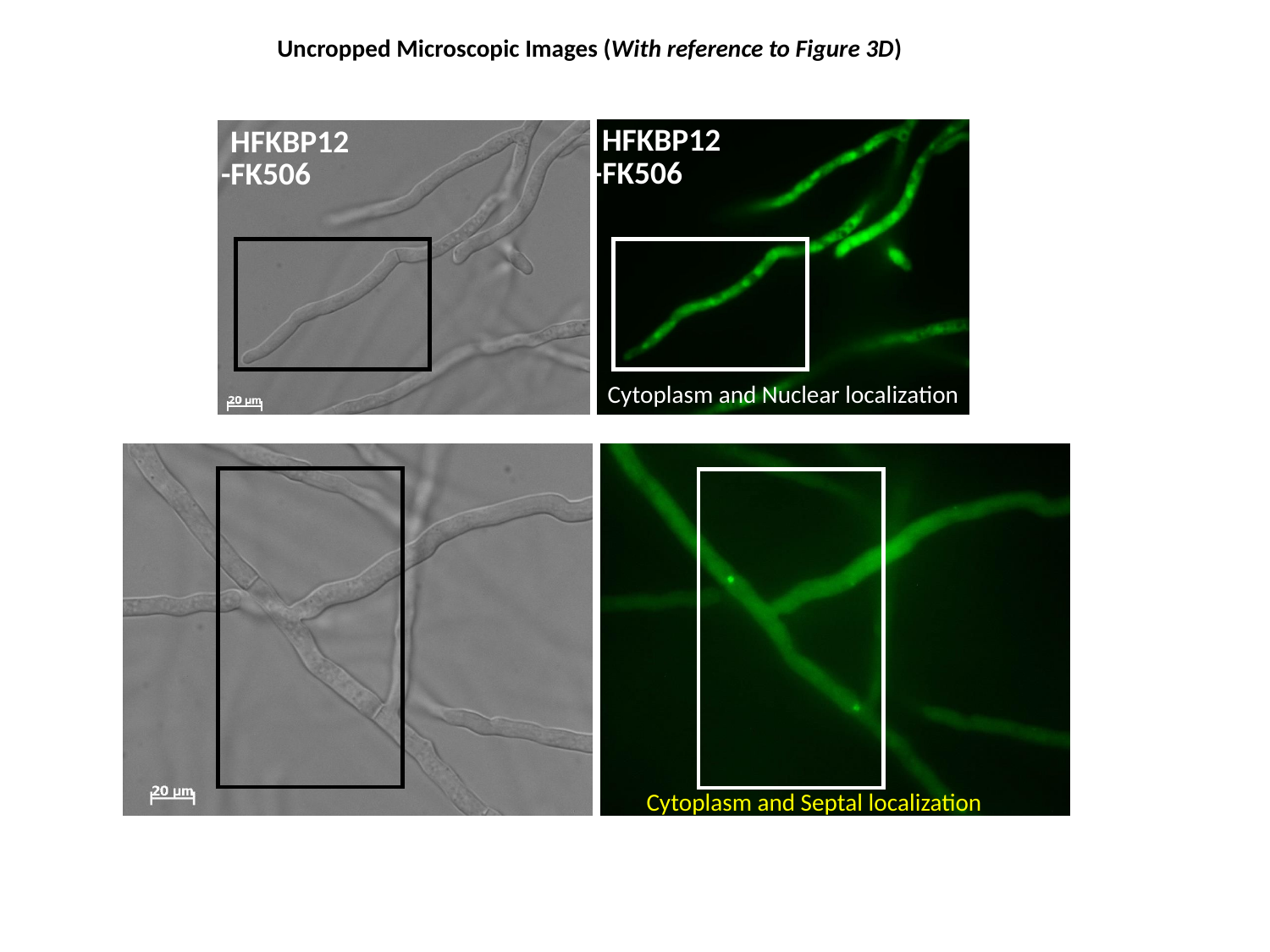

Uncropped Microscopic Images (With reference to Figure 3D)
HFKBP12
HFKBP12
-FK506
-FK506
Cytoplasm and Nuclear localization
Cytoplasm and Septal localization

## Slide 13
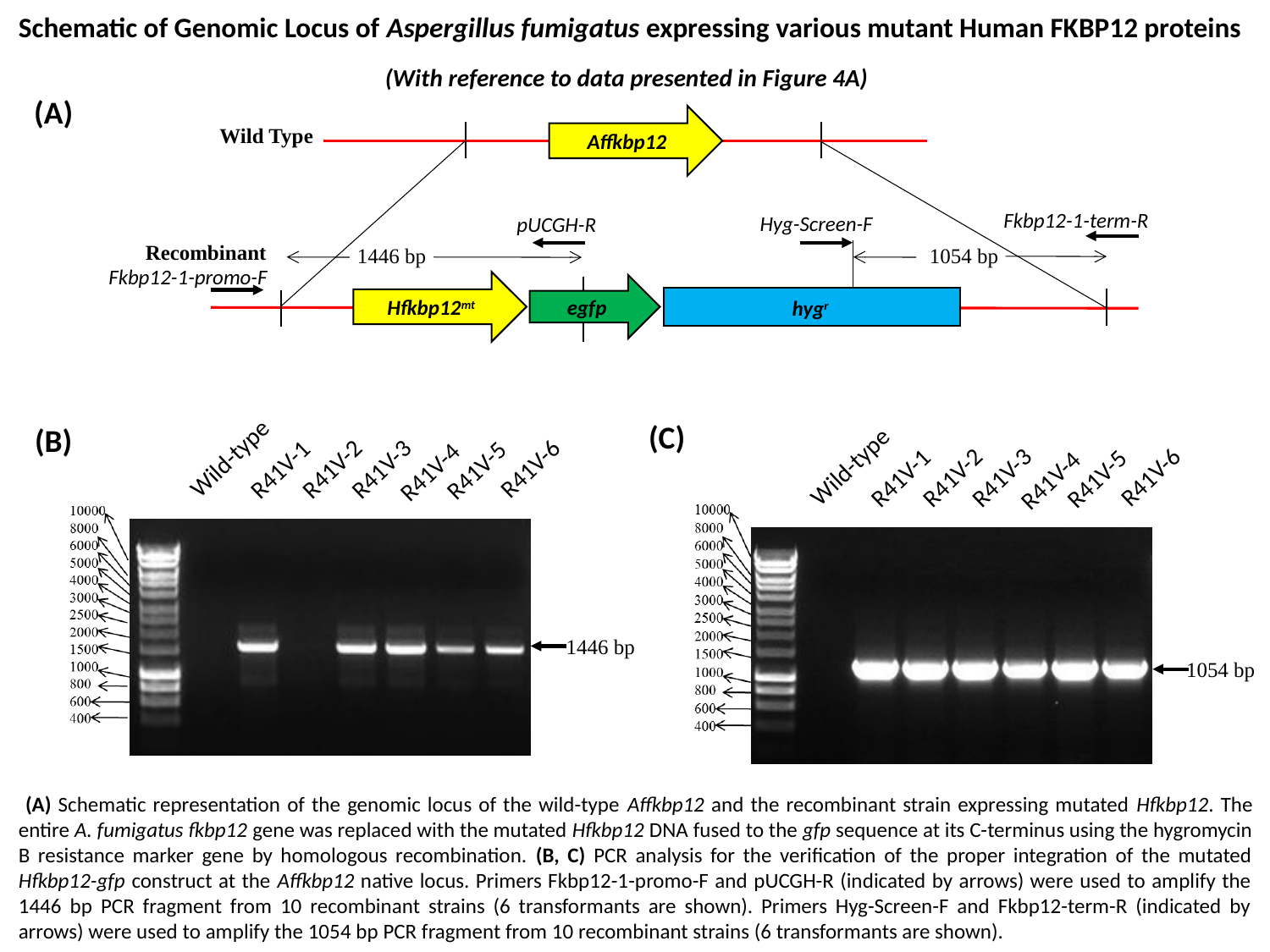

Schematic of Genomic Locus of Aspergillus fumigatus expressing various mutant Human FKBP12 proteins
(With reference to data presented in Figure 4A)
(A)
Affkbp12
Wild Type
Fkbp12-1-term-R
Hyg-Screen-F
pUCGH-R
Recombinant
 1446 bp
 1054 bp
Fkbp12-1-promo-F
Hfkbp12mt
egfp
hygr
(C)
 1054 bp
Wild-type
R41V-6
R41V-3
R41V-2
R41V-1
R41V-5
R41V-4
(B)
 1446 bp
Wild-type
R41V-6
R41V-3
R41V-2
R41V-1
R41V-5
R41V-4
 (A) Schematic representation of the genomic locus of the wild-type Affkbp12 and the recombinant strain expressing mutated Hfkbp12. The entire A. fumigatus fkbp12 gene was replaced with the mutated Hfkbp12 DNA fused to the gfp sequence at its C-terminus using the hygromycin B resistance marker gene by homologous recombination. (B, C) PCR analysis for the verification of the proper integration of the mutated Hfkbp12-gfp construct at the Affkbp12 native locus. Primers Fkbp12-1-promo-F and pUCGH-R (indicated by arrows) were used to amplify the 1446 bp PCR fragment from 10 recombinant strains (6 transformants are shown). Primers Hyg-Screen-F and Fkbp12-term-R (indicated by arrows) were used to amplify the 1054 bp PCR fragment from 10 recombinant strains (6 transformants are shown).

## Slide 14
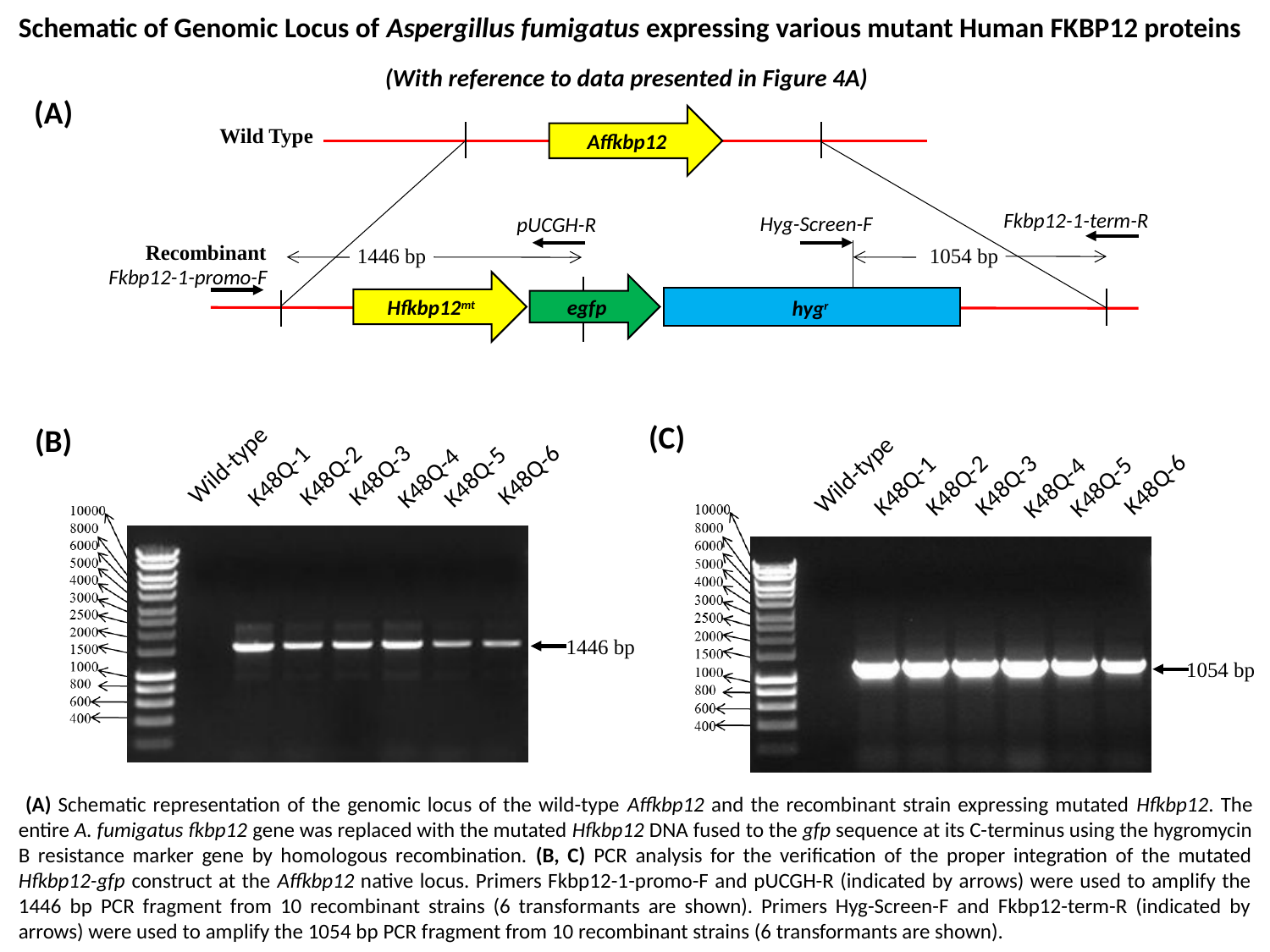

Schematic of Genomic Locus of Aspergillus fumigatus expressing various mutant Human FKBP12 proteins
(With reference to data presented in Figure 4A)
(A)
Affkbp12
Wild Type
Fkbp12-1-term-R
Hyg-Screen-F
pUCGH-R
Recombinant
 1446 bp
 1054 bp
Fkbp12-1-promo-F
Hfkbp12mt
egfp
hygr
(C)
 1054 bp
(B)
 1446 bp
Wild-type
K48Q-6
K48Q-3
K48Q-2
K48Q-1
K48Q-5
K48Q-4
Wild-type
K48Q-6
K48Q-3
K48Q-2
K48Q-1
K48Q-5
K48Q-4
 (A) Schematic representation of the genomic locus of the wild-type Affkbp12 and the recombinant strain expressing mutated Hfkbp12. The entire A. fumigatus fkbp12 gene was replaced with the mutated Hfkbp12 DNA fused to the gfp sequence at its C-terminus using the hygromycin B resistance marker gene by homologous recombination. (B, C) PCR analysis for the verification of the proper integration of the mutated Hfkbp12-gfp construct at the Affkbp12 native locus. Primers Fkbp12-1-promo-F and pUCGH-R (indicated by arrows) were used to amplify the 1446 bp PCR fragment from 10 recombinant strains (6 transformants are shown). Primers Hyg-Screen-F and Fkbp12-term-R (indicated by arrows) were used to amplify the 1054 bp PCR fragment from 10 recombinant strains (6 transformants are shown).

## Slide 15
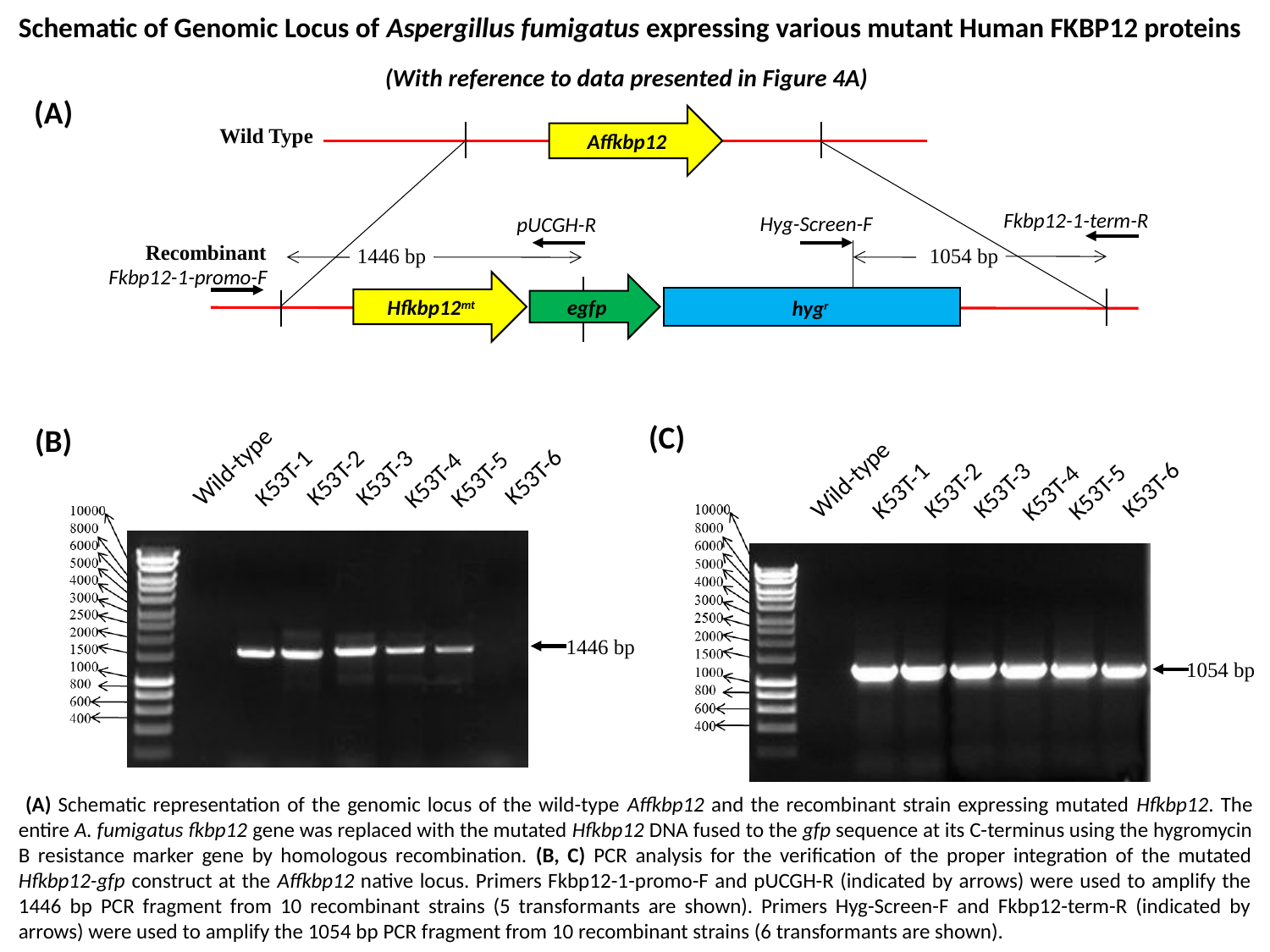

Schematic of Genomic Locus of Aspergillus fumigatus expressing various mutant Human FKBP12 proteins
(With reference to data presented in Figure 4A)
(A)
Affkbp12
Wild Type
Fkbp12-1-term-R
Hyg-Screen-F
pUCGH-R
Recombinant
 1446 bp
 1054 bp
Fkbp12-1-promo-F
Hfkbp12mt
egfp
hygr
(C)
 1054 bp
(B)
 1446 bp
Wild-type
K53T-6
K53T-3
K53T-2
K53T-1
K53T-5
K53T-4
Wild-type
K53T-6
K53T-3
K53T-2
K53T-1
K53T-5
K53T-4
 (A) Schematic representation of the genomic locus of the wild-type Affkbp12 and the recombinant strain expressing mutated Hfkbp12. The entire A. fumigatus fkbp12 gene was replaced with the mutated Hfkbp12 DNA fused to the gfp sequence at its C-terminus using the hygromycin B resistance marker gene by homologous recombination. (B, C) PCR analysis for the verification of the proper integration of the mutated Hfkbp12-gfp construct at the Affkbp12 native locus. Primers Fkbp12-1-promo-F and pUCGH-R (indicated by arrows) were used to amplify the 1446 bp PCR fragment from 10 recombinant strains (5 transformants are shown). Primers Hyg-Screen-F and Fkbp12-term-R (indicated by arrows) were used to amplify the 1054 bp PCR fragment from 10 recombinant strains (6 transformants are shown).

## Slide 16
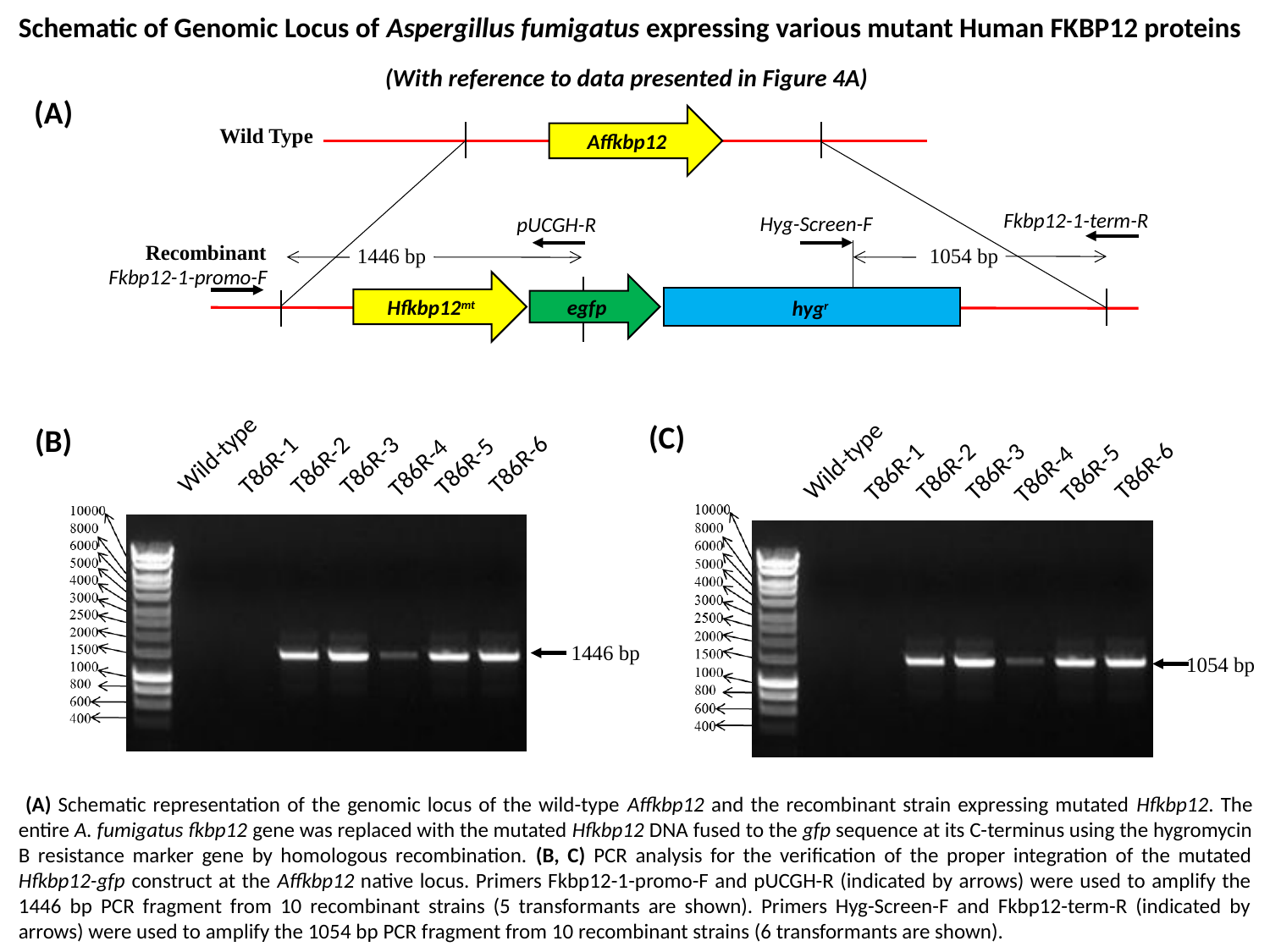

Schematic of Genomic Locus of Aspergillus fumigatus expressing various mutant Human FKBP12 proteins
(With reference to data presented in Figure 4A)
(A)
Affkbp12
Wild Type
Fkbp12-1-term-R
Hyg-Screen-F
pUCGH-R
Recombinant
 1446 bp
 1054 bp
Fkbp12-1-promo-F
Hfkbp12mt
egfp
hygr
(C)
 1054 bp
(B)
 1446 bp
Wild-type
T86R-6
T86R-3
T86R-2
T86R-1
T86R-5
T86R-4
Wild-type
T86R-6
T86R-3
T86R-2
T86R-1
T86R-5
T86R-4
 (A) Schematic representation of the genomic locus of the wild-type Affkbp12 and the recombinant strain expressing mutated Hfkbp12. The entire A. fumigatus fkbp12 gene was replaced with the mutated Hfkbp12 DNA fused to the gfp sequence at its C-terminus using the hygromycin B resistance marker gene by homologous recombination. (B, C) PCR analysis for the verification of the proper integration of the mutated Hfkbp12-gfp construct at the Affkbp12 native locus. Primers Fkbp12-1-promo-F and pUCGH-R (indicated by arrows) were used to amplify the 1446 bp PCR fragment from 10 recombinant strains (5 transformants are shown). Primers Hyg-Screen-F and Fkbp12-term-R (indicated by arrows) were used to amplify the 1054 bp PCR fragment from 10 recombinant strains (6 transformants are shown).

## Slide 17
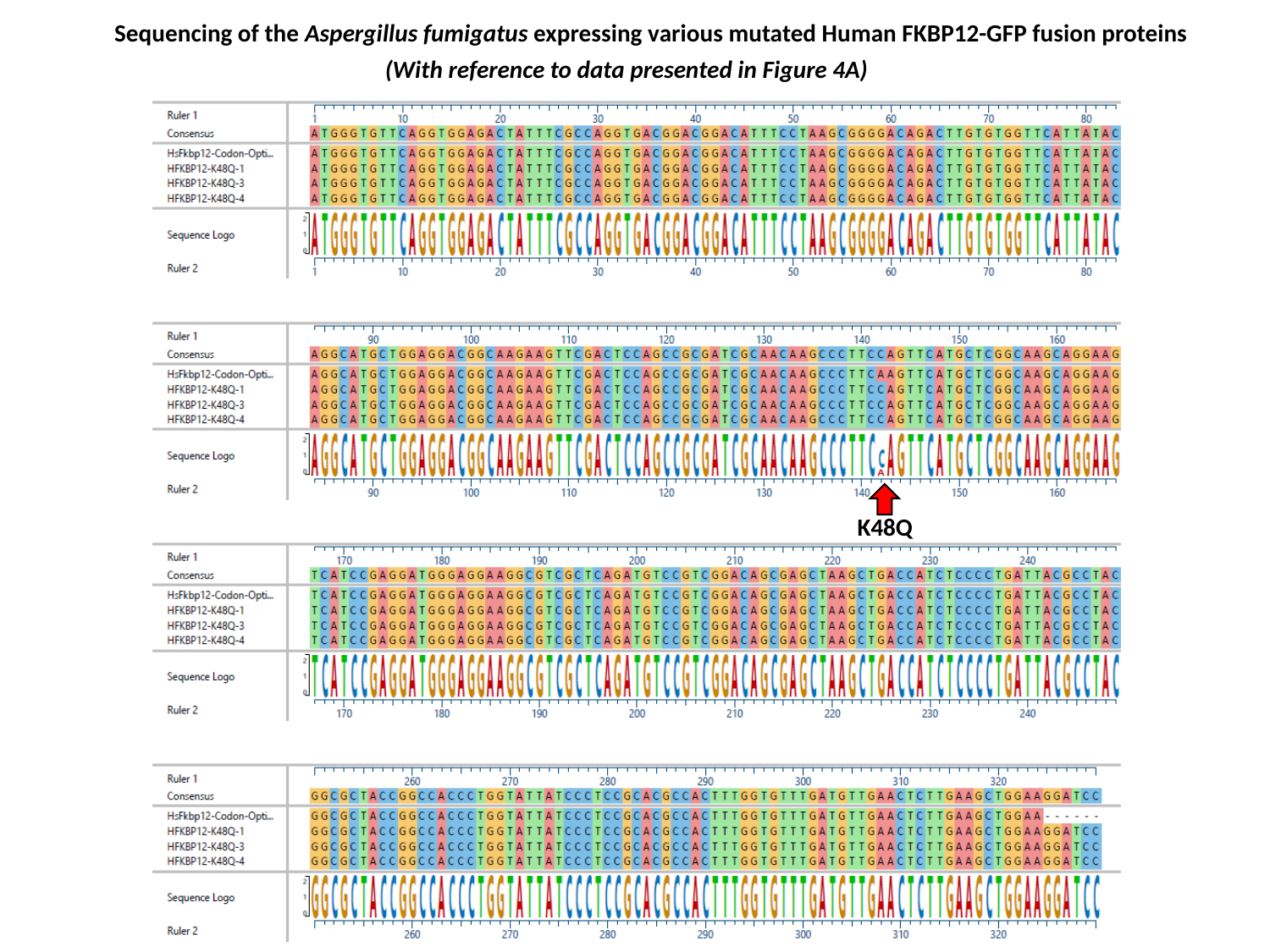

Sequencing of the Aspergillus fumigatus expressing various mutated Human FKBP12-GFP fusion proteins
(With reference to data presented in Figure 4A)
K48Q

## Slide 18
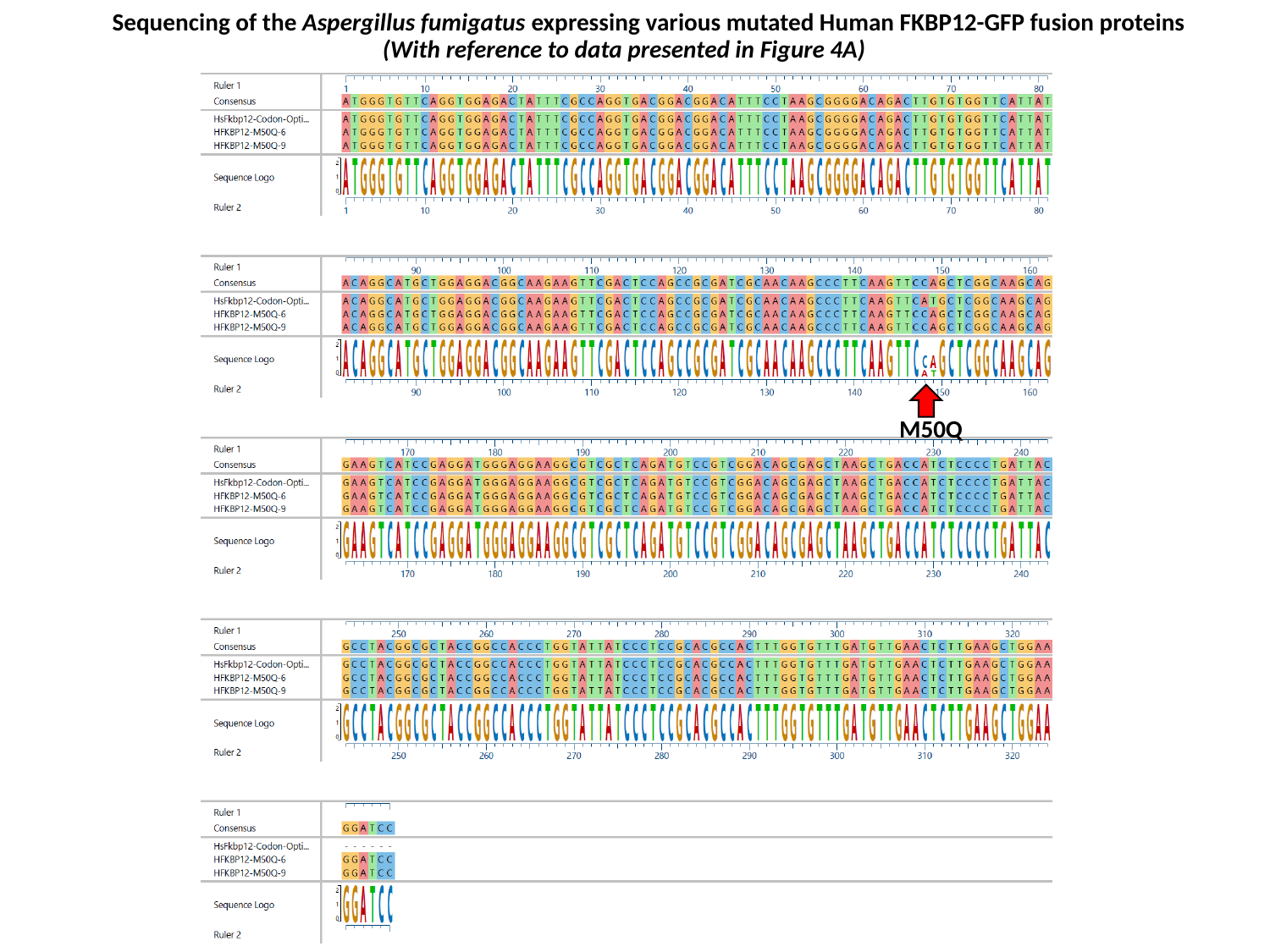

Sequencing of the Aspergillus fumigatus expressing various mutated Human FKBP12-GFP fusion proteins
(With reference to data presented in Figure 4A)
M50Q

## Slide 19
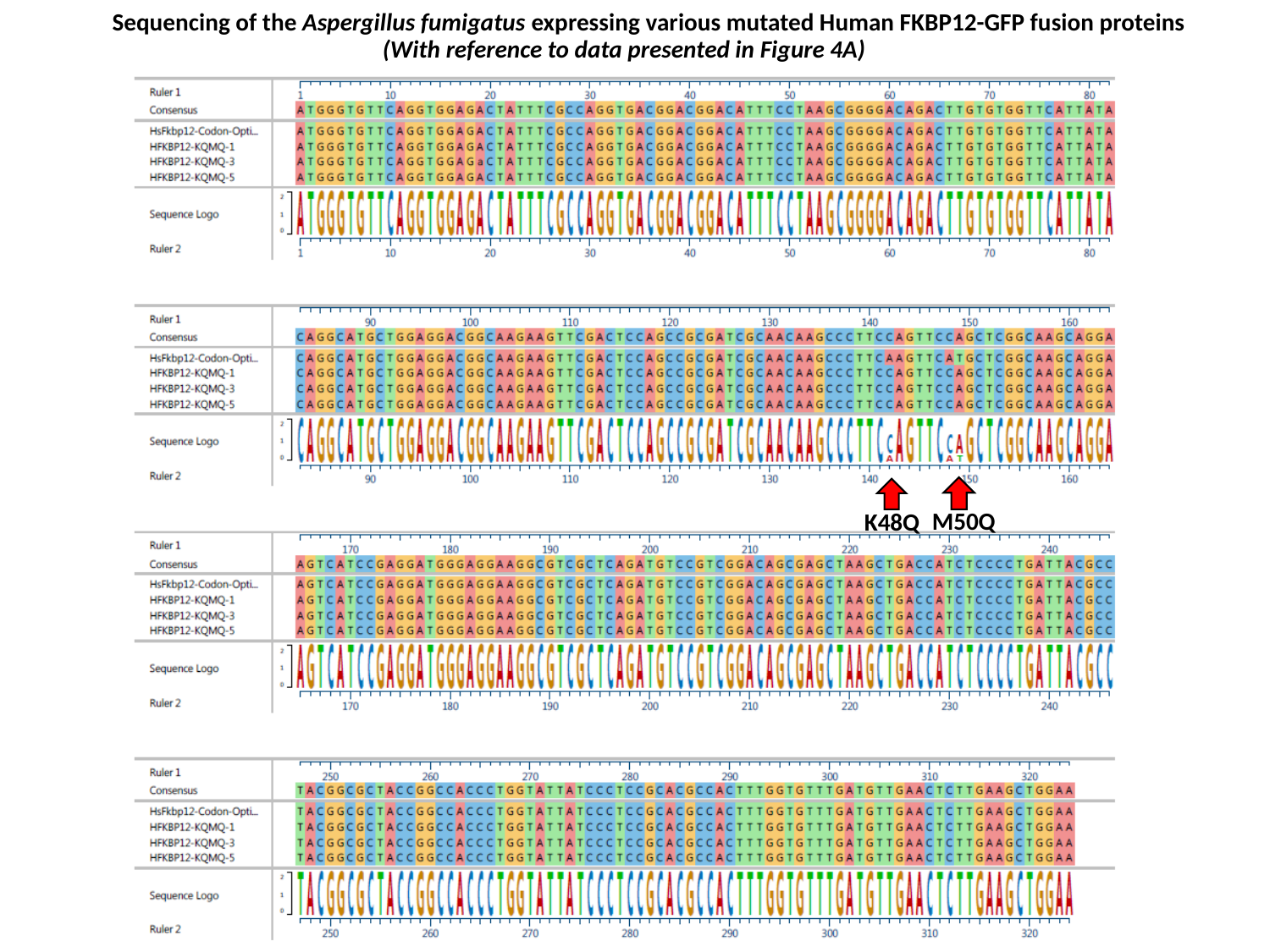

Sequencing of the Aspergillus fumigatus expressing various mutated Human FKBP12-GFP fusion proteins
(With reference to data presented in Figure 4A)
M50Q
K48Q

## Slide 20
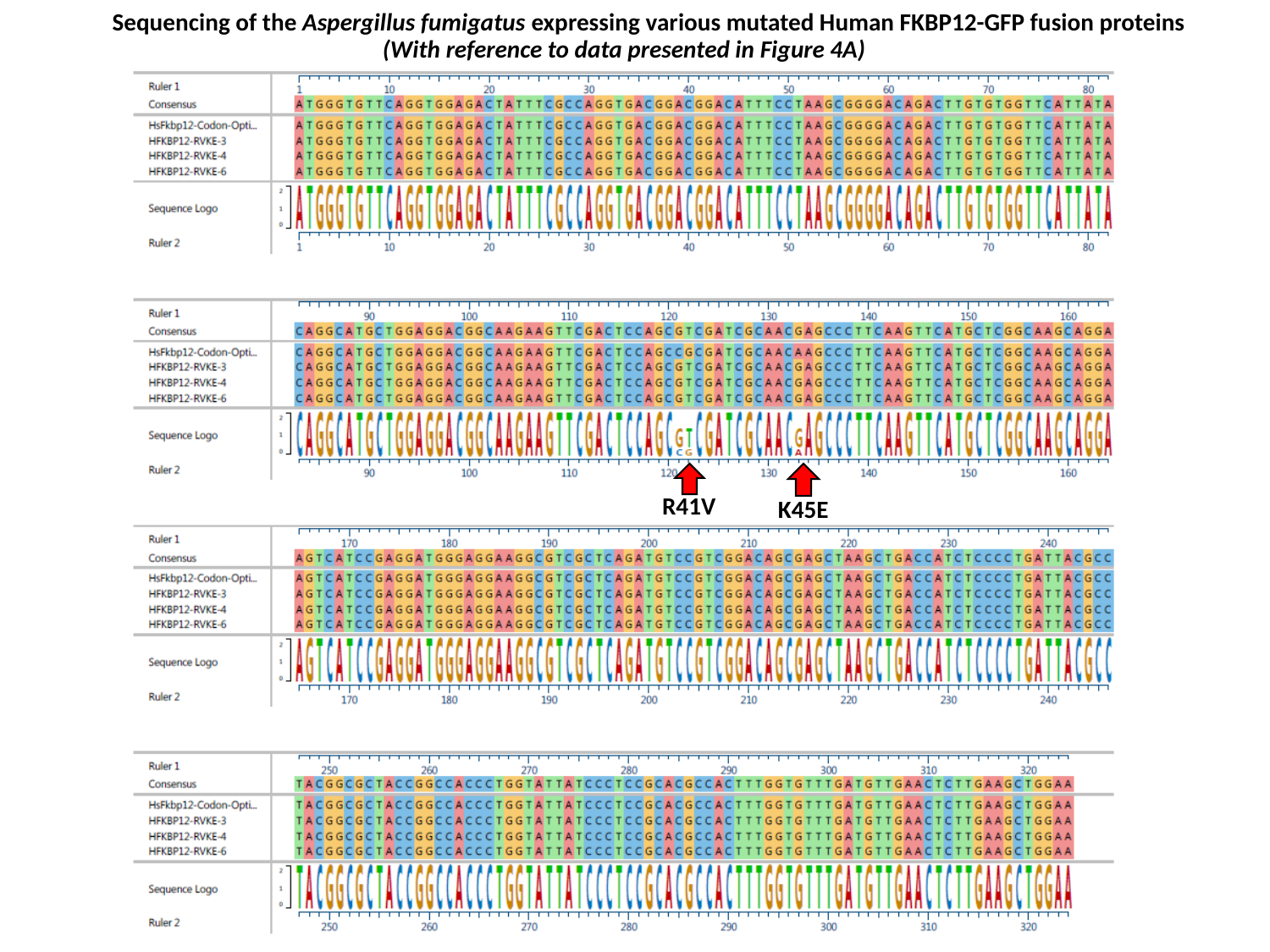

Sequencing of the Aspergillus fumigatus expressing various mutated Human FKBP12-GFP fusion proteins
(With reference to data presented in Figure 4A)
R41V
K45E

## Slide 21
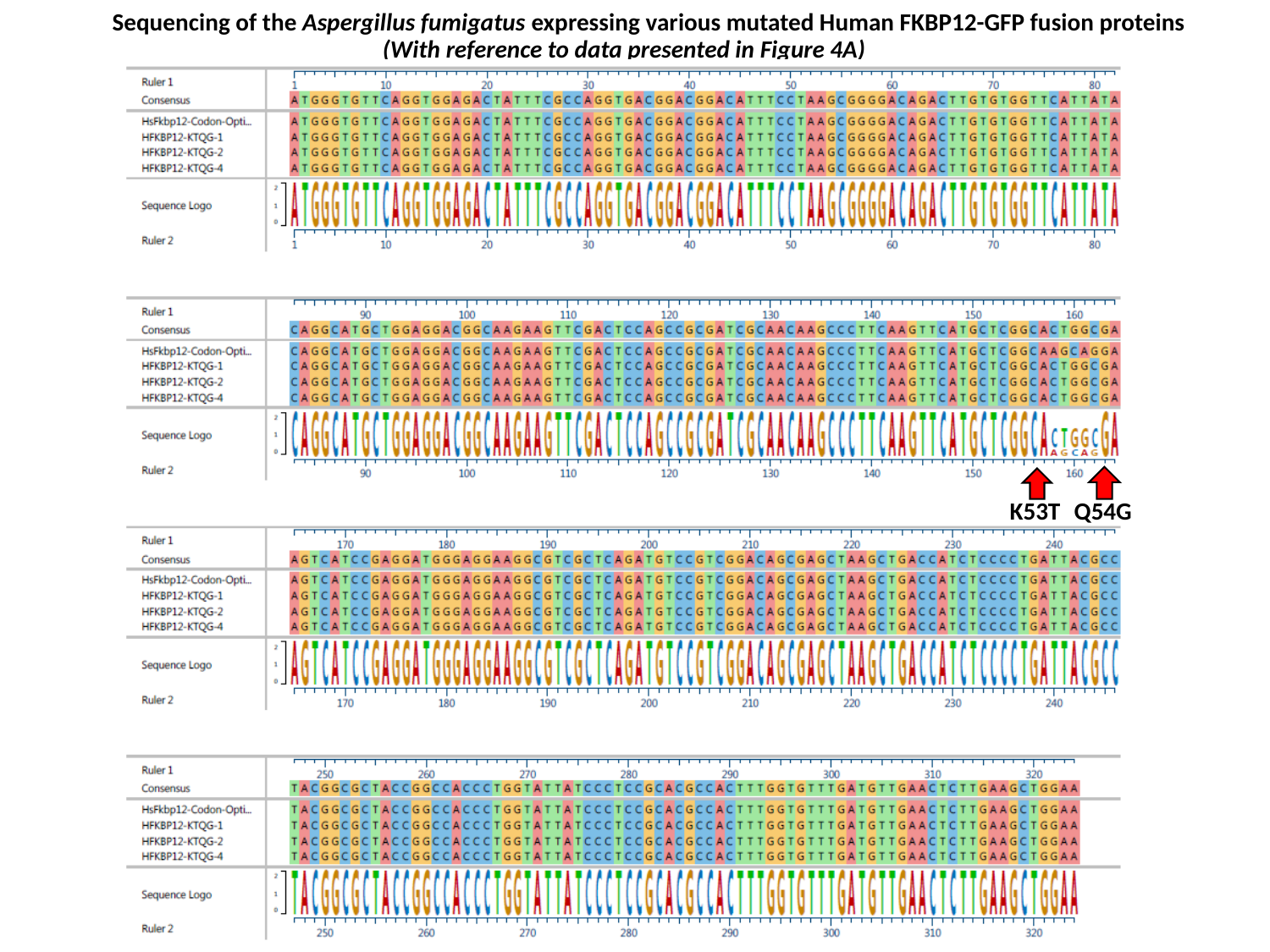

Sequencing of the Aspergillus fumigatus expressing various mutated Human FKBP12-GFP fusion proteins
(With reference to data presented in Figure 4A)
Q54G
K53T

## Slide 22
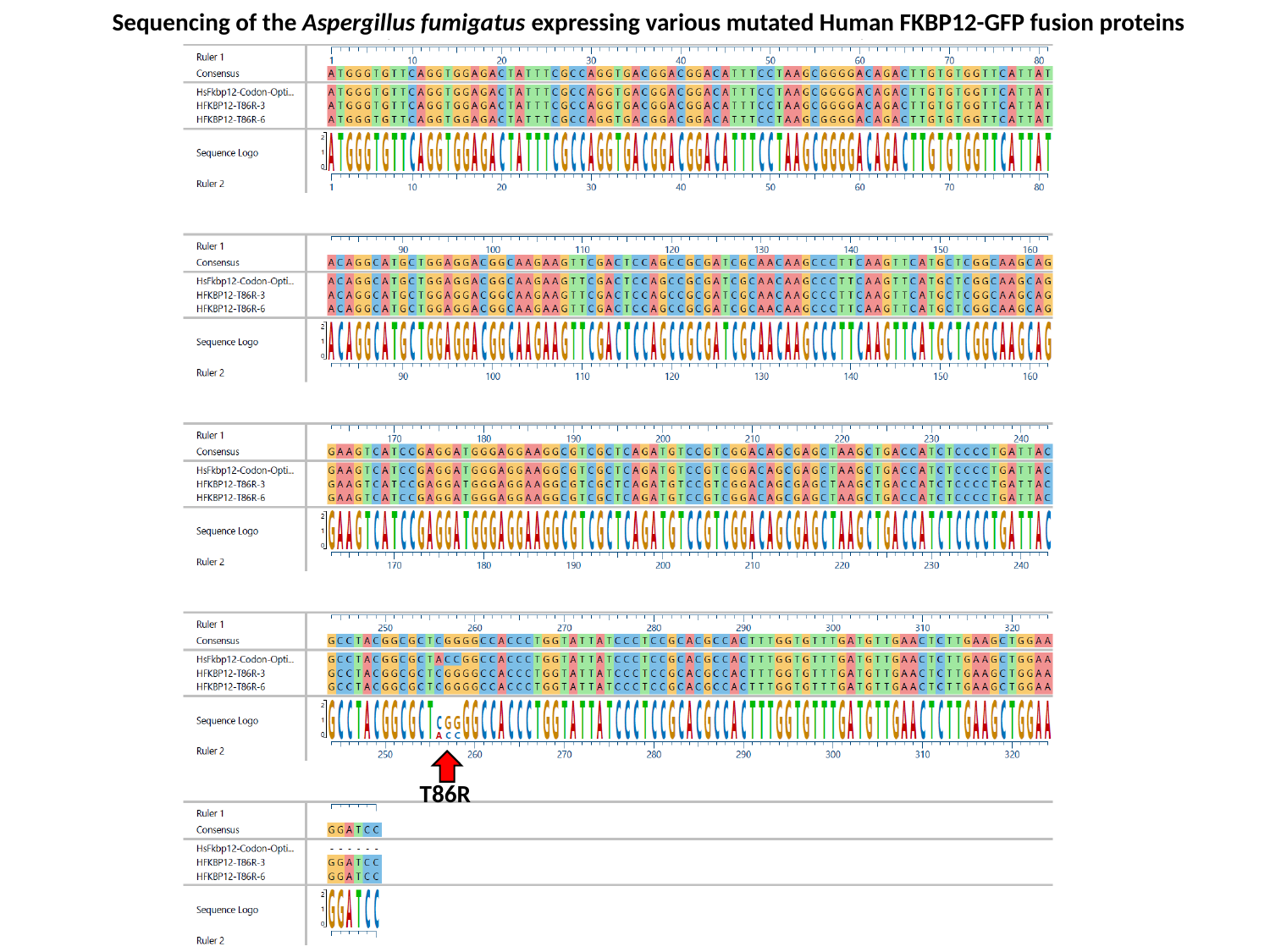

Sequencing of the Aspergillus fumigatus expressing various mutated Human FKBP12-GFP fusion proteins
(With reference to data presented in Figure 4A)
T86R

## Slide 23
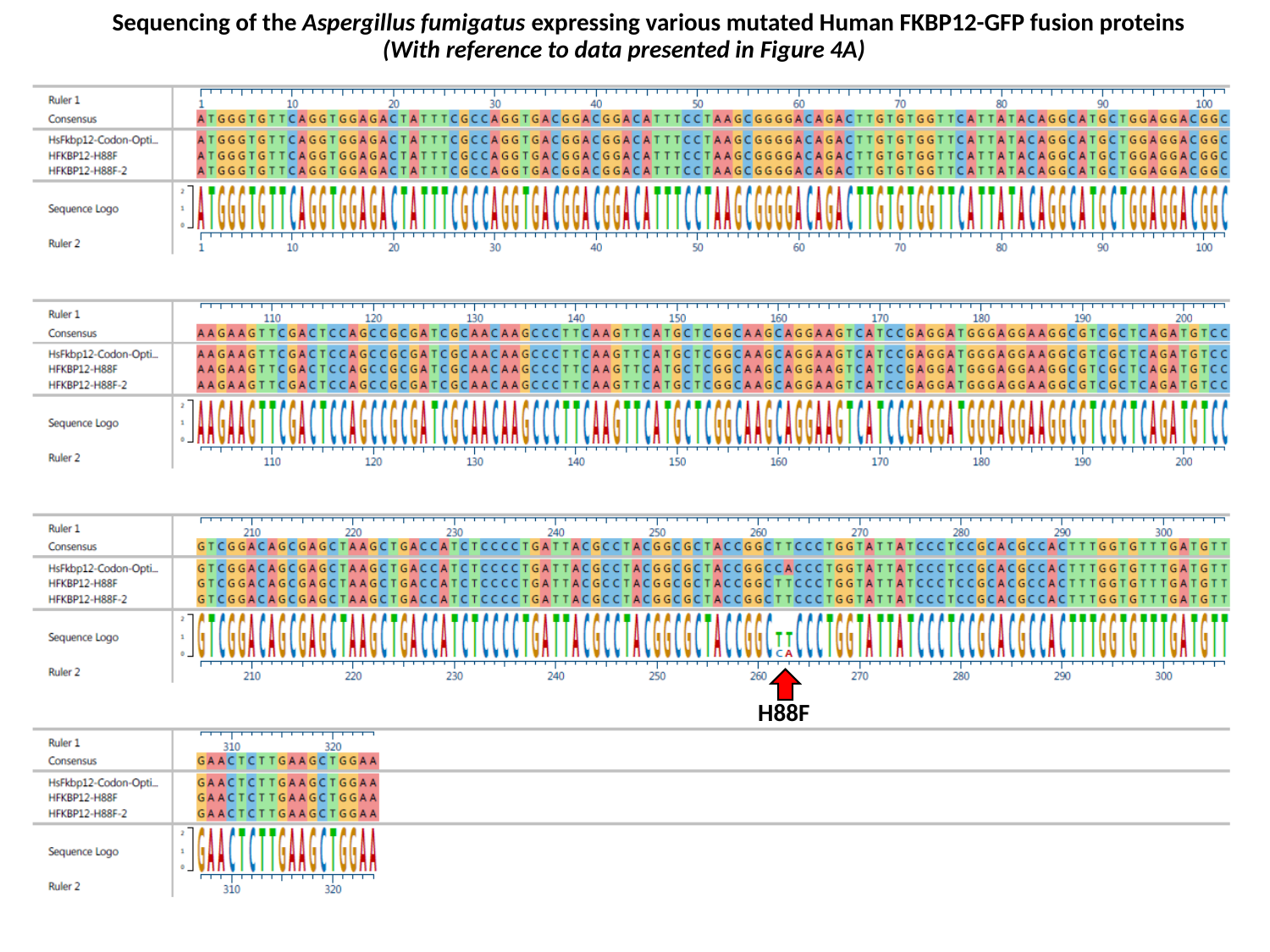

Sequencing of the Aspergillus fumigatus expressing various mutated Human FKBP12-GFP fusion proteins
(With reference to data presented in Figure 4A)
H88F

## Slide 24
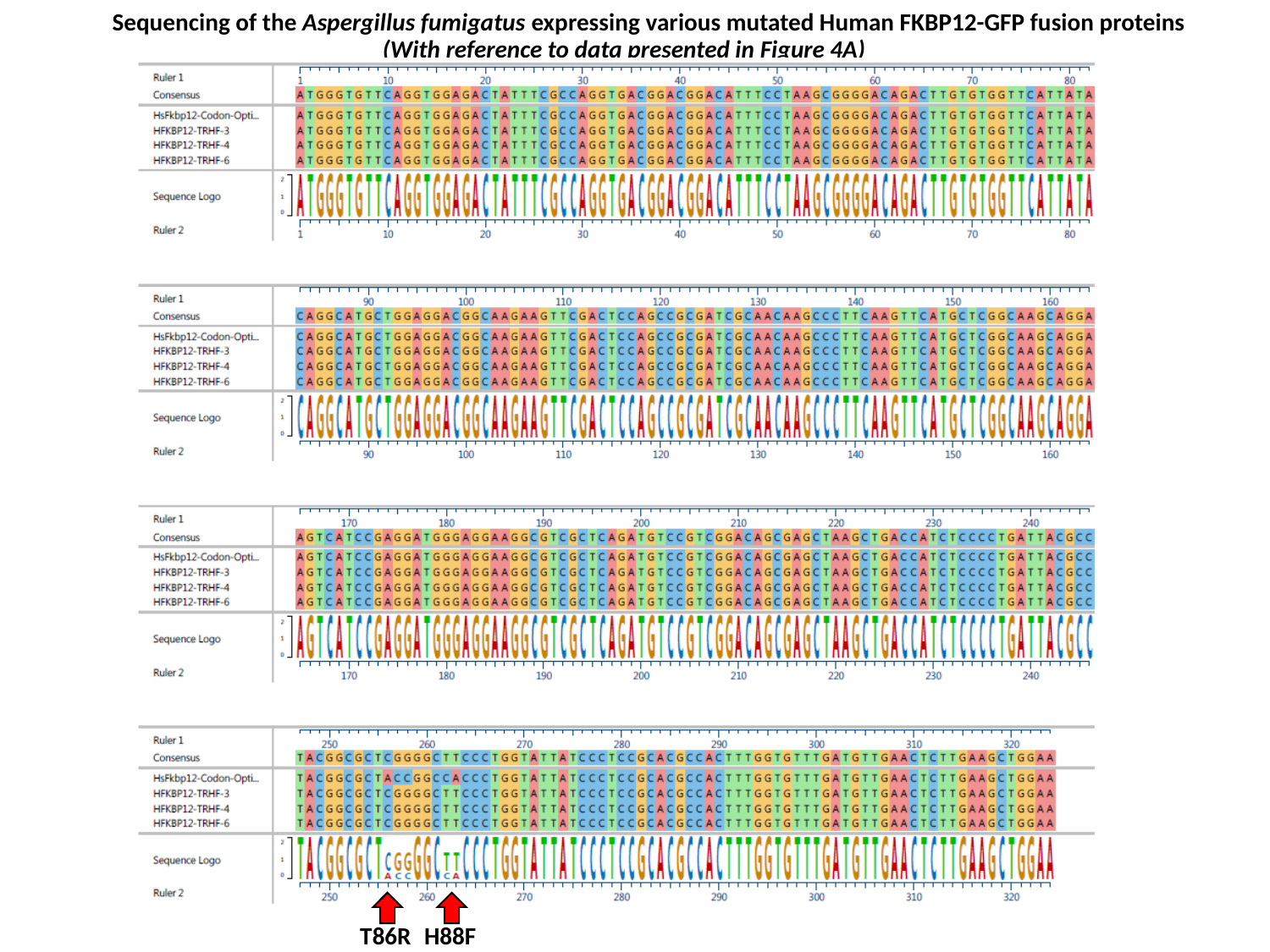

Sequencing of the Aspergillus fumigatus expressing various mutated Human FKBP12-GFP fusion proteins
(With reference to data presented in Figure 4A)
T86R
H88F

## Slide 25
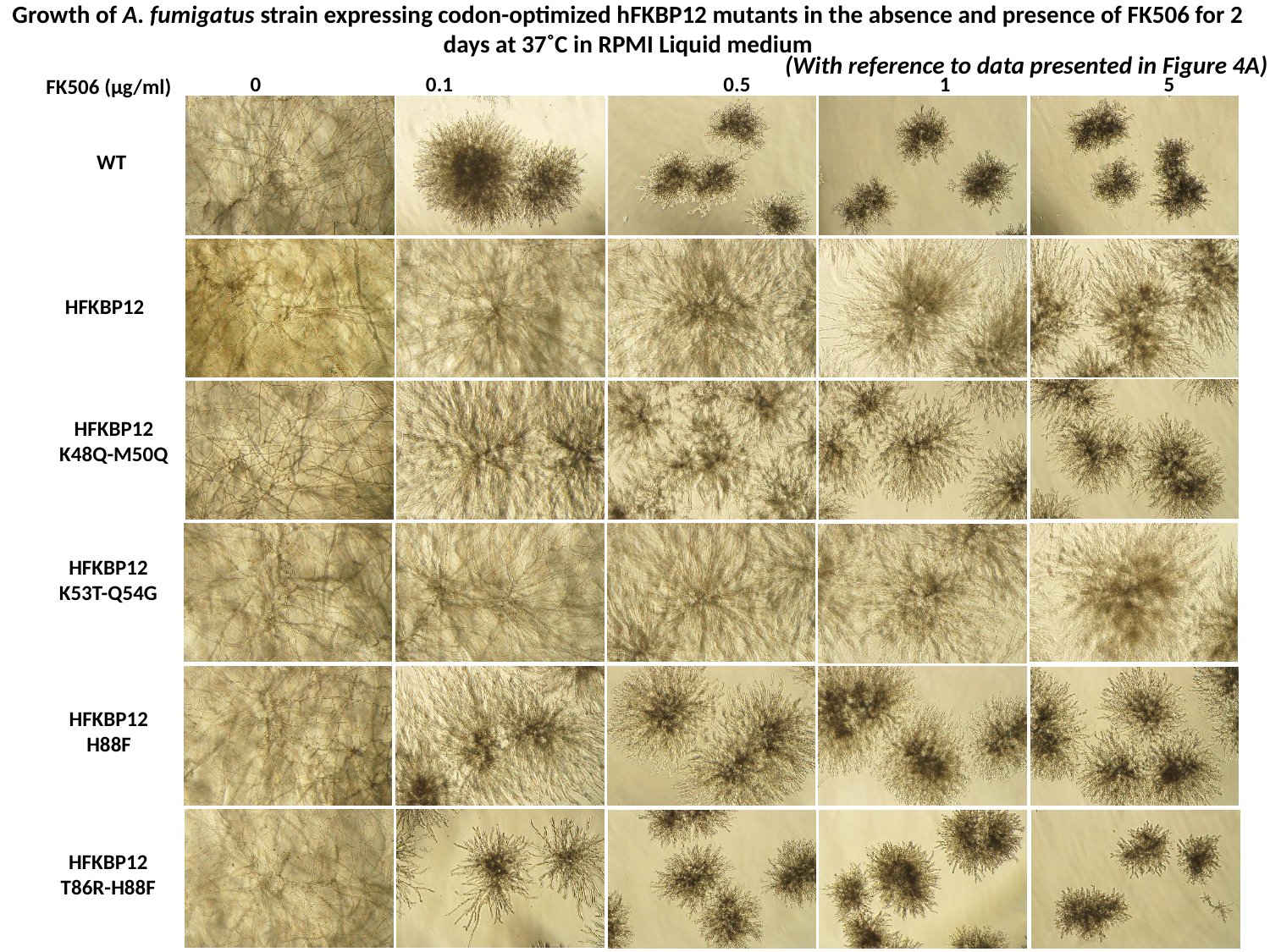

Growth of A. fumigatus strain expressing codon-optimized hFKBP12 mutants in the absence and presence of FK506 for 2 days at 37˚C in RPMI Liquid medium
(With reference to data presented in Figure 4A)
0 0.1		 0.5 1 5
FK506 (µg/ml)
WT
HFKBP12
HFKBP12
K48Q-M50Q
HFKBP12
K53T-Q54G
HFKBP12
H88F
HFKBP12
T86R-H88F

## Slide 26
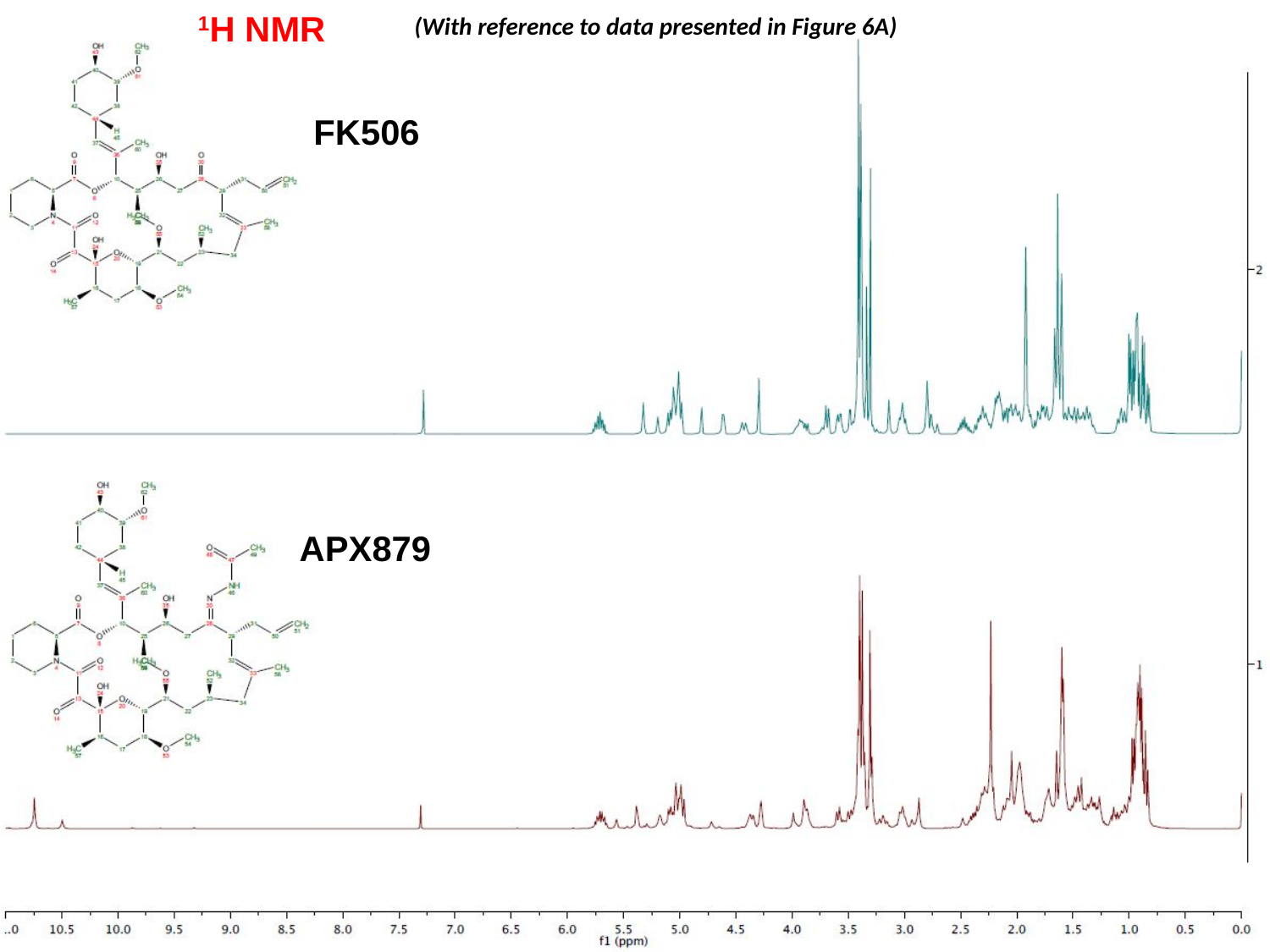

1H NMR
(With reference to data presented in Figure 6A)
FK506
APX879

## Slide 27
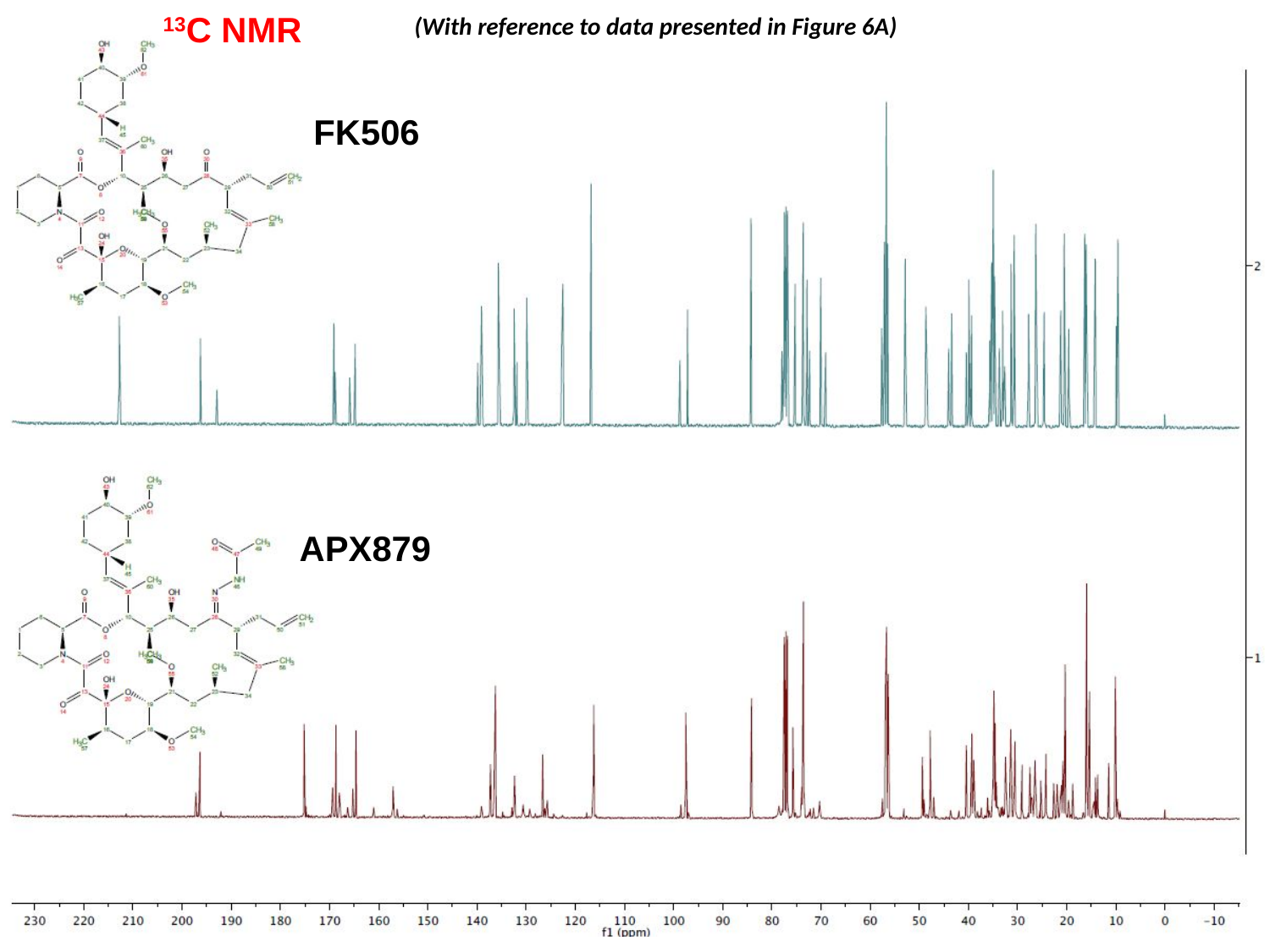

13C NMR
(With reference to data presented in Figure 6A)
FK506
APX879

## Slide 28
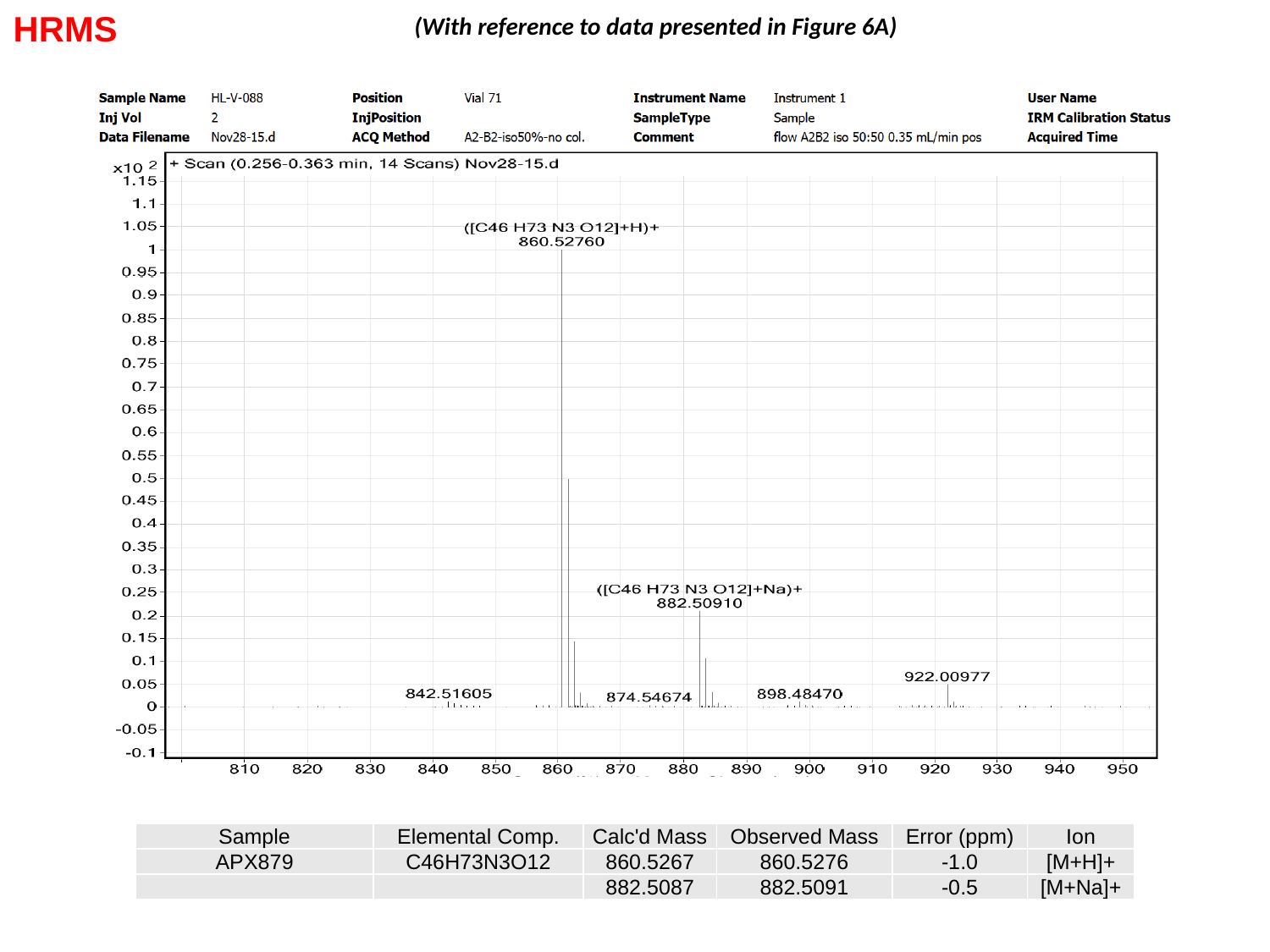

HRMS
(With reference to data presented in Figure 6A)
| Sample | Elemental Comp. | Calc'd Mass | Observed Mass | Error (ppm) | Ion |
| --- | --- | --- | --- | --- | --- |
| APX879 | C46H73N3O12 | 860.5267 | 860.5276 | -1.0 | [M+H]+ |
| | | 882.5087 | 882.5091 | -0.5 | [M+Na]+ |

## Slide 29
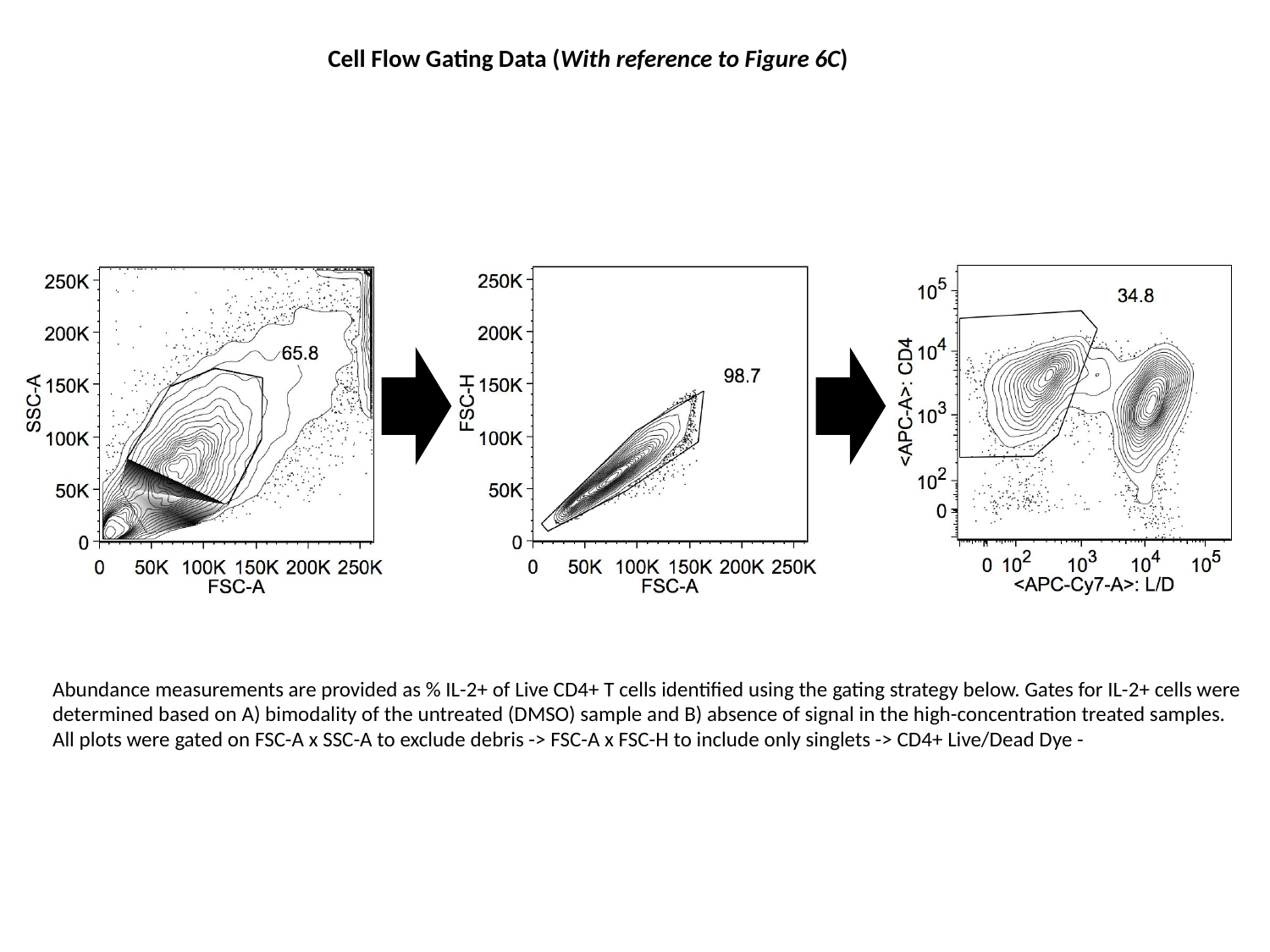

Cell Flow Gating Data (With reference to Figure 6C)
Abundance measurements are provided as % IL-2+ of Live CD4+ T cells identified using the gating strategy below. Gates for IL-2+ cells were determined based on A) bimodality of the untreated (DMSO) sample and B) absence of signal in the high-concentration treated samples. All plots were gated on FSC-A x SSC-A to exclude debris -> FSC-A x FSC-H to include only singlets -> CD4+ Live/Dead Dye -

## Slide 30
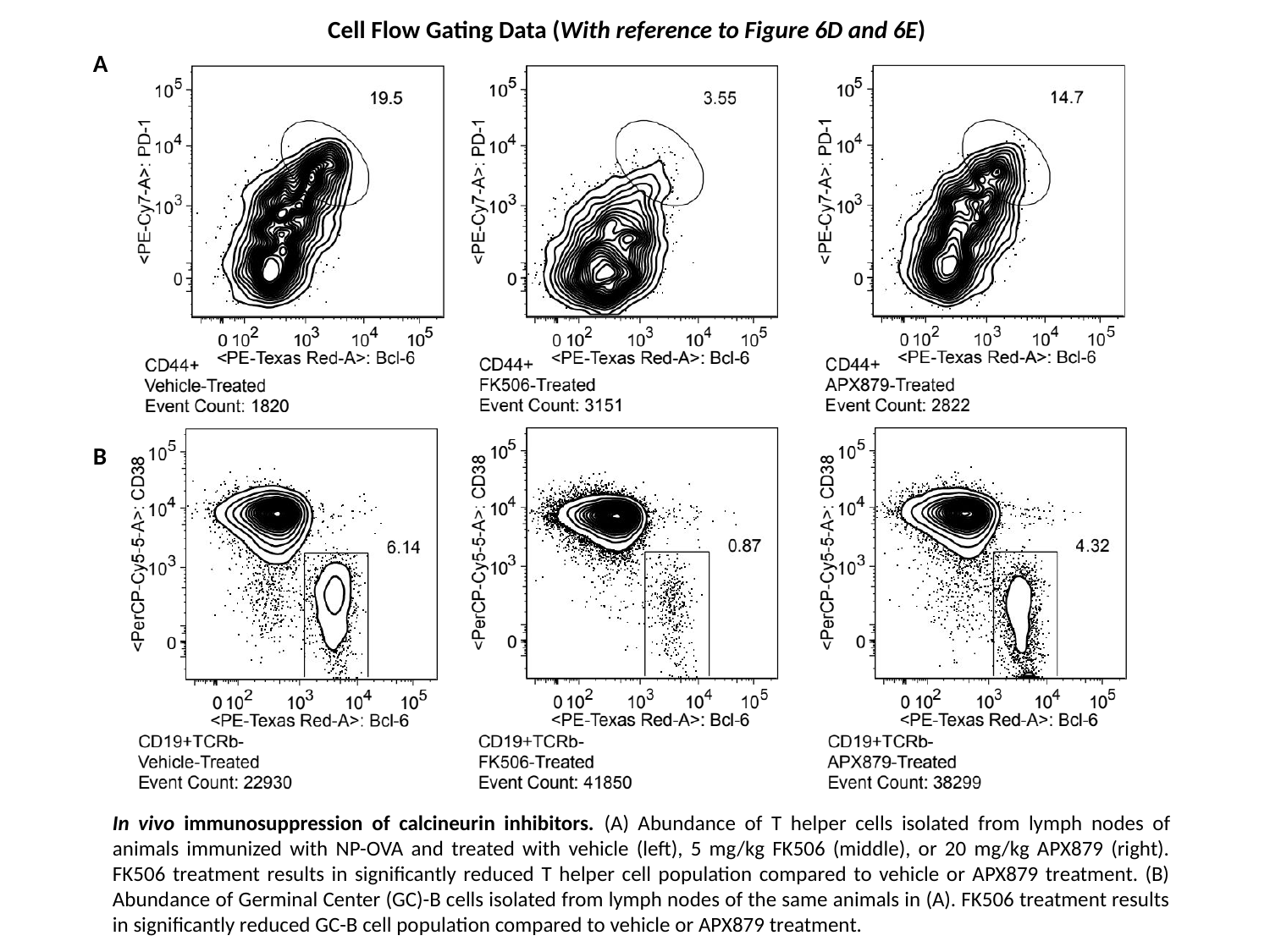

Cell Flow Gating Data (With reference to Figure 6D and 6E)
A
B
In vivo immunosuppression of calcineurin inhibitors. (A) Abundance of T helper cells isolated from lymph nodes of animals immunized with NP-OVA and treated with vehicle (left), 5 mg/kg FK506 (middle), or 20 mg/kg APX879 (right). FK506 treatment results in significantly reduced T helper cell population compared to vehicle or APX879 treatment. (B) Abundance of Germinal Center (GC)-B cells isolated from lymph nodes of the same animals in (A). FK506 treatment results in significantly reduced GC-B cell population compared to vehicle or APX879 treatment.

## Slide 31
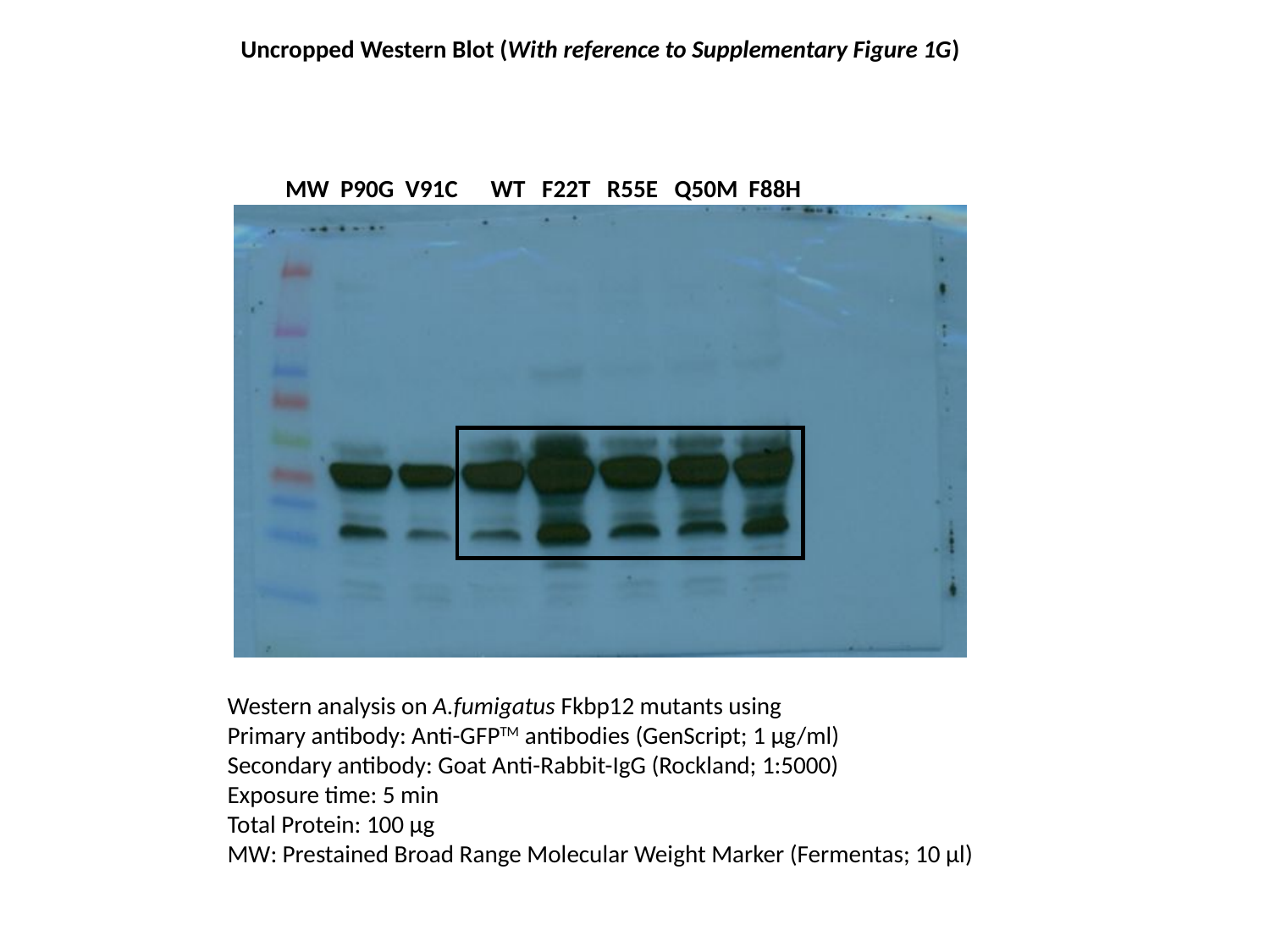

Uncropped Western Blot (With reference to Supplementary Figure 1G)
MW P90G V91C WT F22T R55E Q50M F88H
Western analysis on A.fumigatus Fkbp12 mutants using
Primary antibody: Anti-GFPTM antibodies (GenScript; 1 µg/ml)
Secondary antibody: Goat Anti-Rabbit-IgG (Rockland; 1:5000)
Exposure time: 5 min
Total Protein: 100 µg
MW: Prestained Broad Range Molecular Weight Marker (Fermentas; 10 µl)

## Slide 32
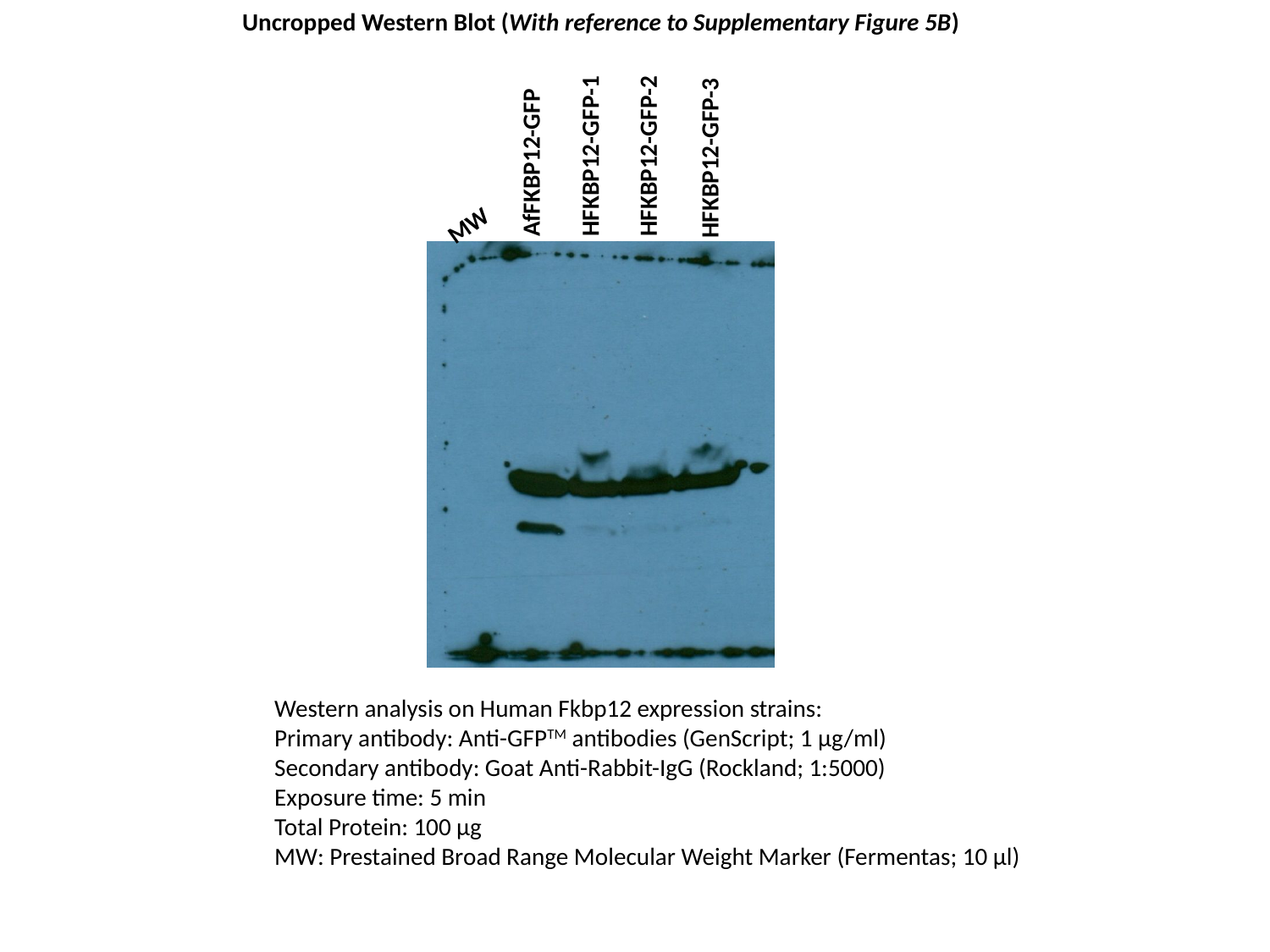

Uncropped Western Blot (With reference to Supplementary Figure 5B)
HFKBP12-GFP-1
HFKBP12-GFP-2
HFKBP12-GFP-3
AfFKBP12-GFP
MW
Western analysis on Human Fkbp12 expression strains:
Primary antibody: Anti-GFPTM antibodies (GenScript; 1 µg/ml)
Secondary antibody: Goat Anti-Rabbit-IgG (Rockland; 1:5000)
Exposure time: 5 min
Total Protein: 100 µg
MW: Prestained Broad Range Molecular Weight Marker (Fermentas; 10 µl)

## Slide 33
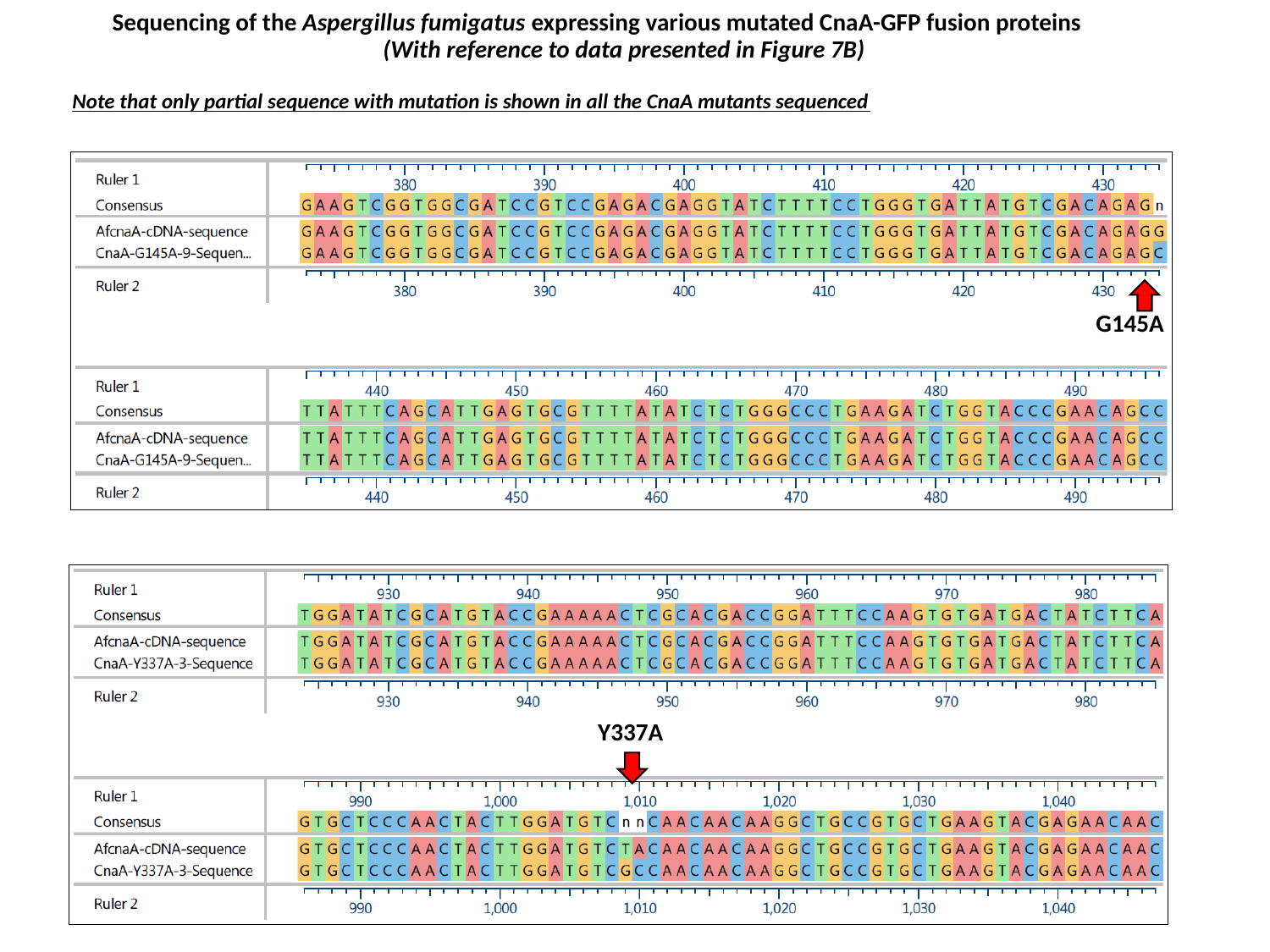

Sequencing of the Aspergillus fumigatus expressing various mutated CnaA-GFP fusion proteins
(With reference to data presented in Figure 7B)
Note that only partial sequence with mutation is shown in all the CnaA mutants sequenced
G145A
Y337A

## Slide 34
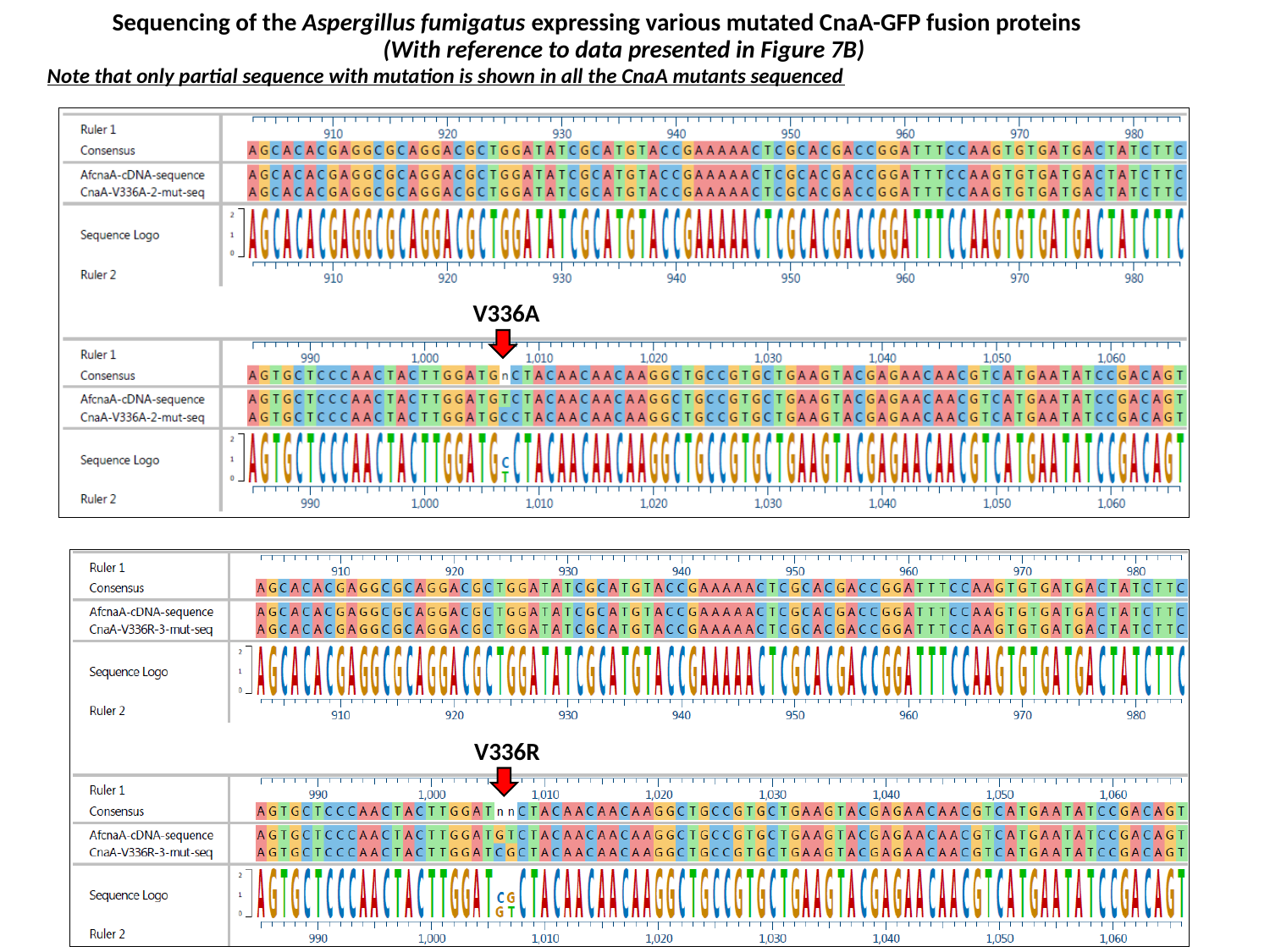

Sequencing of the Aspergillus fumigatus expressing various mutated CnaA-GFP fusion proteins
(With reference to data presented in Figure 7B)
Note that only partial sequence with mutation is shown in all the CnaA mutants sequenced
V336A
V336R

## Slide 35
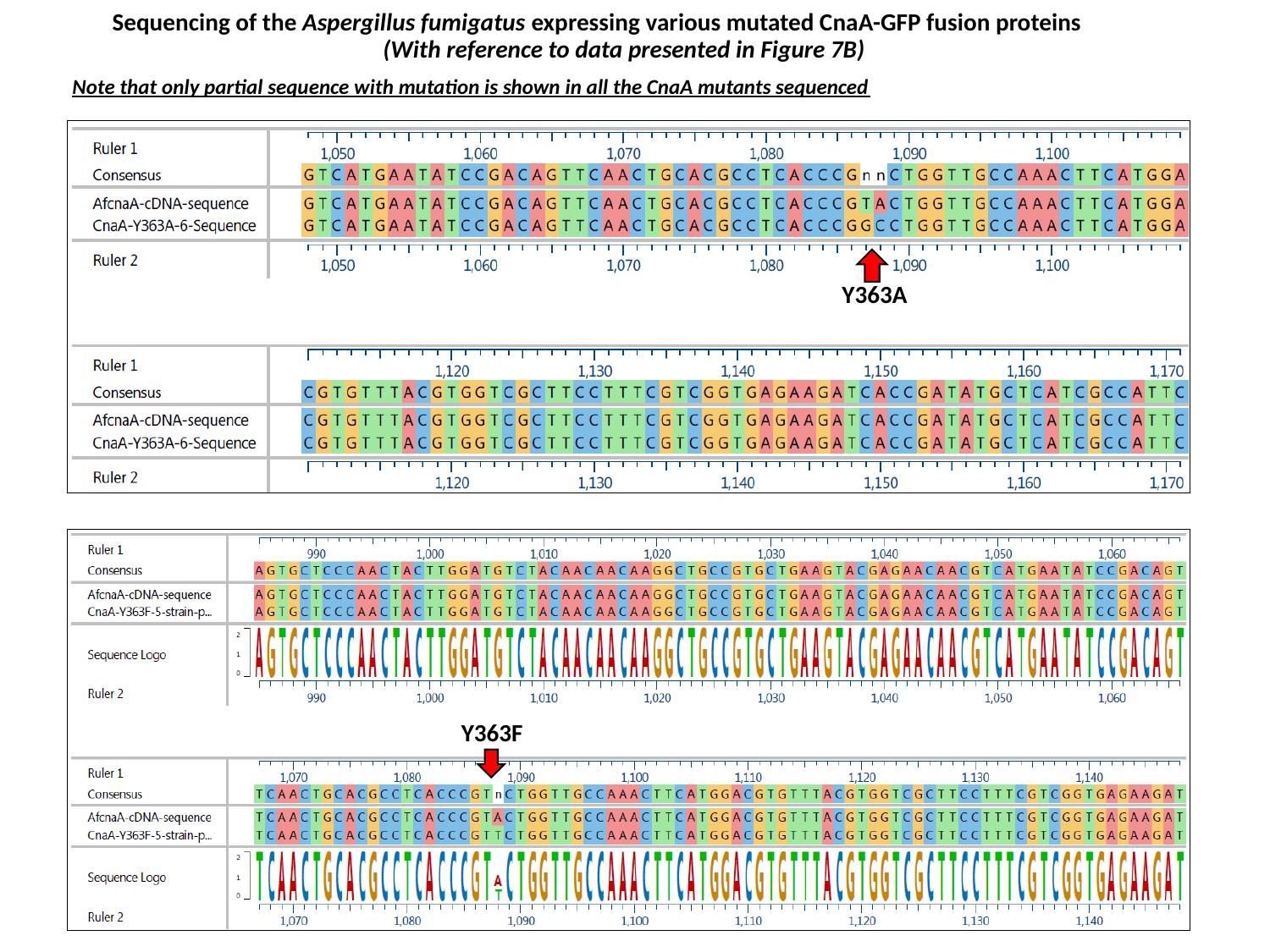

Sequencing of the Aspergillus fumigatus expressing various mutated CnaA-GFP fusion proteins
(With reference to data presented in Figure 7B)
Note that only partial sequence with mutation is shown in all the CnaA mutants sequenced
Y363A
Y363F

## Slide 36
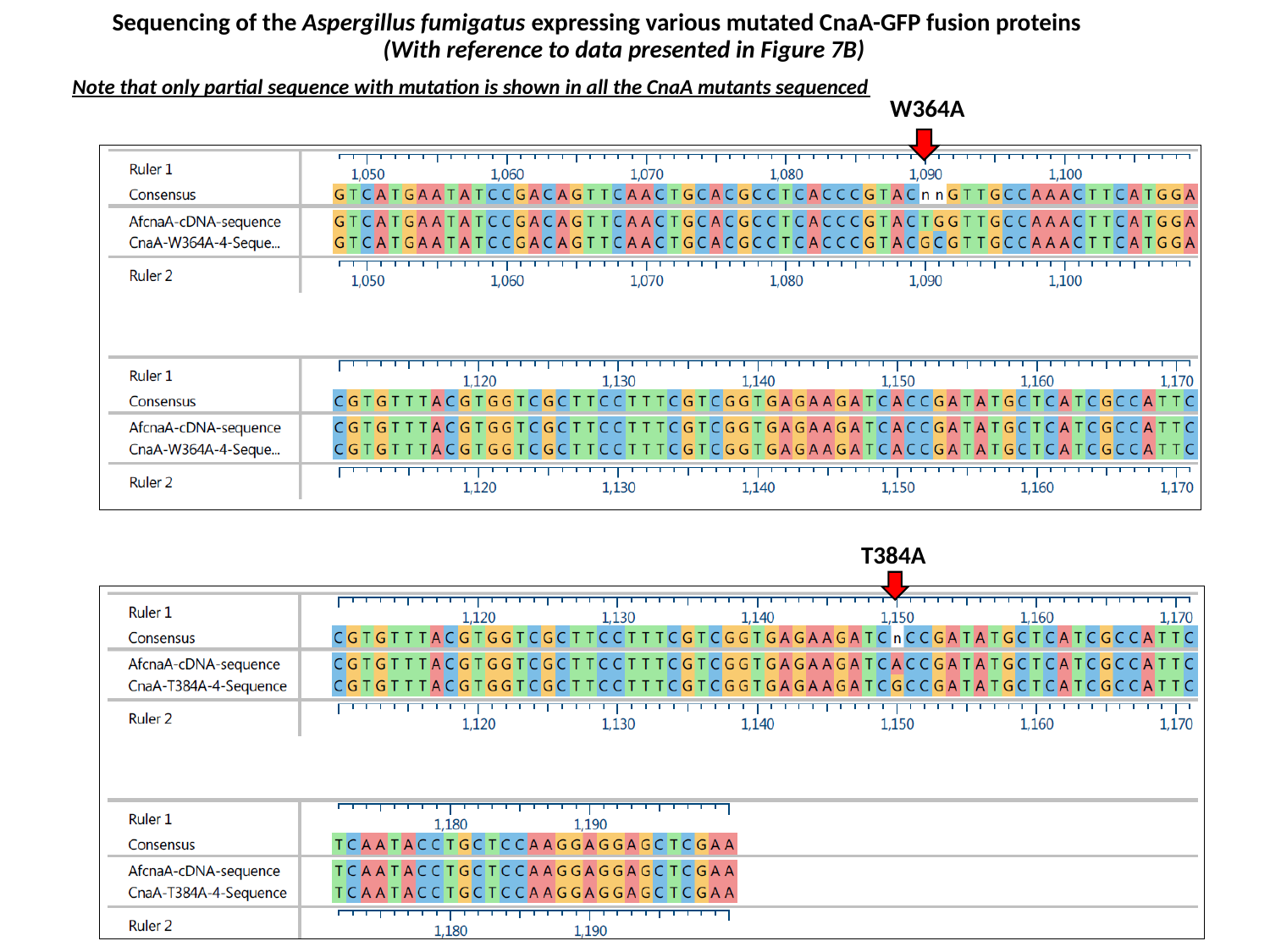

Sequencing of the Aspergillus fumigatus expressing various mutated CnaA-GFP fusion proteins
(With reference to data presented in Figure 7B)
Note that only partial sequence with mutation is shown in all the CnaA mutants sequenced
W364A
T384A

## Slide 37
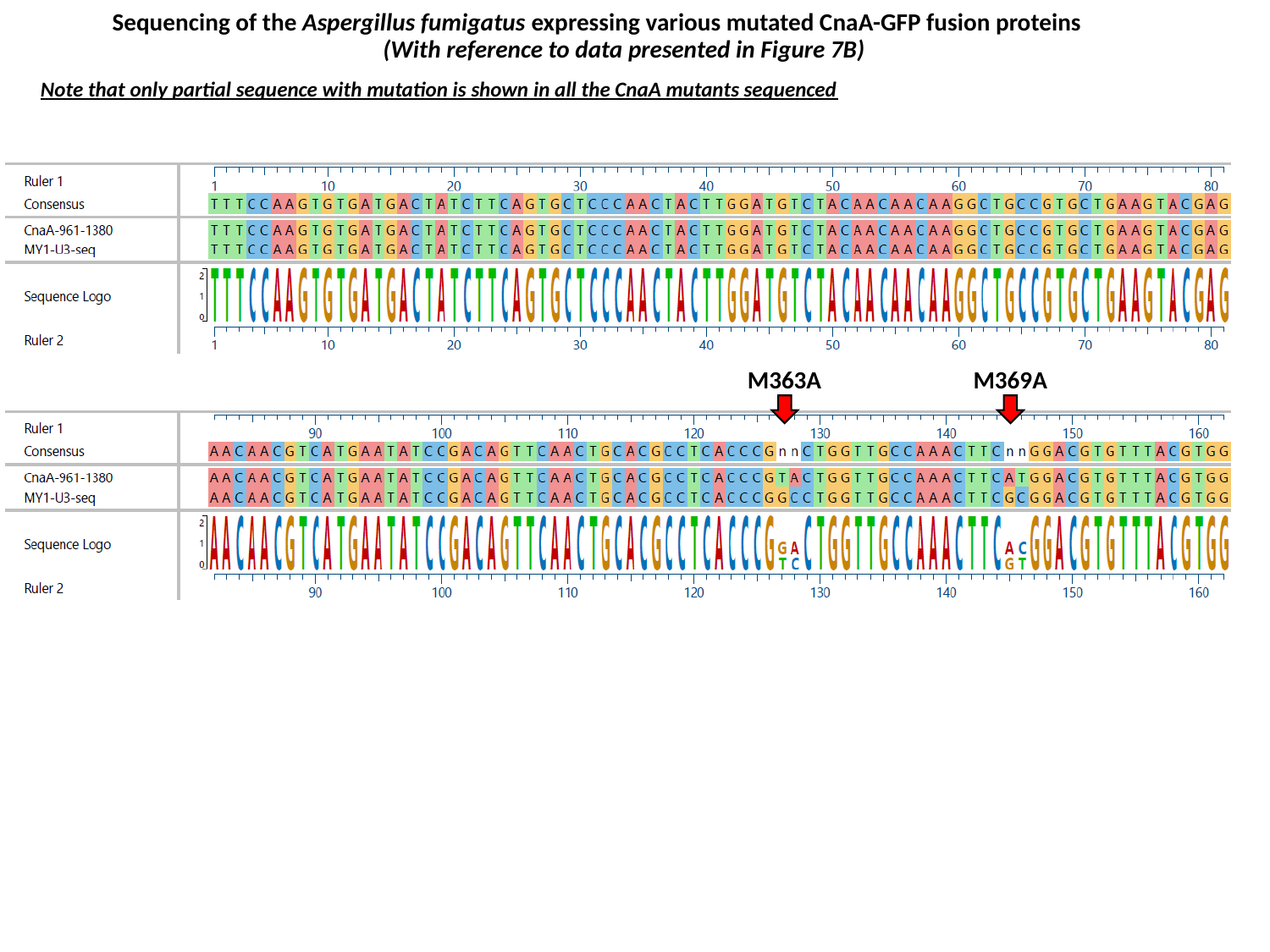

Sequencing of the Aspergillus fumigatus expressing various mutated CnaA-GFP fusion proteins
(With reference to data presented in Figure 7B)
Note that only partial sequence with mutation is shown in all the CnaA mutants sequenced
M363A
M369A

## Slide 38
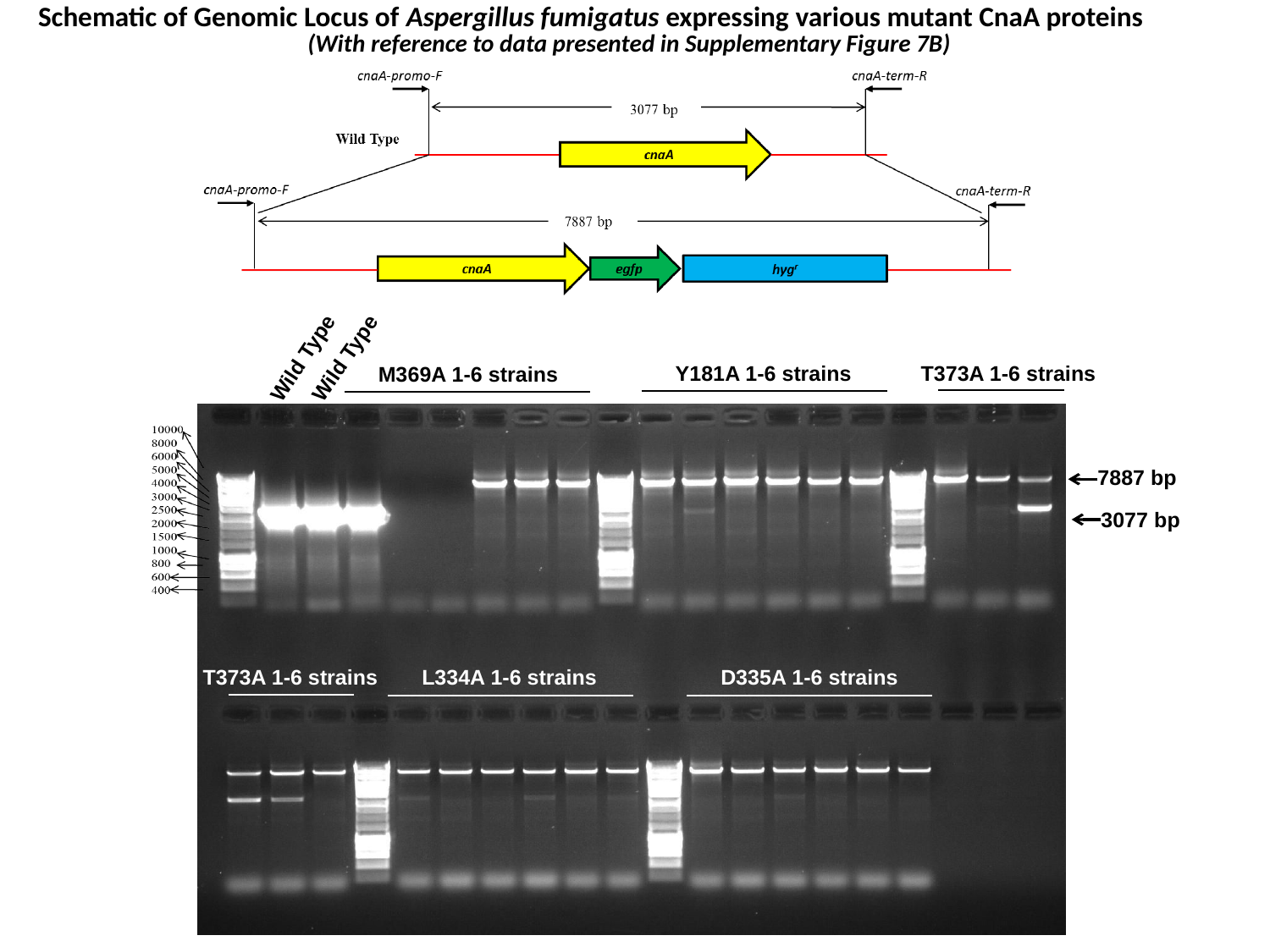

Schematic of Genomic Locus of Aspergillus fumigatus expressing various mutant CnaA proteins
(With reference to data presented in Supplementary Figure 7B)
Wild Type
Wild Type
T373A 1-6 strains
Y181A 1-6 strains
M369A 1-6 strains
T373A 1-6 strains
L334A 1-6 strains
D335A 1-6 strains
7887 bp
3077 bp

## Slide 39
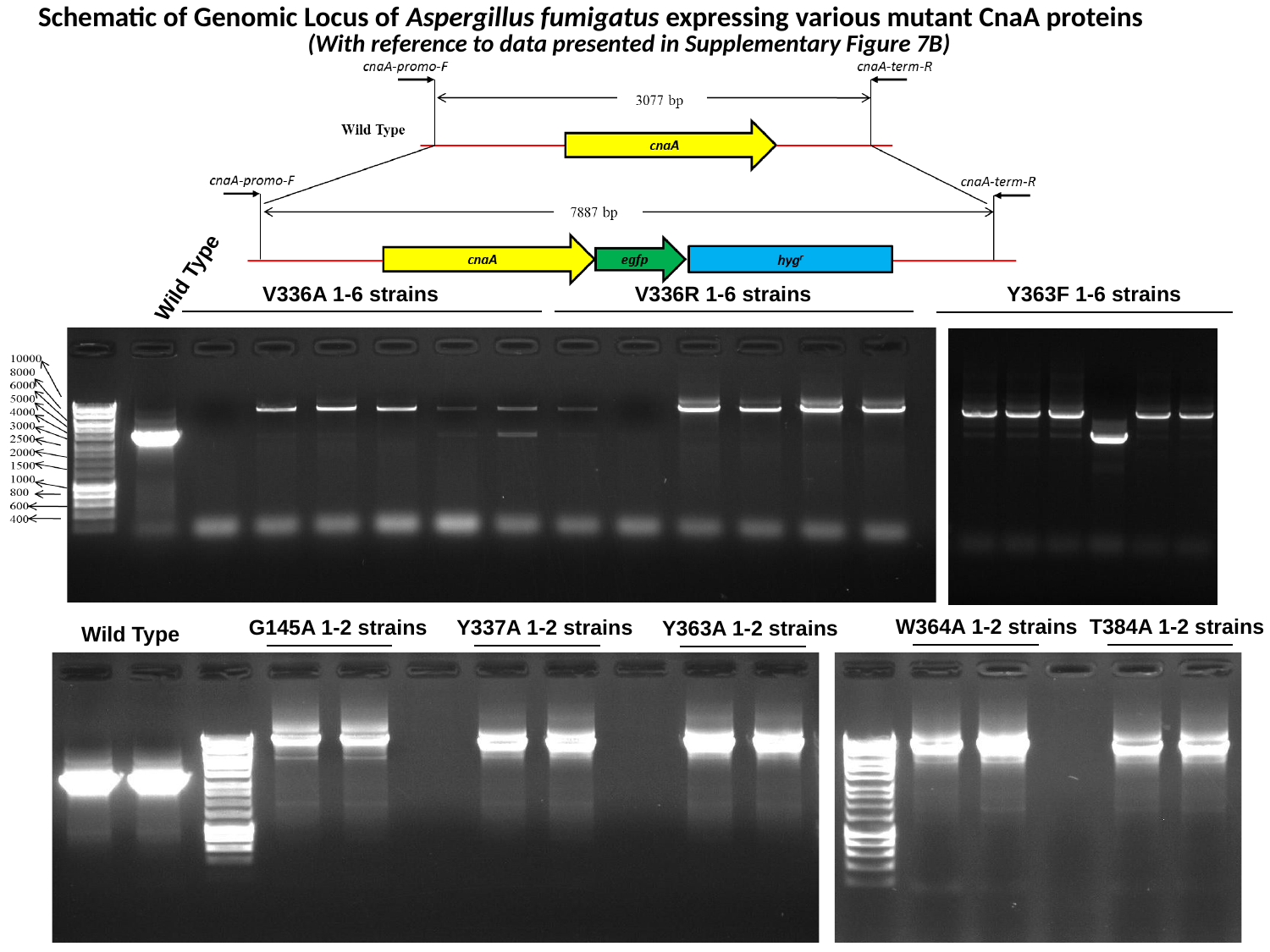

Schematic of Genomic Locus of Aspergillus fumigatus expressing various mutant CnaA proteins
(With reference to data presented in Supplementary Figure 7B)
Wild Type
V336A 1-6 strains
V336R 1-6 strains
Y363F 1-6 strains
T384A 1-2 strains
W364A 1-2 strains
Y337A 1-2 strains
G145A 1-2 strains
Y363A 1-2 strains
Wild Type

## Slide 40
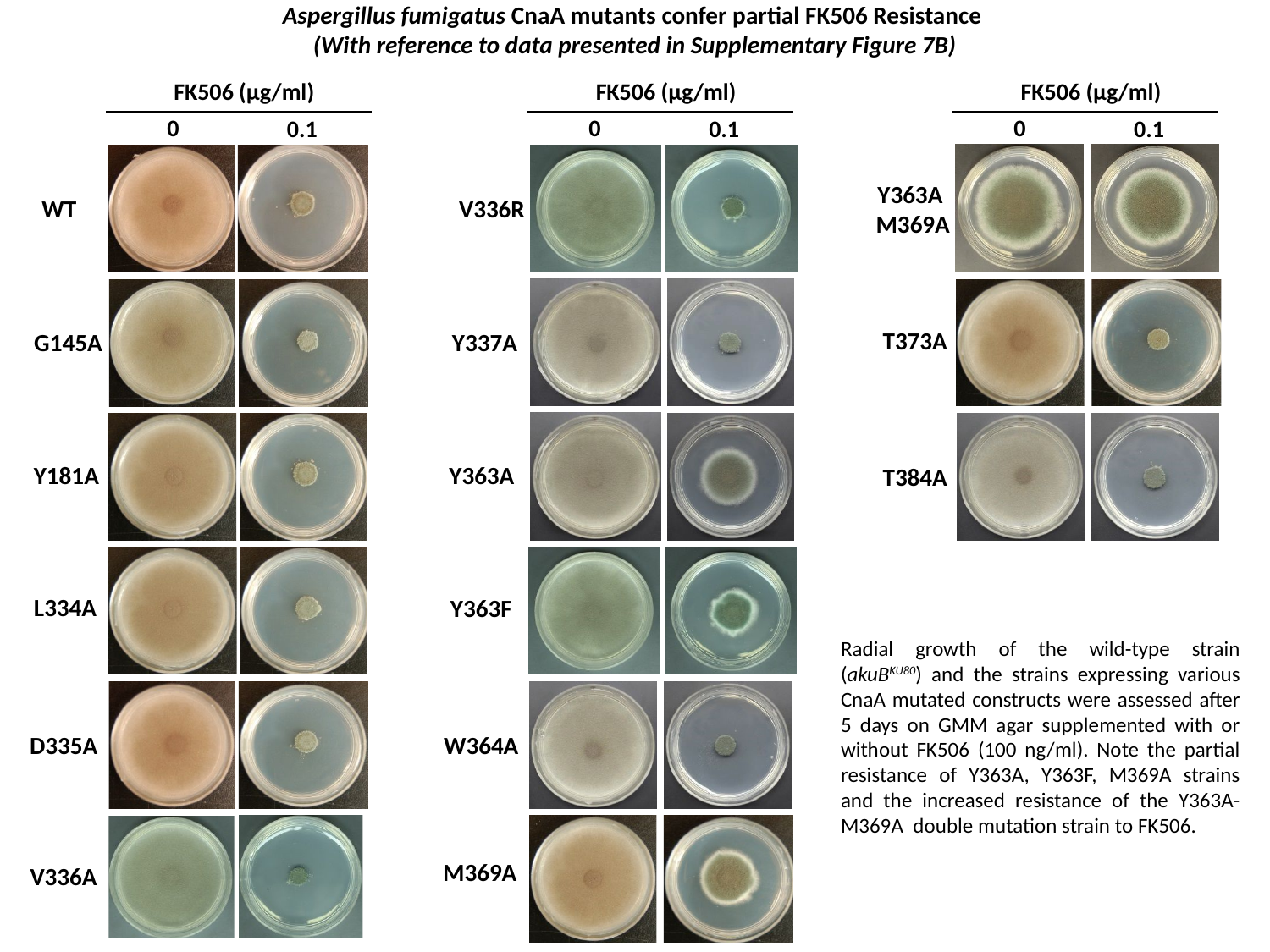

Aspergillus fumigatus CnaA mutants confer partial FK506 Resistance
(With reference to data presented in Supplementary Figure 7B)
FK506 (µg/ml)
0
0.1
WT
G145A
Y181A
L334A
FK506 (µg/ml)
0
0.1
V336R
Y337A
Y363A
Y363F
W364A
M369A
FK506 (µg/ml)
0
0.1
Y363A
M369A
T373A
T384A
Radial growth of the wild-type strain (akuBKU80) and the strains expressing various CnaA mutated constructs were assessed after 5 days on GMM agar supplemented with or without FK506 (100 ng/ml). Note the partial resistance of Y363A, Y363F, M369A strains and the increased resistance of the Y363A-M369A double mutation strain to FK506.
D335A
V336A

## Slide 41
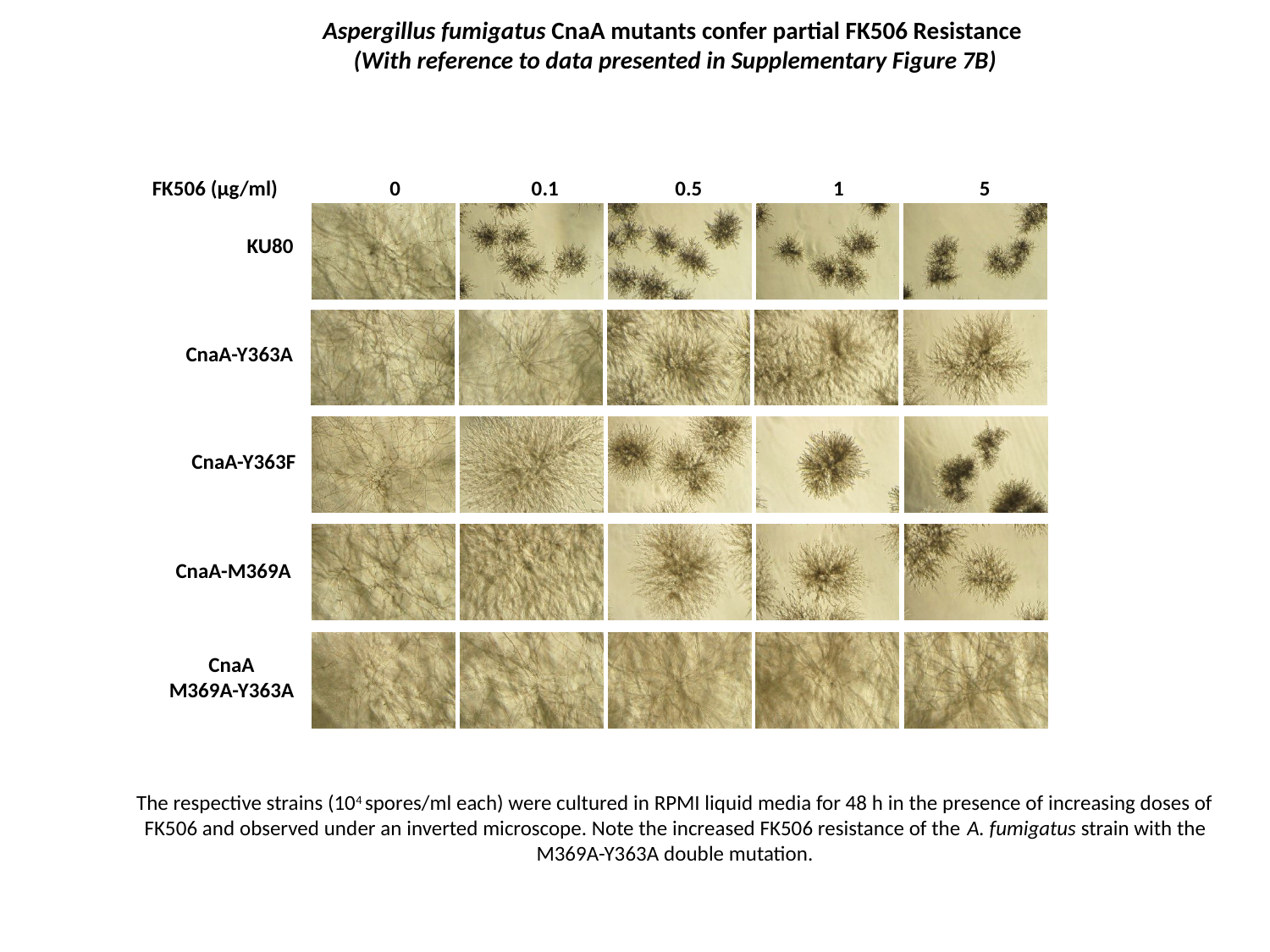

Aspergillus fumigatus CnaA mutants confer partial FK506 Resistance
(With reference to data presented in Supplementary Figure 7B)
FK506 (µg/ml)
0 0.1 0.5 1 5
KU80
CnaA-Y363A
CnaA-Y363F
CnaA-M369A
CnaA
M369A-Y363A
The respective strains (104 spores/ml each) were cultured in RPMI liquid media for 48 h in the presence of increasing doses of FK506 and observed under an inverted microscope. Note the increased FK506 resistance of the A. fumigatus strain with the M369A-Y363A double mutation.

## Slide 42
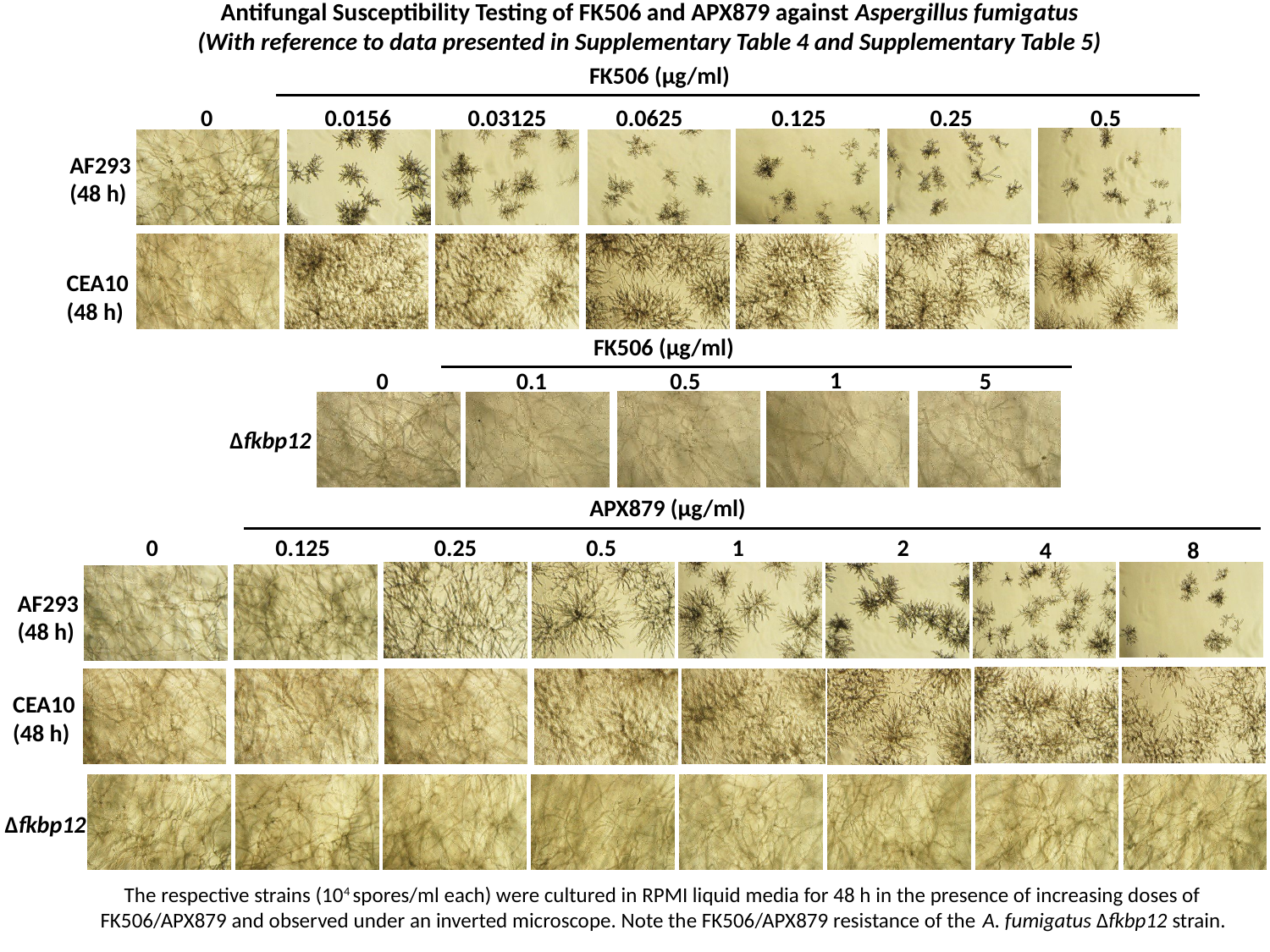

Antifungal Susceptibility Testing of FK506 and APX879 against Aspergillus fumigatus
(With reference to data presented in Supplementary Table 4 and Supplementary Table 5)
FK506 (µg/ml)
0
0.0156
0.03125
0.0625
0.125
0.25
0.5
AF293
(48 h)
CEA10
(48 h)
FK506 (µg/ml)
1
0
0.1
0.5
5
Δfkbp12
APX879 (µg/ml)
0
0.125
0.25
1
2
0.5
4
8
AF293
(48 h)
CEA10
(48 h)
Δfkbp12
The respective strains (104 spores/ml each) were cultured in RPMI liquid media for 48 h in the presence of increasing doses of FK506/APX879 and observed under an inverted microscope. Note the FK506/APX879 resistance of the A. fumigatus Δfkbp12 strain.

## Slide 43
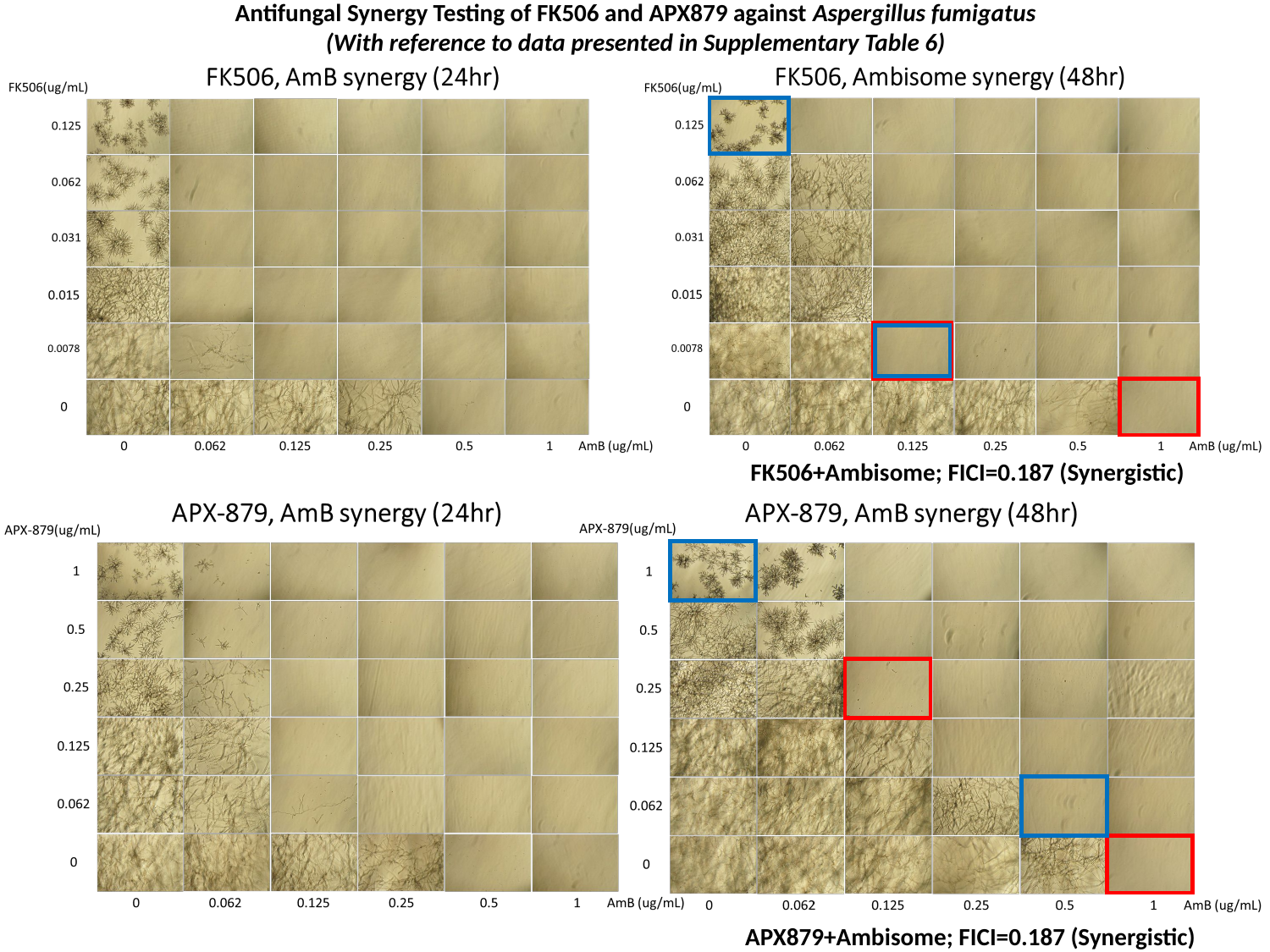

Antifungal Synergy Testing of FK506 and APX879 against Aspergillus fumigatus
(With reference to data presented in Supplementary Table 6)
FK506+Ambisome; FICI=0.187 (Synergistic)
APX879+Ambisome; FICI=0.187 (Synergistic)

## Slide 44
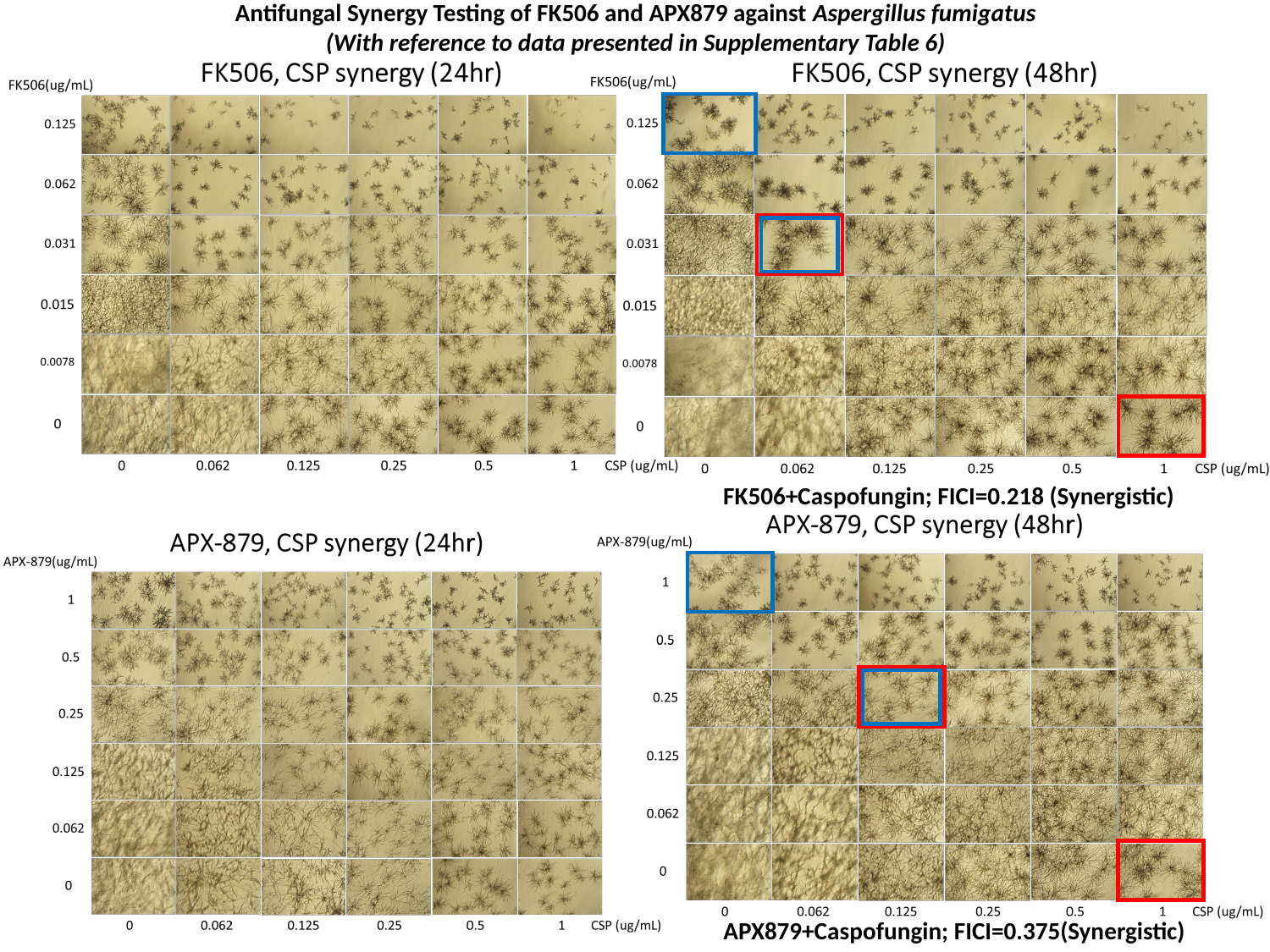

Antifungal Synergy Testing of FK506 and APX879 against Aspergillus fumigatus
(With reference to data presented in Supplementary Table 6)
FK506+Caspofungin; FICI=0.218 (Synergistic)
APX879+Caspofungin; FICI=0.375(Synergistic)

## Slide 45
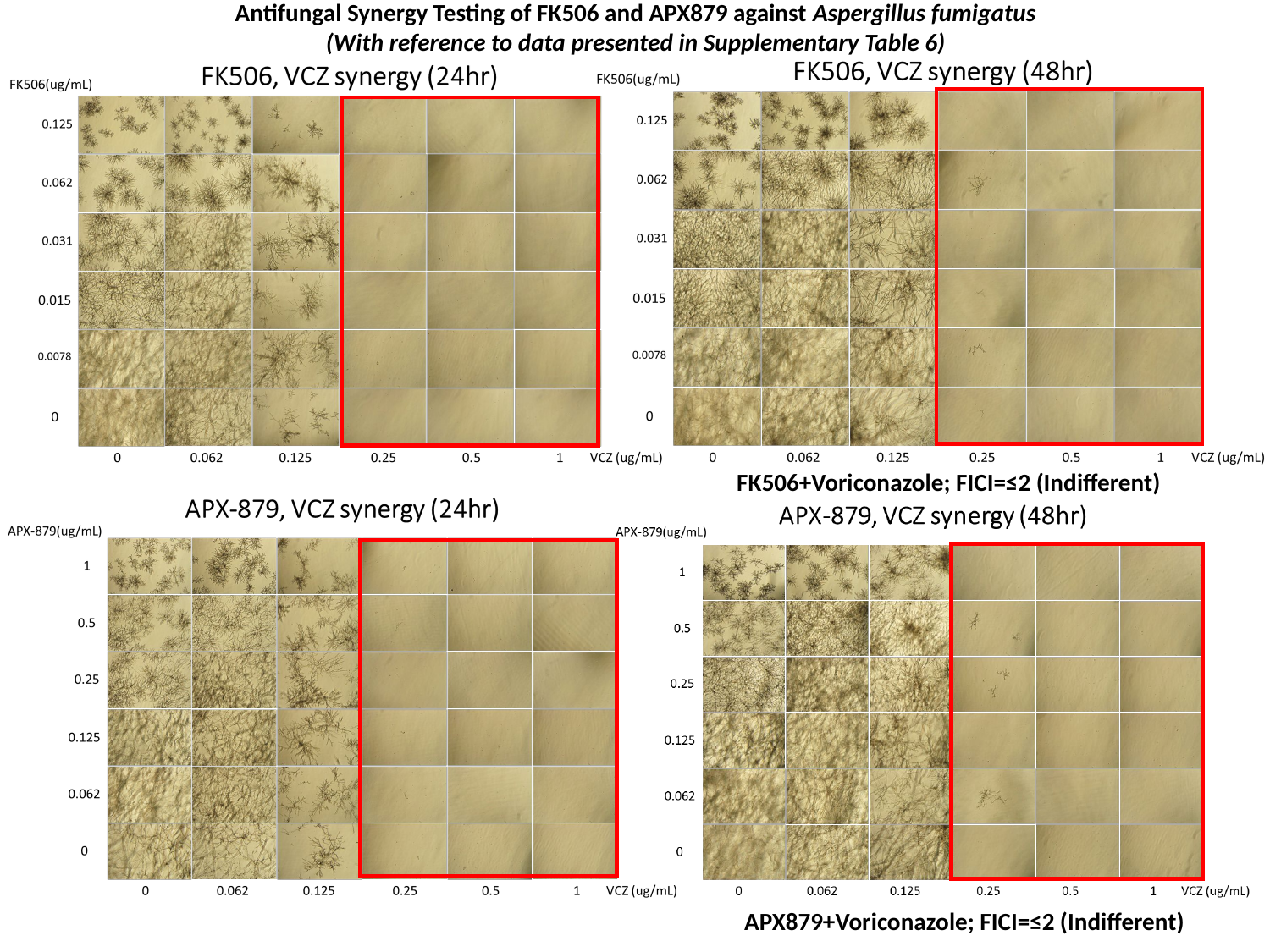

Antifungal Synergy Testing of FK506 and APX879 against Aspergillus fumigatus
(With reference to data presented in Supplementary Table 6)
FK506+Voriconazole; FICI=≤2 (Indifferent)
APX879+Voriconazole; FICI=≤2 (Indifferent)
