# Supplementary material for: Harnessing calcineurin-FK506-FKBP12 crystal structures from invasive fungal pathogens to develop antifungal agents
Source: Nat Commun. 2019 Sep 19;10:4275. doi: 10.1038/s41467-019-12199-1 (PMC6753081; doi:10.1038/s41467-019-12199-1)
Supplement: Supplementary file 8 — Source Data [file 41467_2019_12199_MOESM8_ESM.zip › SOURCE-DATA-NCOMMS-18-31711B-2019/TABLE-OF-CONTENTS-FOR-SOURCE-DATA.docx]

**Description of Table of Contents included in the Source Data File**

**APX879_FK506_800.tar**

The tar file contains the raw data for the 1H and 13C one dimensional NMR spectra of both the APX879 and the FK506 compounds collected on the Duke 800MHz instrument in 100% deuterated DMSO at 25˚C using a triple resonance cryo-cooled probe.

**Human_FKBP12_FK506_gNhsqc.nv**

The Human_FKBP12_FK506_gNhsqc.nv file is the NMRView formatted processed two dimensional 1H/15N HSQC spectra of the 15N/13C labeled Human FKBP12 protein bound to FK506 in a ratio of 2:1 ligand to protein. The data were collected at 25˚C on an Agilent 800MHz instrument with a triple resonance cryo-cooled probe.

**Human_FKBP12_879_gNhsqc.nv**

The Human_FKBP12_879_gNhsqc.nv file is the NMRView formatted processed two dimensional 1H/15N HSQC spectra of the 15N/13C labeled Human FKBP12 protein bound to APX879 in a ratio of 2:1 ligand to protein. The data were collected at 25˚C on an Agilent 800MHz instrument with a triple resonance cryo-cooled probe.

**Fumigatus_FKBP12_FK506_gNhsqc.nv**

The Fumigatus_FKBP12_FK506_gNhsqc.nv file is the NMRView formatted processed two dimensional 1H/15N HSQC spectra of the 15N/13C labeled Fumigatus FKBP12 protein bound to FK506 in a ratio of 2:1 ligand to protein. The data were collected at 25˚C on an Agilent 800MHz instrument with a triple resonance cryo-cooled probe.

**Fumigatus_FKBP12_879_gNhsqc.nv**

The Fumigatus_FKBP12_879_gNhsqc.nv file is the NMRView formatted processed two dimensional 1H/15N HSQC spectra of the 15N/13C labeled Fumigatus FKBP12 protein bound to APX879 in a ratio of 2:1 ligand to protein. The data were collected at 25˚C on an Agilent 800MHz instrument with a triple resonance cryo-cooled probe.

**KEY-RESOURCE-TABLE.docx**

-Includes information on all the constructs, chemicals and strains used in this study

**SOURCE-DATA-Juvvadi-etal-Calcineurin-FKBP12-Structure-Manuscript.pptx**

-Includes all the raw data (PCR confirmations; Strains sequenced to confirm the various AfFKBP12 mutations; Phenotyping and Fluorescence microscopy images) pertaining to Figure 2B and Figure 2C.

-Includes all the raw data (PCR confirmations for the HFKBP12 expression strain; Phenotyping; Fluorescence microscopy images) pertaining to Figure 3C and Figure 3D.

-Includes all the raw data (PCR confirmations for the various HFKBP12 mutants; HFKBP12 mutant strains sequenced to confirm the various mutations; Phenotyping) pertaining to Figure 4A.

-Includes the NMR data (1H-NMR and 13C-NMR) for APX879 and FK506. The high resolution mass spectra (HRMS) for APX879 is included. These data pertain to Figure 6A.

-Includes the Cell Flow Gating data pertaining to Figure 6C, Figure 6D and Figure 6E.

-Includes the Western analysis data pertaining to Supplementary Figure 1G.

-Includes the Western analysis data pertaining to Supplementary Figure 5B.

-Includes all the raw data (PCR confirmations; Strains sequenced to confirm the various CnaA mutations; Phenotyping) pertaining to Figure 7B.

-Includes the raw antifungal susceptibility testing data included in Supplementary Table 4 and Supplementary Table 4.

-Includes the raw antifungal synergy testing data for FK506 and APX879 in combination with Amphotericin, Caspofungin, and Voriconazole for the data included in Supplementary Table 6.

**SOURCE-DATA-FOR-NMR.xlsx**

-Includes the 3 raw NMR data files for Figure 2A, Supplementary Figure 2 and Supplementary Figure 6.

**All the HFKBP12 mutant strains sequencing files are included:**

HFKBP12-T86R-Strains-Sequenced.pdf

HFKBP12-T86R-H88F-Strains-Sequenced.pdf

HFKBP12-R41V-K45E-Strains-Sequenced.pdf

HFKBP12-M50Q-Strains-Sequenced.pdf

HFKBP12-K53T-Q54G-Strains-Sequenced.pdf

HFKBP12-K48Q-Strains-Sequenced.pdf

HFKBP12-K48Q-M50Q-Strains-Sequenced.pdf

HFKBP12-H88F-Strains-Sequenced.pdf

**All the AfFKBP12 mutant strains sequencing files are included:**

AfFkbp12-R55E-Strain-Sequenced.pdf

AfFkbp12-Q50M-Strains-Sequenced.pdf

AfFkbp12-F88H-Strain-Sequenced.pdf

AfFkbp12-F22T-Strain-Sequenced.pdf
