# Supplementary figures and images for: Harnessing calcineurin-FK506-FKBP12 crystal structures from invasive fungal pathogens to develop antifungal agents
Source: Nat Commun. 2019 Sep 19;10:4275. doi: 10.1038/s41467-019-12199-1 (PMC6753081; doi:10.1038/s41467-019-12199-1)

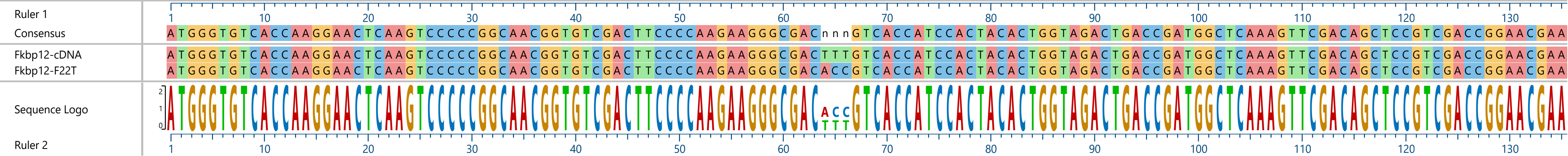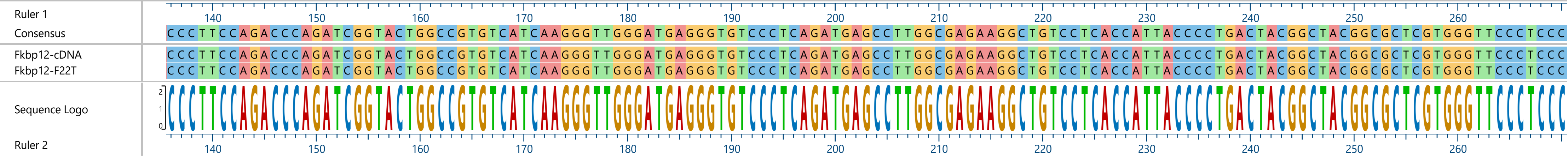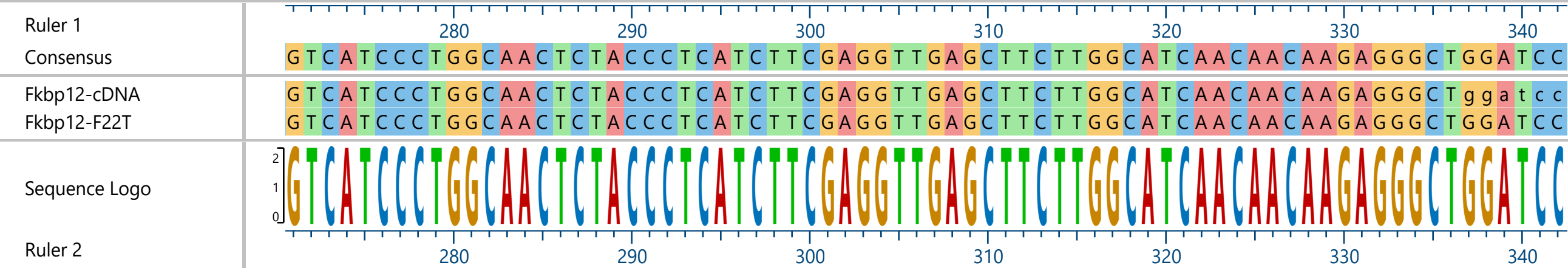

Supplement: Supplementary file 8 — Source Data [file 41467_2019_12199_MOESM8_ESM.zip › SOURCE-DATA-NCOMMS-18-31711B-2019/AfFkbp12-F22T-Strain-Sequenced.pdf]

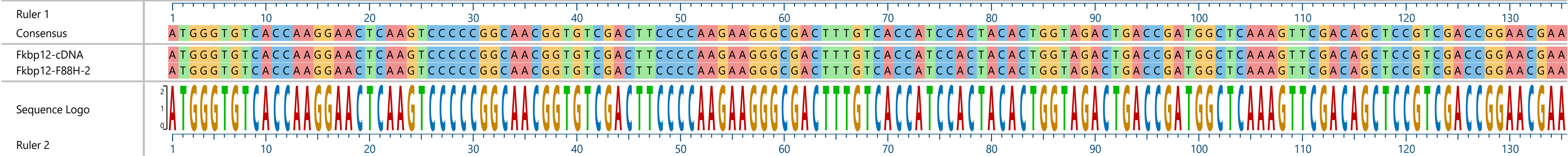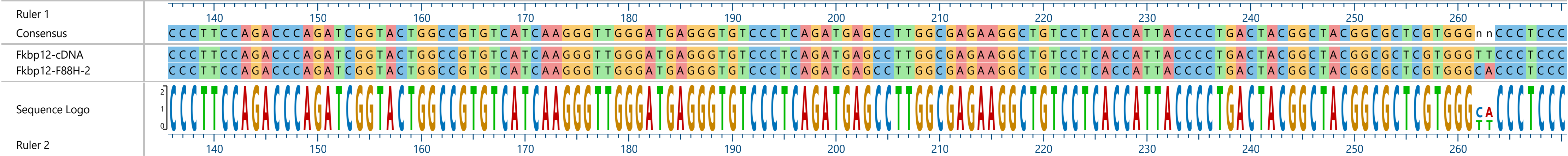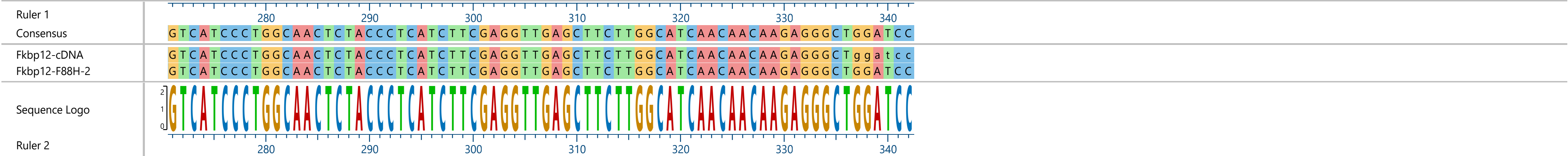

Supplement: Supplementary file 8 — Source Data [file 41467_2019_12199_MOESM8_ESM.zip › SOURCE-DATA-NCOMMS-18-31711B-2019/AfFkbp12-F88H-Strain-Sequenced.pdf]

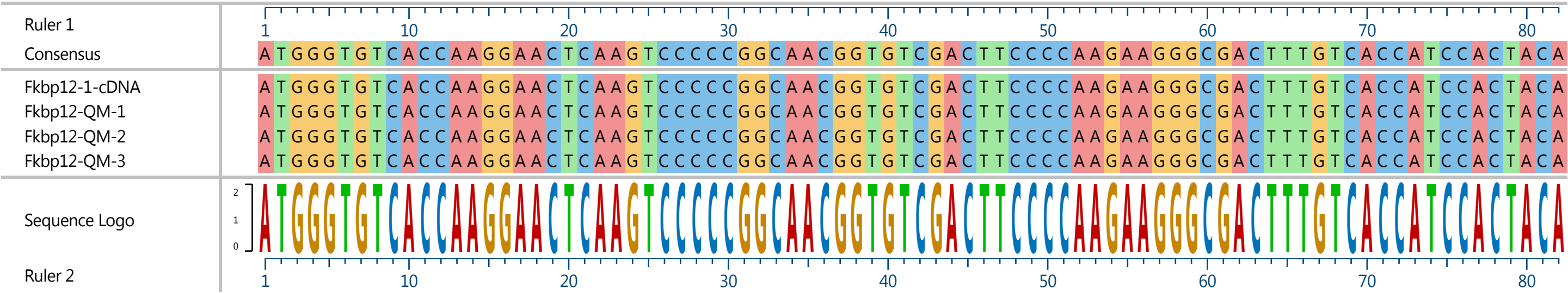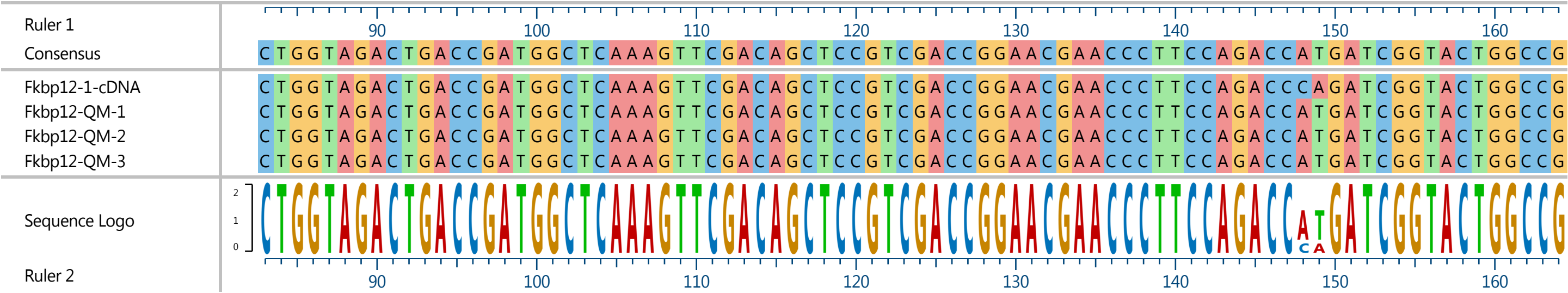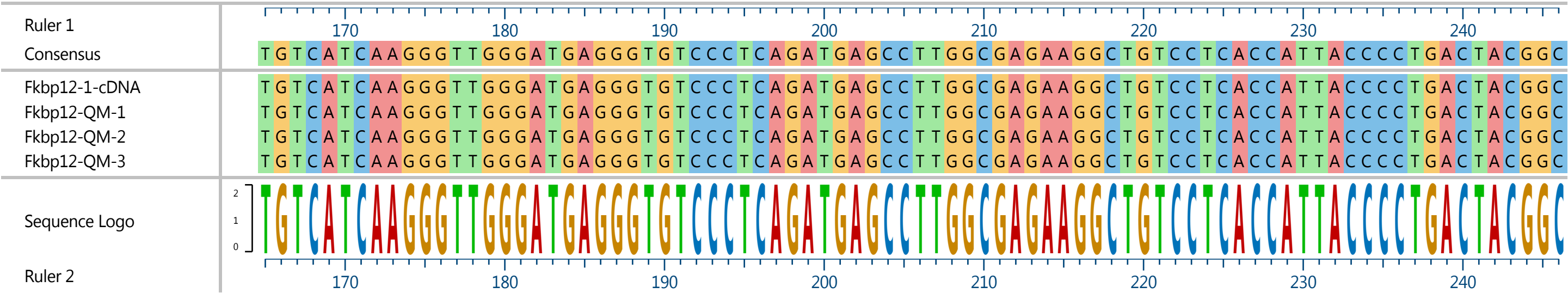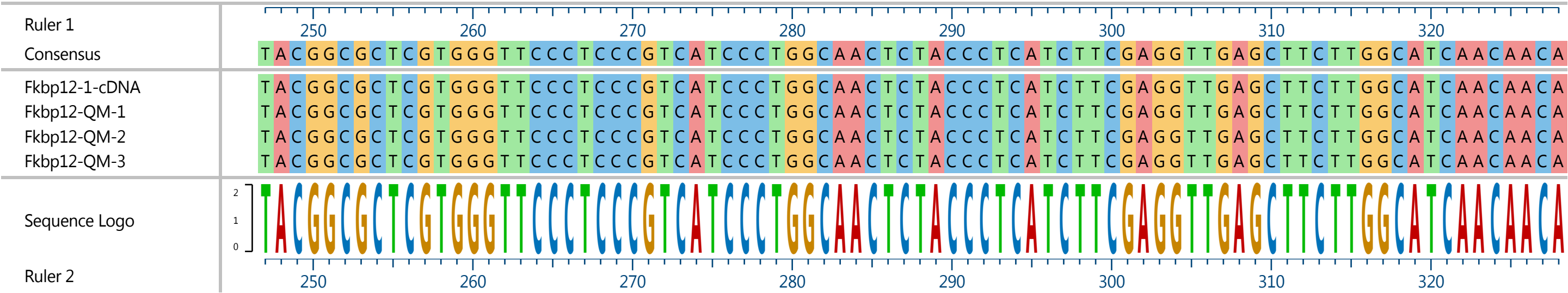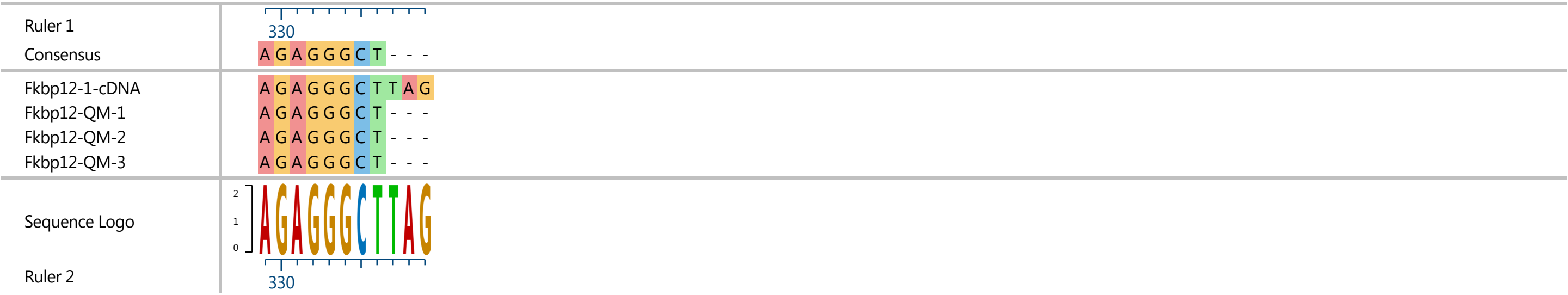

Supplement: Supplementary file 8 — Source Data [file 41467_2019_12199_MOESM8_ESM.zip › SOURCE-DATA-NCOMMS-18-31711B-2019/AfFkbp12-Q50M-Strains-Sequenced.pdf]

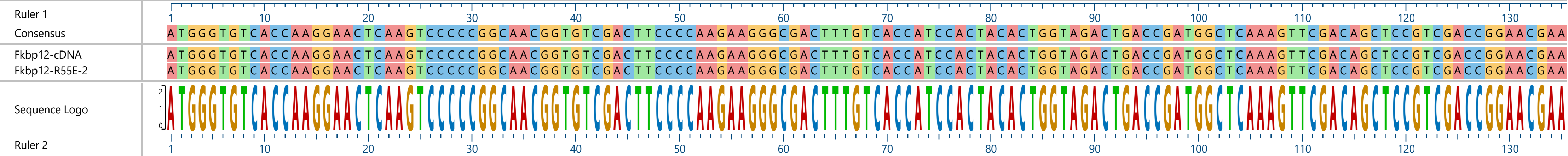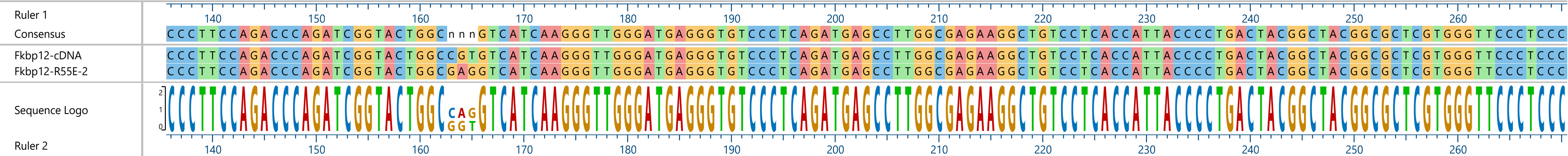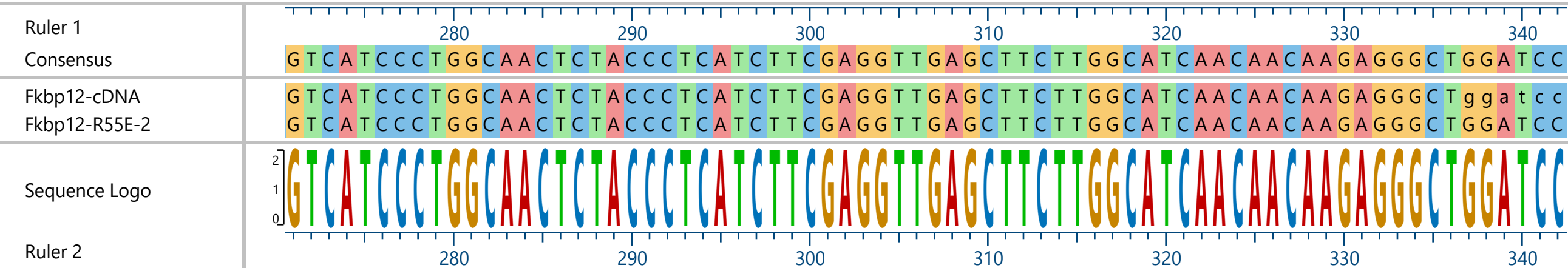

Supplement: Supplementary file 8 — Source Data [file 41467_2019_12199_MOESM8_ESM.zip › SOURCE-DATA-NCOMMS-18-31711B-2019/AfFkbp12-R55E-Strain-Sequenced.pdf]

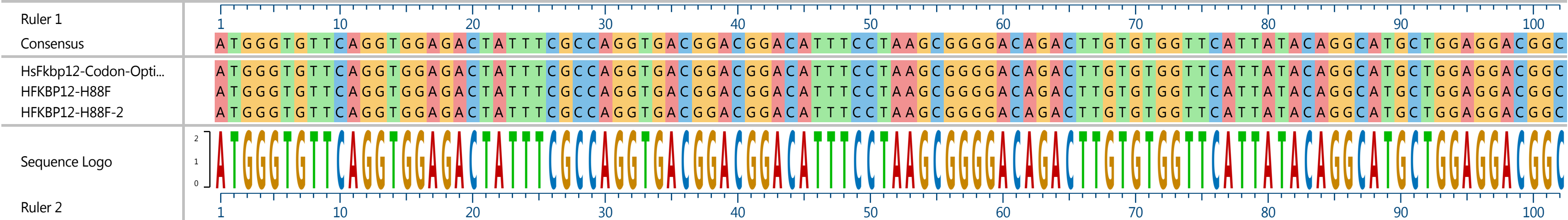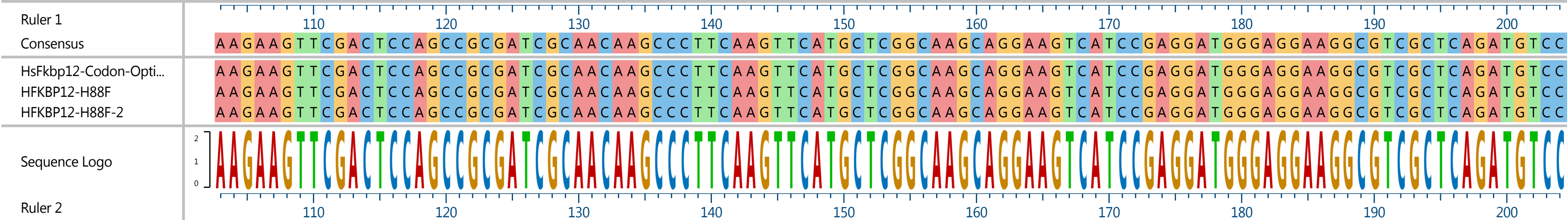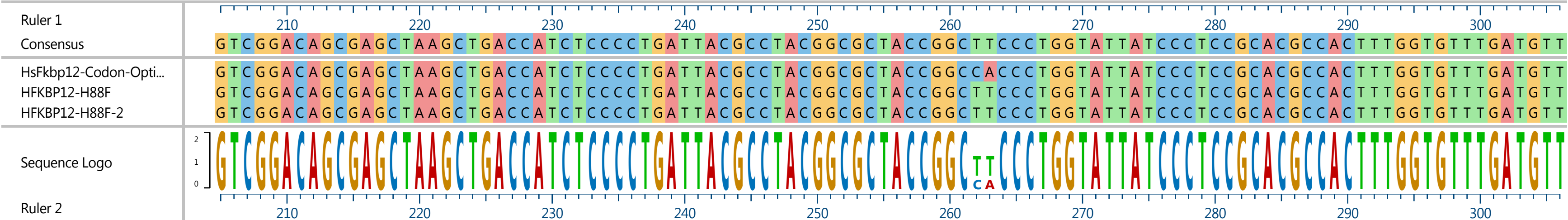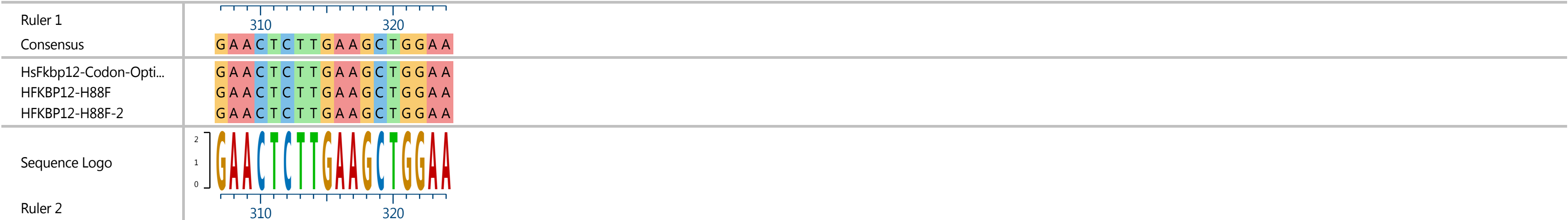

Supplement: Supplementary file 8 — Source Data [file 41467_2019_12199_MOESM8_ESM.zip › SOURCE-DATA-NCOMMS-18-31711B-2019/HFKBP12-H88F-Strains-Sequenced.pdf]

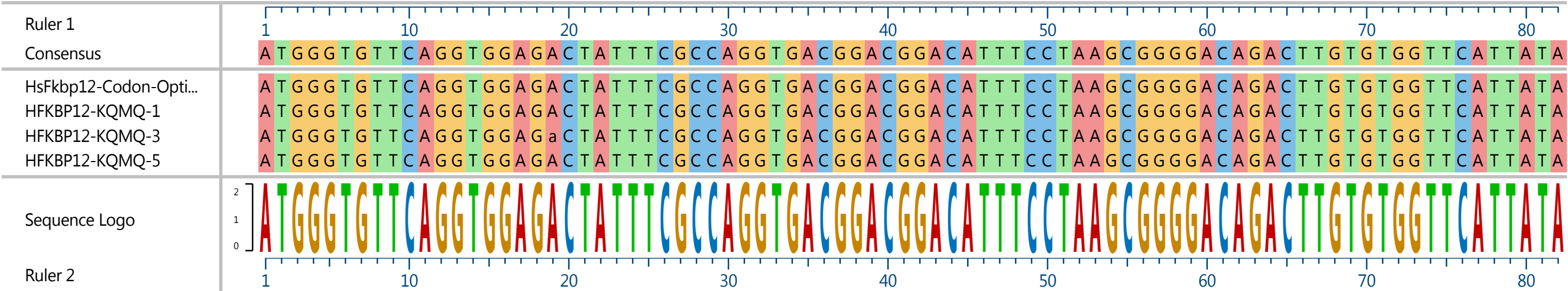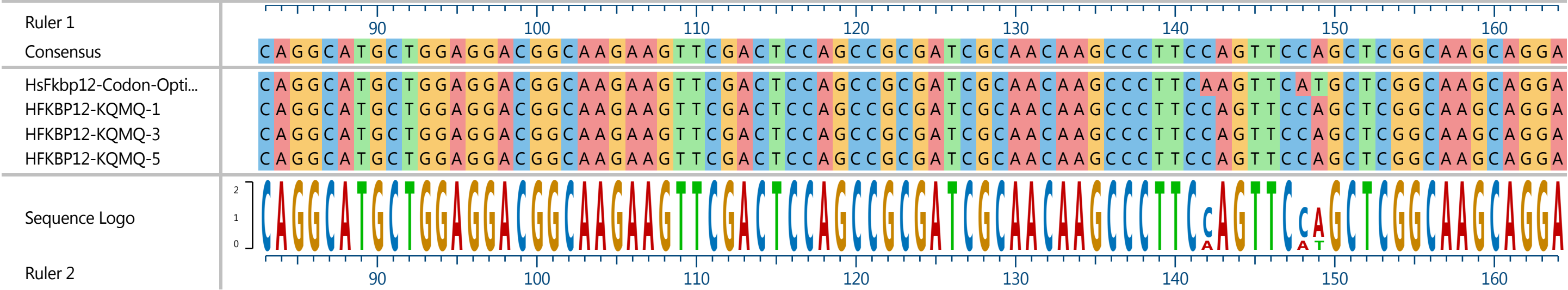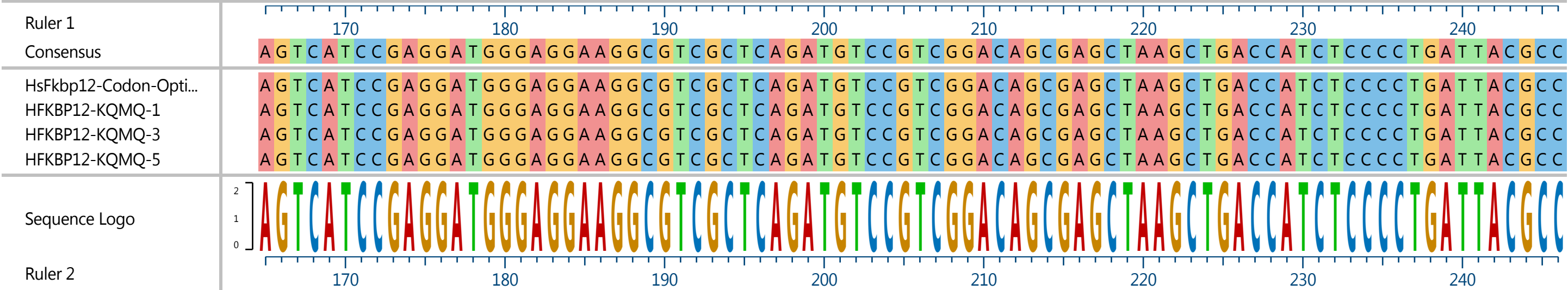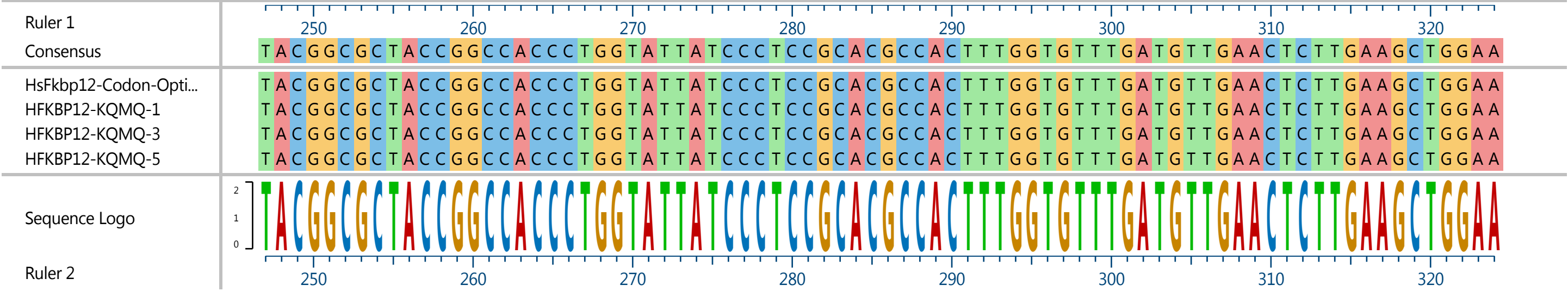

Supplement: Supplementary file 8 — Source Data [file 41467_2019_12199_MOESM8_ESM.zip › SOURCE-DATA-NCOMMS-18-31711B-2019/HFKBP12-K48Q-M50Q-Strains-Sequenced.pdf]

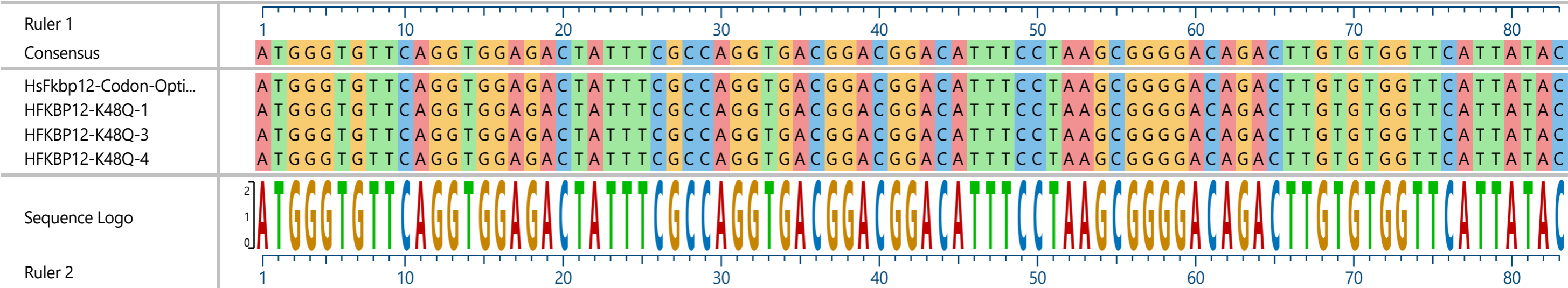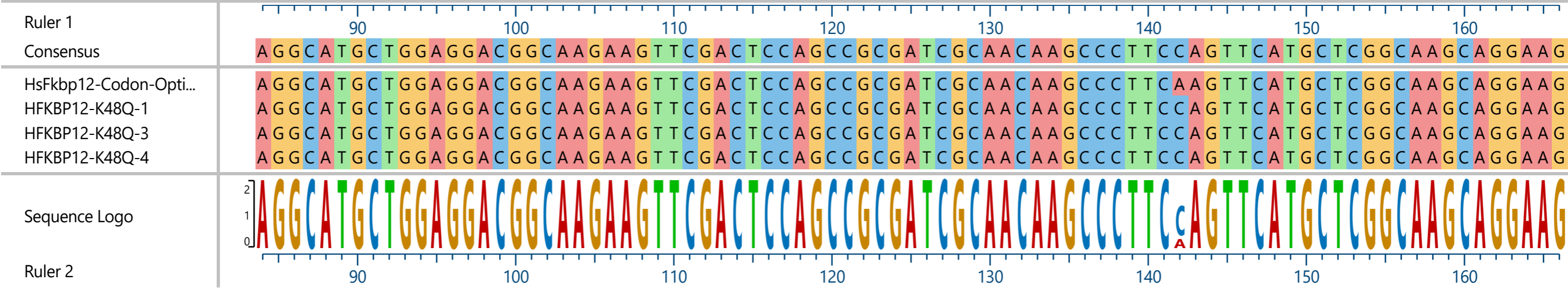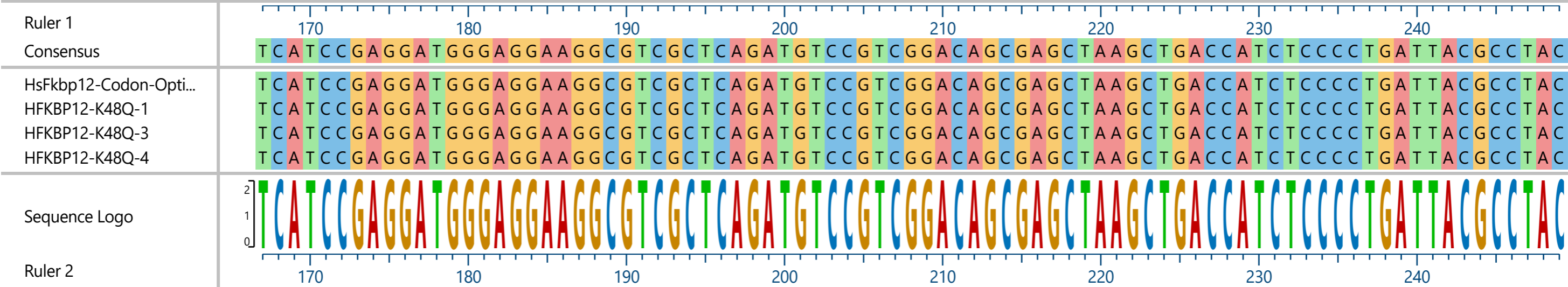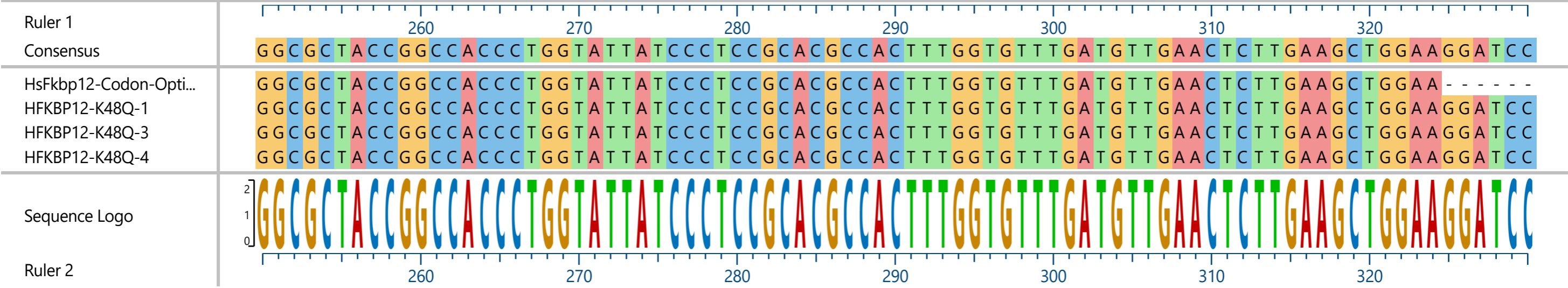

Supplement: Supplementary file 8 — Source Data [file 41467_2019_12199_MOESM8_ESM.zip › SOURCE-DATA-NCOMMS-18-31711B-2019/HFKBP12-K48Q-Strains-Sequenced.pdf]

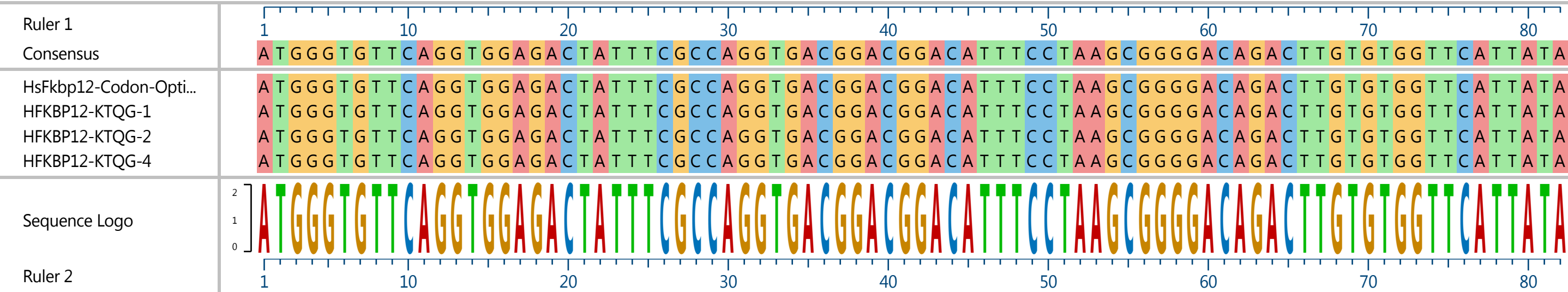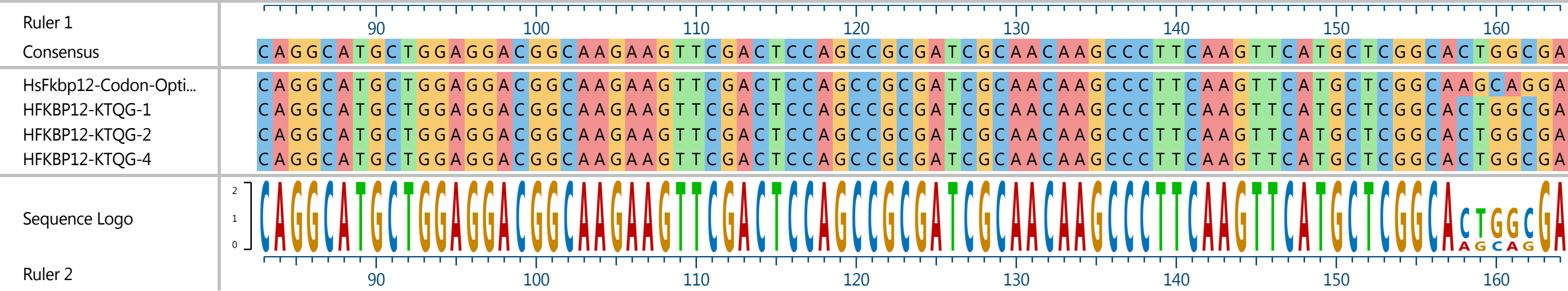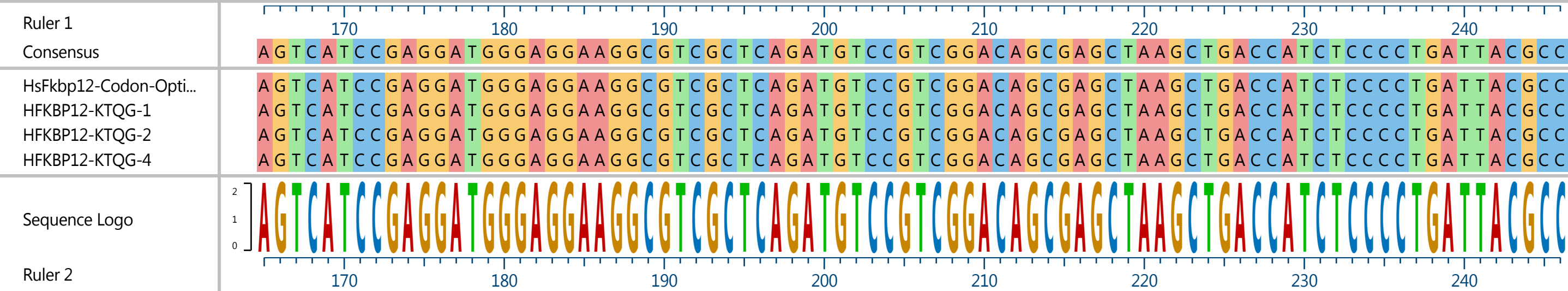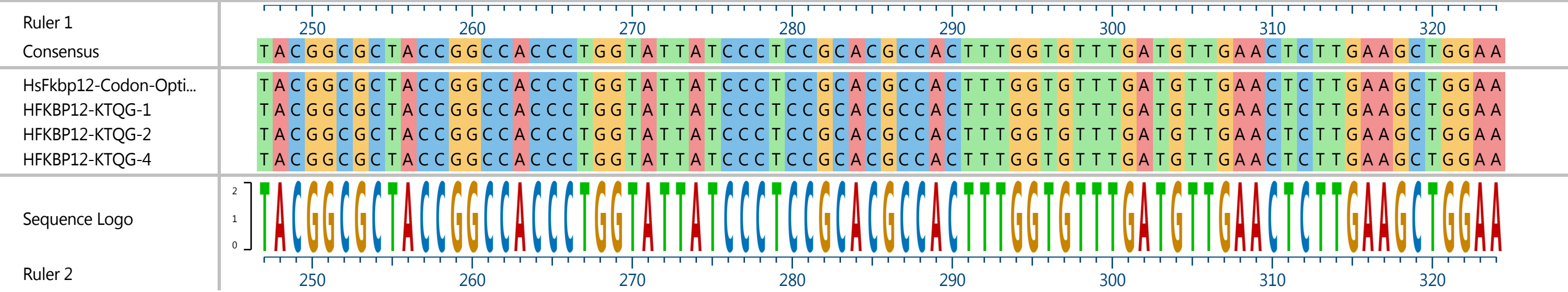

Supplement: Supplementary file 8 — Source Data [file 41467_2019_12199_MOESM8_ESM.zip › SOURCE-DATA-NCOMMS-18-31711B-2019/HFKBP12-K53T-Q54G-Strains-Sequenced.pdf]

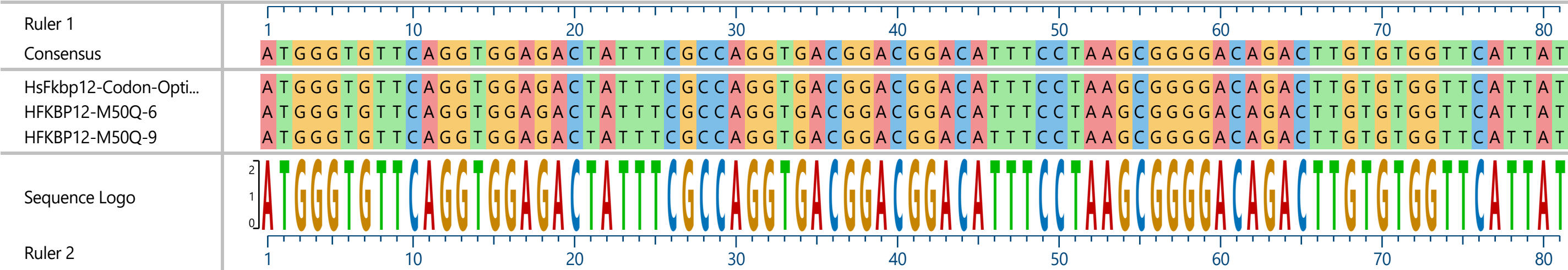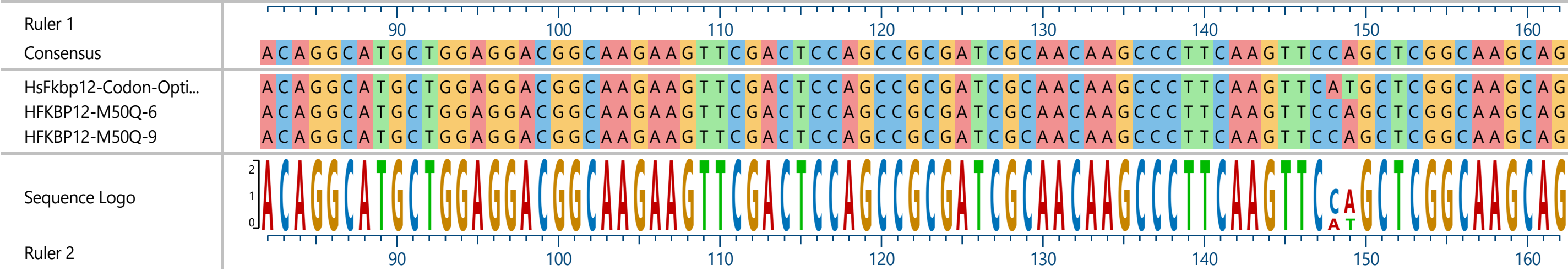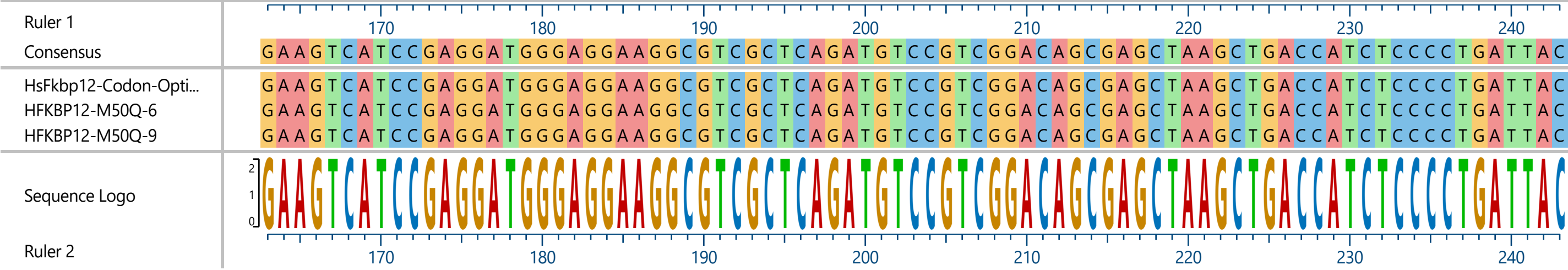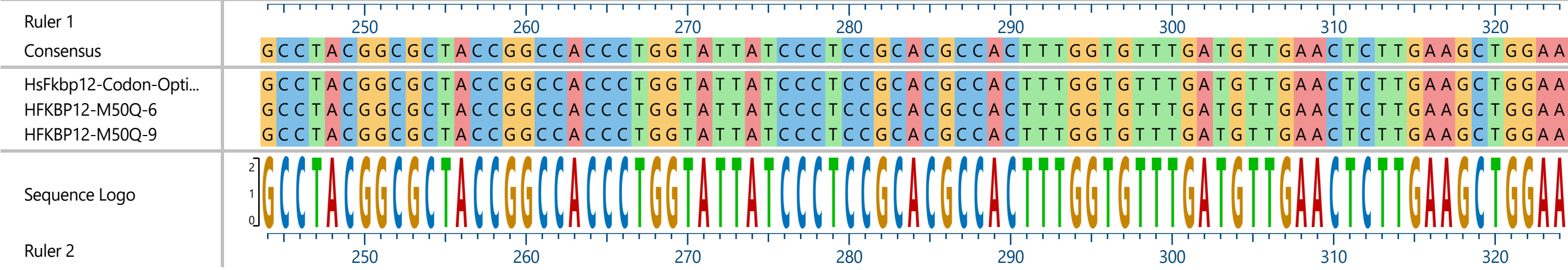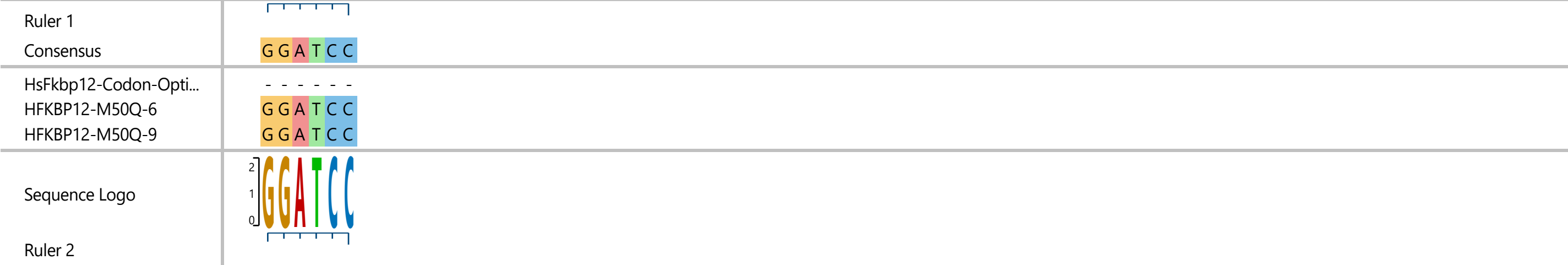

Supplement: Supplementary file 8 — Source Data [file 41467_2019_12199_MOESM8_ESM.zip › SOURCE-DATA-NCOMMS-18-31711B-2019/HFKBP12-M50Q-Strains-Sequenced.pdf]

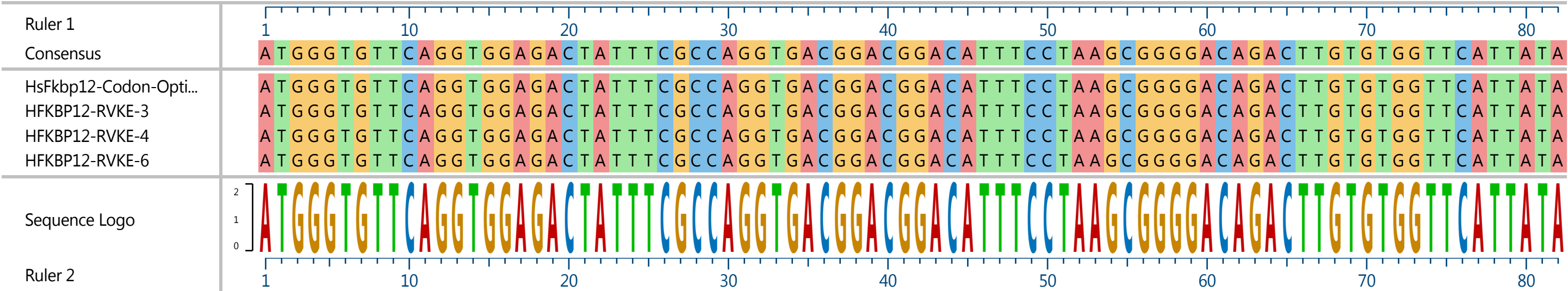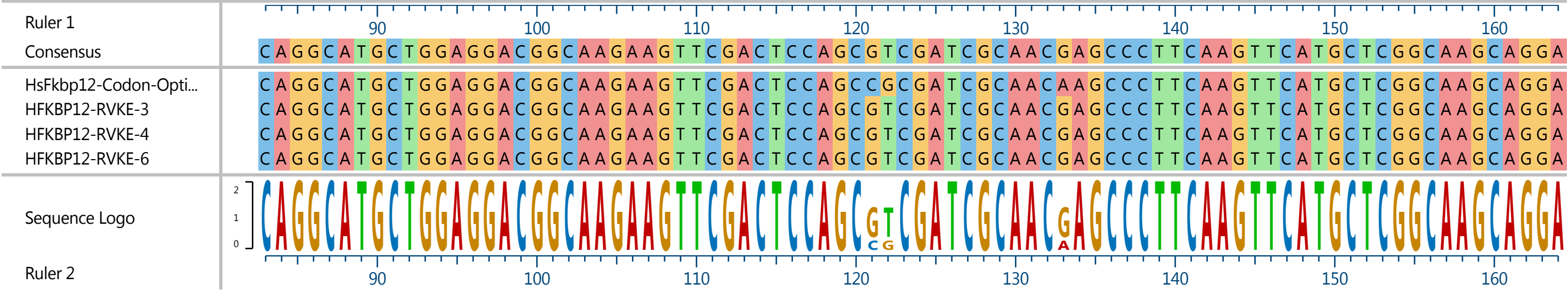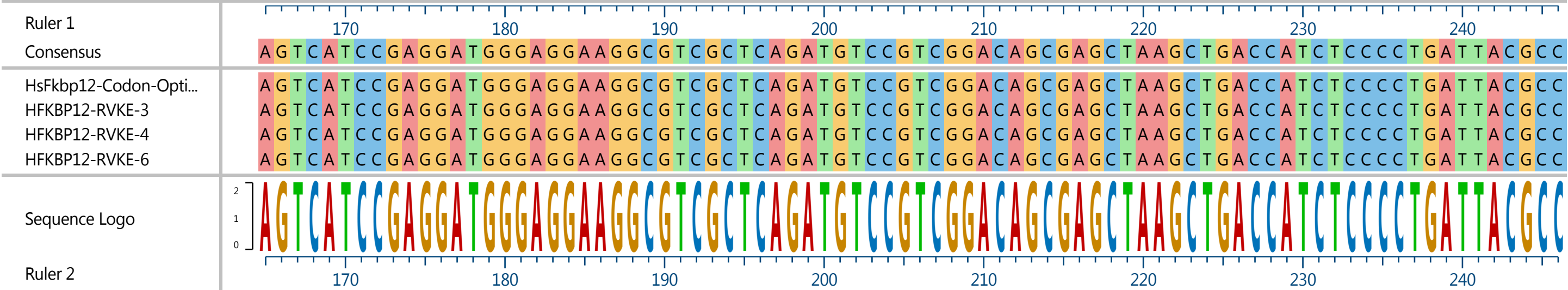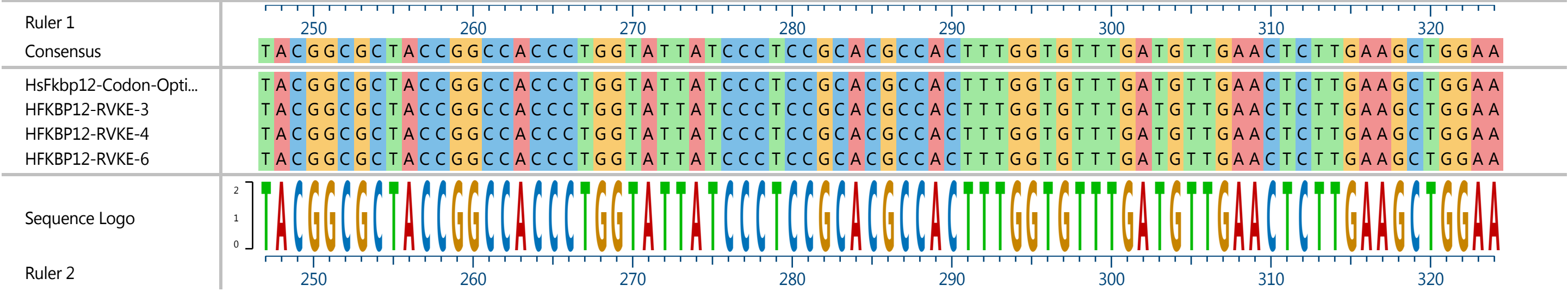

Supplement: Supplementary file 8 — Source Data [file 41467_2019_12199_MOESM8_ESM.zip › SOURCE-DATA-NCOMMS-18-31711B-2019/HFKBP12-R41V-K45E-Strains-Sequenced.pdf]

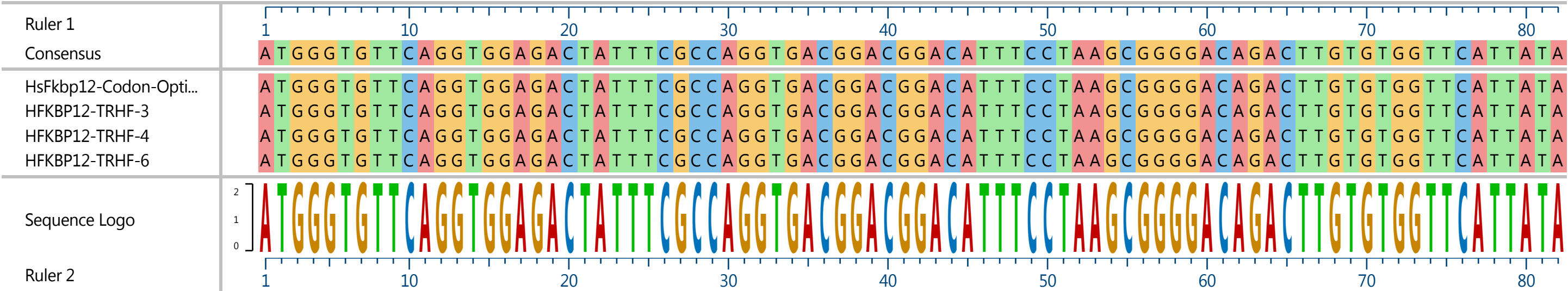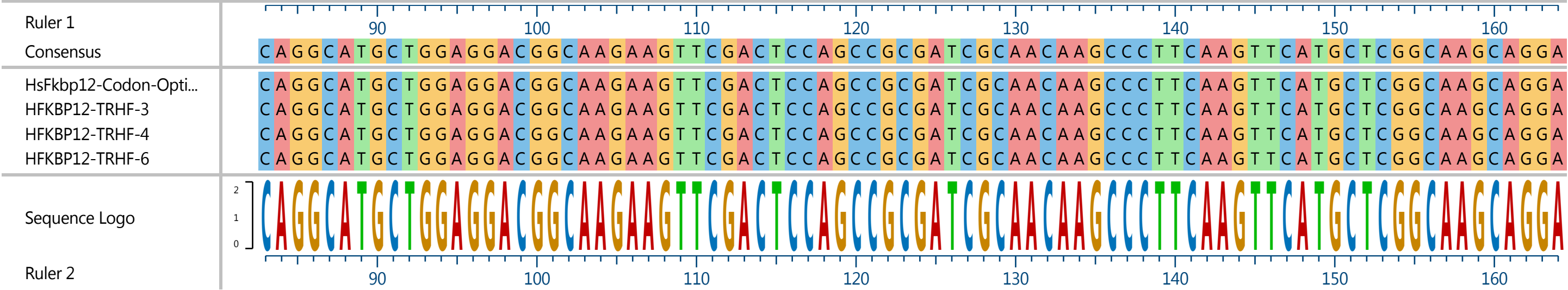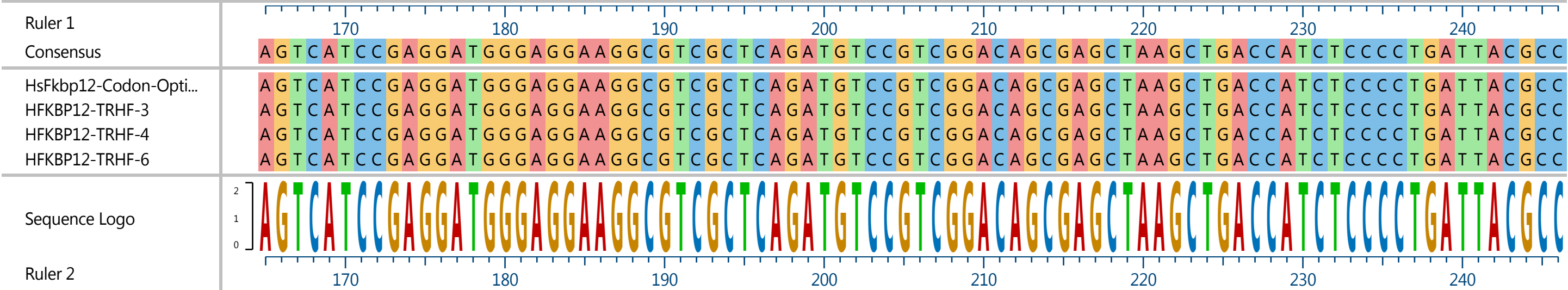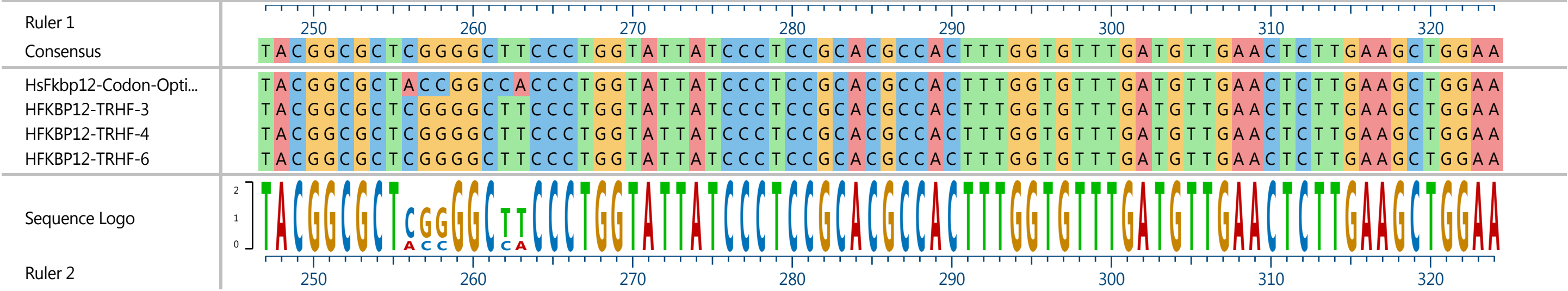

Supplement: Supplementary file 8 — Source Data [file 41467_2019_12199_MOESM8_ESM.zip › SOURCE-DATA-NCOMMS-18-31711B-2019/HFKBP12-T86R-H88F-Strains-Sequenced.pdf]

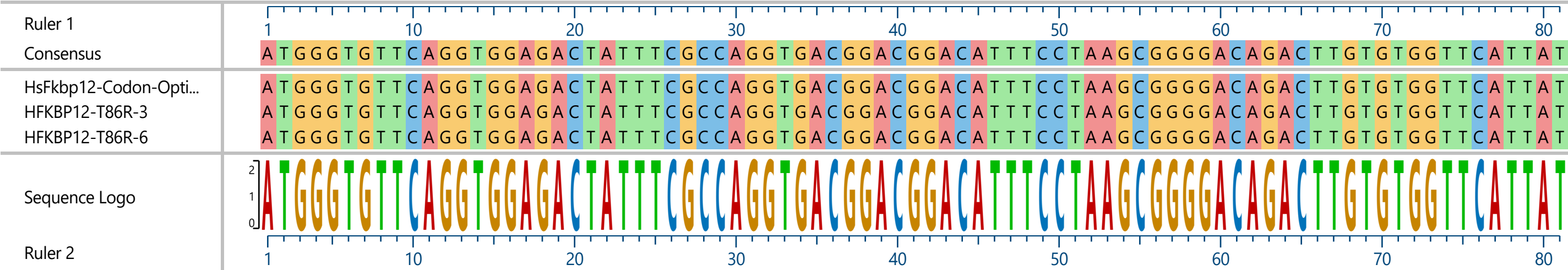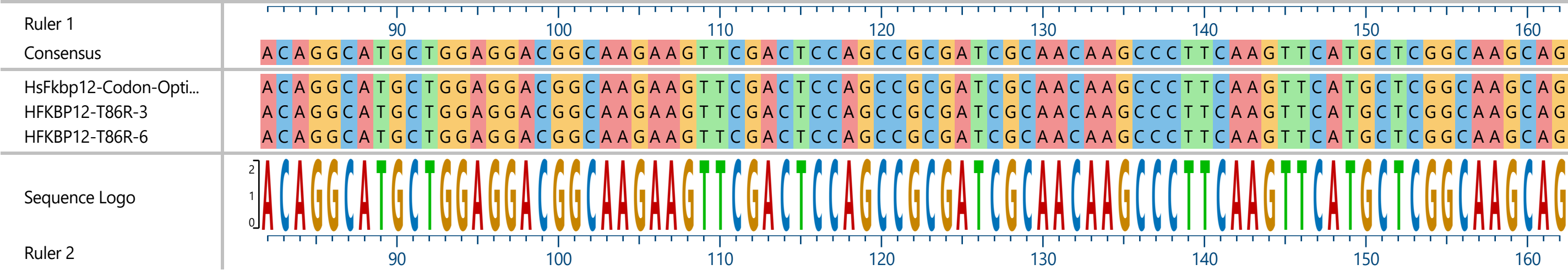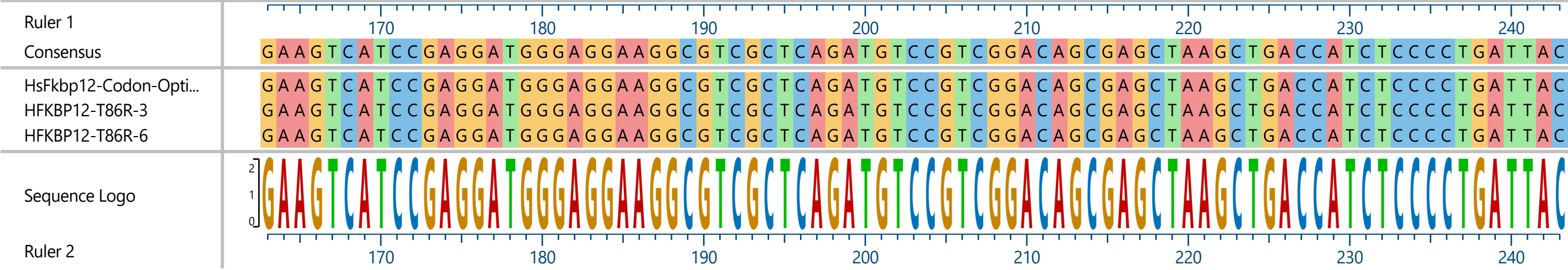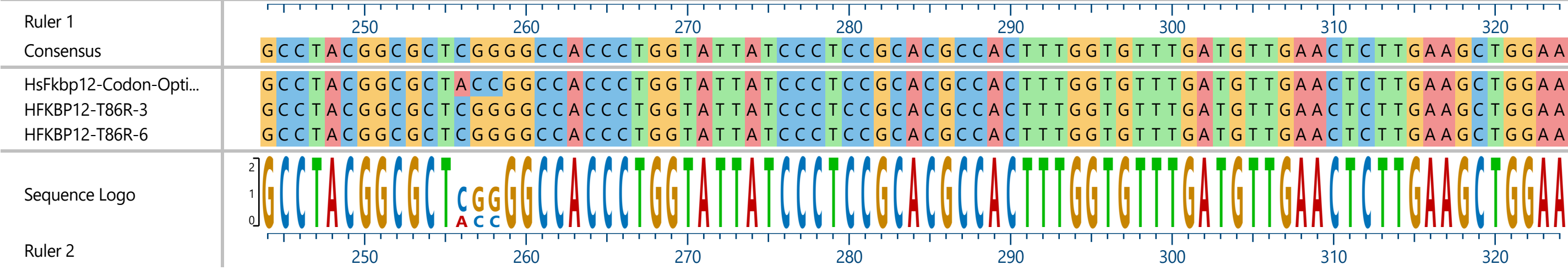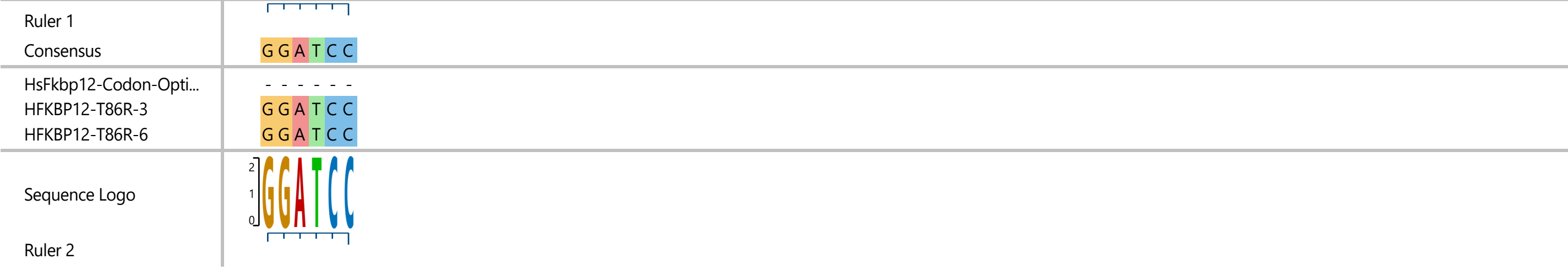

Supplement: Supplementary file 8 — Source Data [file 41467_2019_12199_MOESM8_ESM.zip › SOURCE-DATA-NCOMMS-18-31711B-2019/HFKBP12-T86R-Strains-Sequenced.pdf]
